# Supplementary material for: Dynamic chromatin architecture of the porcine adipose tissues with weight gain and loss
Source: Nat Commun. 2023 Jun 12;14:3457. doi: 10.1038/s41467-023-39191-0 (PMC10258790; doi:10.1038/s41467-023-39191-0)
Supplement: Supplementary file 1 — Supplementary Information [file 41467_2023_39191_MOESM1_ESM.pdf]

Supplementary Information

Supplementary Note 1 Transcriptome alteration in distinct AT response to weight gain and loss

To characterize the transcriptomic differences among AT responses to changes of body weight, we sequenced 245 paired-end rRNA-depleted RNA-seq libraries from the AT samples (~11.84 gigabases [Gb] of high-quality sequences per library; ~3.30 terabases [Tb] total) (**Supplementary Data 1**). The transcriptomic variations of protein-coding genes and other two transcripts with essential regulatory roles (*i.e.*, long noncoding RNAs and transcripts of uncertain coding potential) **(Supplementary Fig. 6)** highlight that the differences between ATs (especially between SAT and VATs) extend beyond weight changes, and indicate that changes in chromatin architecture are correlated with shifts in transcriptional activity. Analysis of differentially expressed genes (DEGs) identified an average of ~1409 DEGs between pairwise ATs, especially between SAT and VATs (~1728 DE genes). An average of ~1322 DEGs were identified between the WG and NC groups, while ~1379 DEGs were identified between the WG and WL groups for each AT (**Supplementary Data 2**).

We performed functional enrichment analysis for differentially expressed protein-coding genes. For a given AT, genes (~ 643) that were up-regulated in WG compared to NC were primarily involved in immune and inflammation responses (such as 'lymphocyte activation') and fibrosis (such as 'extracellular matrix organization'). This reflects the increased inflammation in ATs during obesity development[1,2] **(Supplementary Fig. 3)**. Consistent with the sustained depression of the metabolic ratio after dietary restrictions[3], genes (~706) that are down-regulated in ATs of WL compared to WG were mainly involved in metabolism (such as 'fatty acid metabolism' and 'mitochondrial transmembrane transport') **(Supplementary Fig. 3)**. Under three nutritional conditions, genes that specifically were up-regulated in VATs (~804) and SAT (~920) were separately involved in immune and inflammatory processes (such as 'regulation of T cell cytokine production' and 'chemotaxis') and lipid metabolism processes (such as 'lipid biosynthetic process')[4]. **(Supplementary Fig. 7)**.

Supplementary Note 2 Compartmental arrangements contribute to distinct ATs physiologies under different nutritional conditions

In addition to the analysis of compartment frequency changes between ATs or groups, we used a more detailed method to quantify these changes. We defined the sets of SAT-restricted highly accessible regions (179.48 Mb; or ~7.92% of the genome) under different nutritional conditions that specifically have A compartment status (*i.e.*, A/B switches) or significantly higher A-B index against its counterpart in VATs (Δ A-B index > 0.75, Student's *t*-test, FDR < 0.01) **(Supplementary Fig. 9–11).** We found that genes (~331) located within these highly accessible SAT-restricted regions tended to show increased expression levels in SATs compared to those in VATs (median fold change = 1.206, $P = 8.12\times10^{-13}$, Wilcoxon rank-sum test) (**Supplementary Fig. 12**). Functional enrichment analysis highlighted the putative functions of genes located in SAT- and RAD- restricted active regions against GOM. Both are related to developmental processes (typically, 'embryonic morphogenesis' and 'regionalization') **(Supplementary Fig. 13)** and indicate the higher potential of hyperplasia for SAT compared to VATs during obesity development[4,5]. Thirty-seven genes are involved in 'mesenchymal cell differentiation' **(Supplementary Fig. 13)**, a major source of adipocyte progenitors, are among these differentially compartmentalized and expressed genes (such as *TBX1*, *FOXC2*, and *PAX1*).

In contrast, only 27.13 Mb (~1.20% of the genome) regions of a given AT exhibited specifically distinct compartmental status or had significant differences in the A-B index among nutritional conditions, which also influence the expression of embedded genes (**Supplementary Fig. 11, 12**). For a given AT, compared with the NC group, genes (~225) located in WG-restricted highly accessible regions (and which are thus more active in transcription) were primarily involved in inflammation and immune response processes (*e.g.*, 'TNFα mediated signaling pathway,' 'chemotaxis' and 'lymphocyte differentiation') and cellular biogenesis (*e.g.*, 'positive regulation of cellular component biogenesis') **(Supplementary Fig. 14),** which reflects increased inflammation in ATs associated with weight gain. We also found that representative inflammatory markers were among this set of genes, such as

EGR1 (an inflammatory TF and hypoxia-sensitive marker)[6,7], *CD14* (a macrophage molecule marker that modulates the inflammatory activity and insulin resistance[8]), and *TNFSF12* (a pro-inflammatory TNF system cytokines[9]) **(Supplementary Fig. 15)**.

Supplementary Note 3 Variable TAD boundaries of ATs with weight gain and loss

We partitioned the genome into 4795 - 5469 TADs[10] (median sizes of 448 kb, occupied ~98.85% of the genome) (**Supplementary Fig. 17a)**. However, the TAD boundaries were mostly shared among ATs (~80% are colocalized within 100 kb) **(Supplementary Fig. 17e)**, and the more profound divergence of the local spatial context (measured by the insulation score [IS] and the local boundary score [LBS] of the merged unique boundaries, and thus are more informative in functional roles[11]) were observed **(Fig. 1i, and Supplementary Fig. 5d)**. **(See Methods for details)**.

TAD boundaries with the greatest changes in frequency (from high to low frequency or reversely) were defined as variable TAD boundaries and were used in subsequent analysis. Compared to the small fraction of boundaries (1.01%, ~120 boundaries) that differed across ATs within the treatment groups, relatively few boundary changes were detected in each AT across treatment groups (average 0.18%, ~22 boundaries), which is consistent with previous findings of TAD stability across metabolic and physiological changes, and reflects a more fundamental organizational unit of chromosomes[12]. **(Fig. 2c, d)**

We found that some representative TAD boundaries shift between ATs embedded genes, including *EN1* and *TCF21* **(Fig. 2i, j)**. Mechanistically, the high frequency of the TAD boundary embedded with gene *EN1* in ULB leads to a reduction in TAD size (*i.e.*, some sub-TAD structures), thereby restricting more local interactions with the *EN1* promoter and enhanced transcription. The high-frequency boundary embedded with gene *TCF21* in ULB is the site of a disrupted former larger TAD structure in GOM, which was divided in two. Compared to the larger TAD structure in GOM, which allows further external interactions with *TCF21*, the smaller TAD in ULB attenuates local interactions, thus decreasing its transcriptional levels.

In addition to the TE distribution in TAD boundaries, we also evaluated TE age based on sequence divergence (*i.e.*, lower divergence sequence indicating younger TEs). Generally, SINEs in common boundaries across ATs had lower divergence rates than SINEs in AT-specific boundaries. Although the mechanistic roles of TEs involved in TAD formation during speciation and adaptive evolution are not fully understood, this phenomenon suggests the possible involvement of evolutionarily younger TEs during the formation of functional, stable TAD boundaries in mammals, and thus elimination of older TEs[13].

Supplementary Note 4 Dynamic CTCF- mediated loops in ATs

To investigate the rewiring of CTCF-mediated loops in ATs with weight changes, we compiled a genome-wide catalog of CTCF loops across four ATs under three nutritional conditions at a 5-kb resolution using the Fit-Hi-C package and identified ~6412 CTCF loops for each of 12 groups at a 5-kb resolution **(Supplementary Fig. 21 and Supplementary Data 4)**.

As expected, CTCF loop dynamics are more profound among ATs than among treatment groups **(Supplementary Fig. 21c, d)**. Genes residing within CTCF loops were more highly expressed than those outside loops **(Supplementary Fig. 21e)**. In detail, genes (averaged ~64) located in VAT-specific CTCF loops (compared with ULB for each group) (**Supplementary Fig. 22**) were primarily related to responses to inflammation and immunity (*e.g.*, 'positive regulation of inflammatory response' and 'T cell activation'). This indicates the intrinsically functional and metabolic differences among ATs. Genes located in the WG-specific CTCF loop (compared with the NC group across ATs) were also mainly associated with inflammation response (such as 'chemotaxis' and 'leukocyte differentiation') **(Supplementary Fig. 23)**, reflecting the increased inflammation in ATs with weight gain.

Notably, compared with CTCF motifs that do not participate in loops, the CTCF motifs of loop anchors are more evolutionally conserved, and the anchors of loops that contain genes are more conserved than those depleted with genes **(Supplementary Fig. 24)**. This indicates that these evolutionarily conserved motifs of CFCF loop anchors are of potential regulatory importance

(constraint the regulatory interactions within loops)[14,15] for ATs with weight changes.

Supplementary Note 5 Identification of evolutionary patterns of local spatial context in mammalian ATs

We categorized homologous regions (949.94 Mb, or 30.65% of the human genome) across seven mammals into 26 states with a different evolutionary spatial context in IS using the Phylo-HMGP model[16]. Our findings reflect three evolutionarily distinct patterns of local spatial context: conserved high IS (517.71 Mb, 54.55%), conserved low IS (297 Mb, 31.29%), and non-conserved IS (134.43 Mb, ~14.16%) **(Supplementary Fig. 34a, and Supplementary Data 7)**. Genes (3,304) located in conserved high IS regions mainly reflected core AT functions, such as 'energy and lipid metabolism,' while genes (633) located in non-conserved IS regions have dispersed functions, suggesting potential physiological discrepancies of ATs across species **(Supplementary Data 8)**.

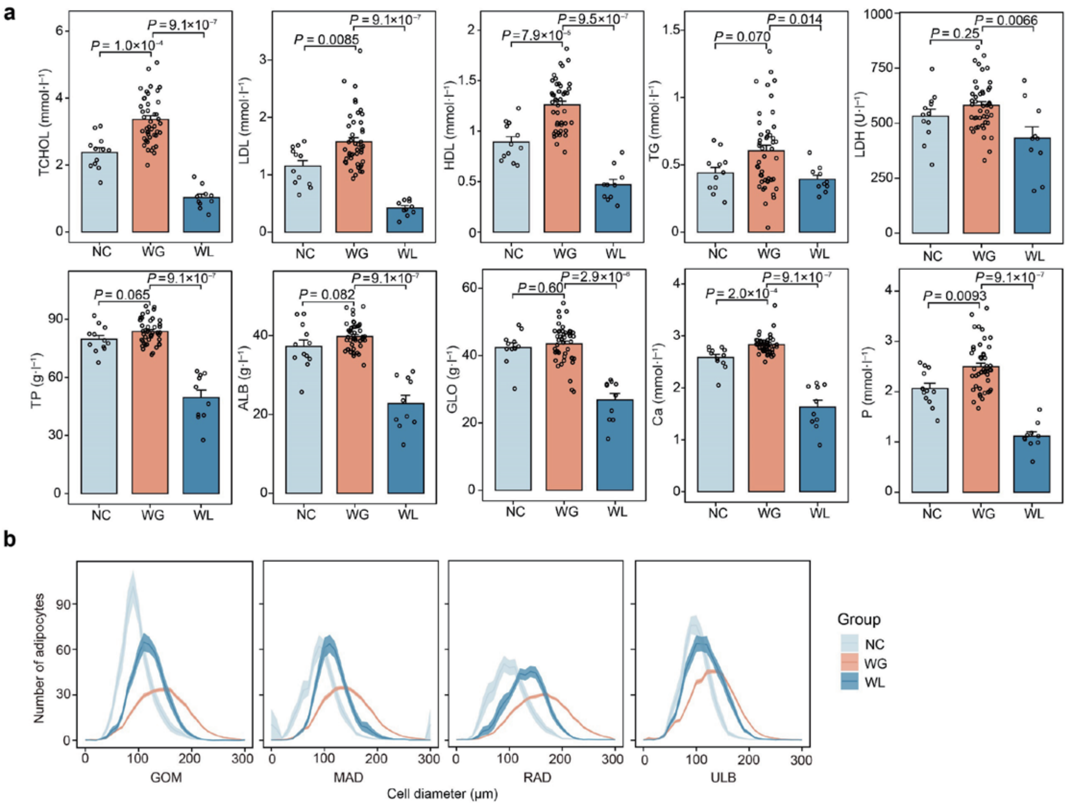

Supplementary Fig. 1. Overview of serum metabolic indicators and adipocyte size across body weight gain and loss in pigs.

a Difference of serum metabolic indicators across weight gain and loss, including total cholesterol (TCHOL), low-density lipoprotein (LDL), high-density lipoprotein (HDL), triglyceride (TG), lactate dehydrogenase (LDH), total protein (TP), albumin (ALB), globulin (GLO), calcium (Ca), and phosphorus (P). Data are presented as mean values ± SD (NC n = 12, WG n = 46, WL n = 10). Dots represent the values of each pig. Statistical significance was performed using two-sided Wilcoxon signed-rank test.

b Adipocyte size distribution of each AT during body weight gain and loss. Adipocyte size (diameter) is measured by histology. Colored lines represent mean values across replicates, and lighter-colored shading around the mean represents standard error. Despite an expected increase in adipocyte size after weight gain, the deviation of adipocyte size distribution also increased. This highlights the wide range of sizes, revealing the presence of both hypertrophy and hyperplasia for adipocytes as body weight increased.

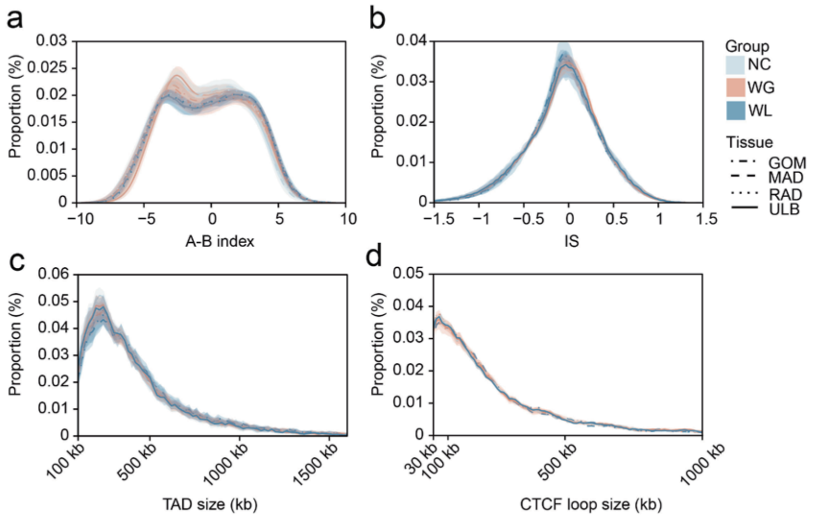

Supplementary Fig. 2. The distribution of A-B index **(a)**, IS **(b)**, TAD size (kb) **(c)**, and CTCF loop size (kb) **(d)** proportions across groups for a given tissue. Colored lines represent mean value across replicates and shading around the mean represents standard deviation across replicates.

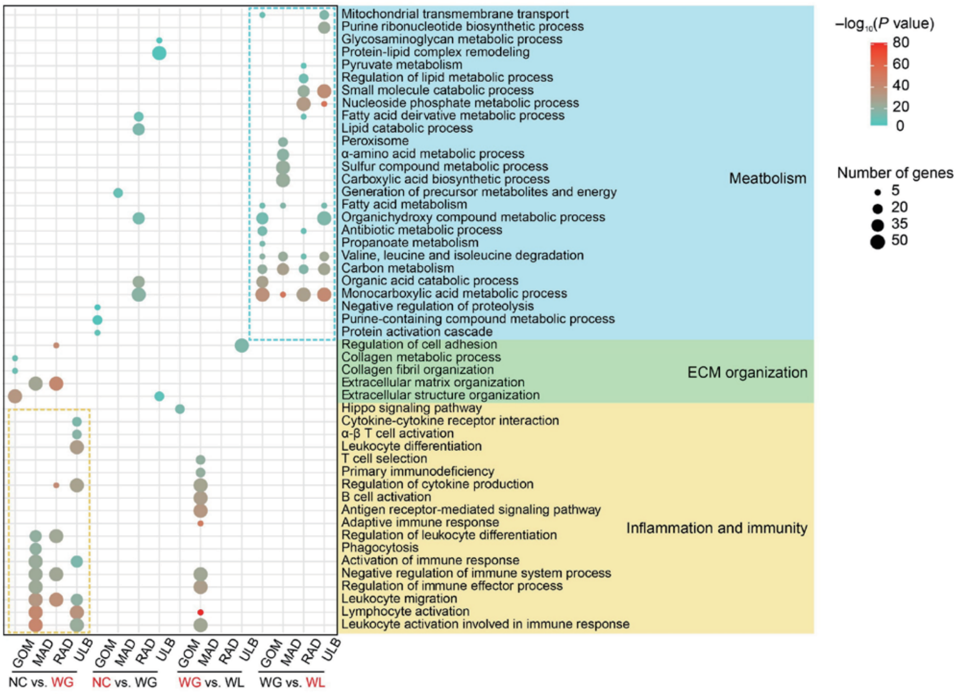

Supplementary Fig. 3. Functional enrichment for differentially expressed (DEs) PCGs between groups. Plot showing the top 10 enriched GO terms of DE PCGs in NC-WG and WG-WL comparisons for each AT. The names highlighted in red for each pairwise comparison represent the group in which the genes were highly expressed. The color shades on the Y-axes highlight the categories of GO terms, *i.e.,* metabolism-related terms (blue), extracellular matrix (ECM) organization-related terms (green), inflammation- and immunity-related terms (yellow), and others (n = 52, not shown in the plot). The size of the dot represents the number of enriched genes, and the dot color represents the -log$_{10}$(P-value) (unadjusted). P values were calculated based on a one-sided accumulative hypergeometric test.

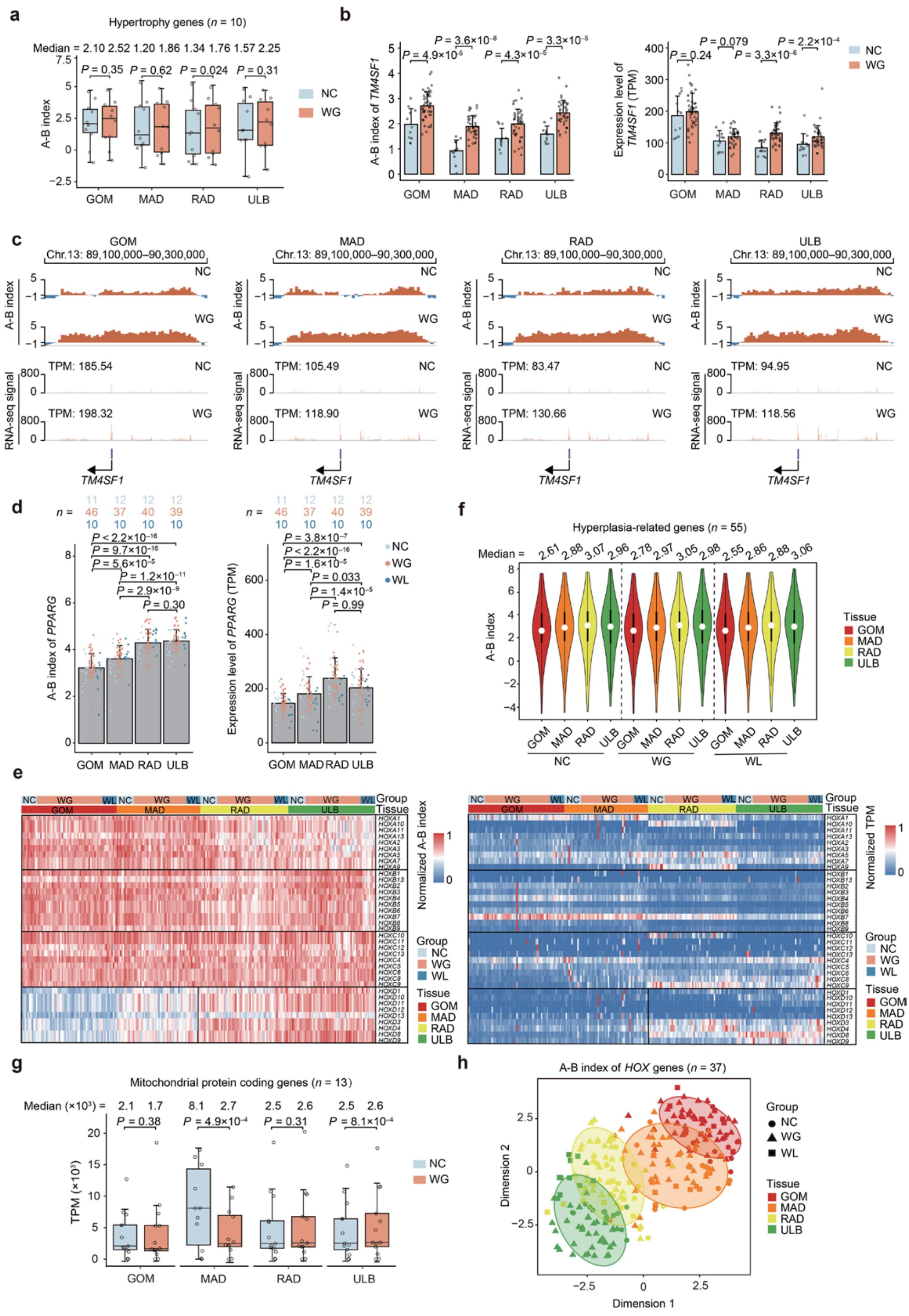

Supplementary Fig. 4. Pattern of compartment status and expression of genes suggesting characteristics of distinct ATs.

a Compartment status and expression pattern of hypertrophy genes (*n* = 10) in each adipose depot across weight gain and loss. In the boxplot, the internal line indicates the median, the box limits indicate the upper and lower quartiles and the whiskers extend to

1.5 IQR from the quartiles. Statistical significance was determined using a one-sided Wilcoxon rank-sum test. *P* value is shown above each box of each AT.

b Histogram showing the compartment status and transcription of representative hypertrophy gene (i.e., *TM4SF1*). The data are shown as mean with SD (NC *n*=12, WG *n*=46). Statistical significance was determined using a one-sided Wilcoxon rank-sum test.

c A representative gene (*TM4SF1*) with changes in A/B compartment status during weight gain for each adipose depot. Tracks show compartment status (top) and gene expression level (bottom).

d Histogram showing the compartment status and transcription of representative hyperplasia genes (i.e., *PPARG*). Data are presented as mean values ± SD (*n* number is listed above each bar). Statistical significance was determined using a one-sided Wilcoxon rank-sum test.

e Heatmap showing the pattern of (left panel) compartment status and (right panel) expression of *HOX* genes across adipose depots, in which *HOXD* clusters showed a remarkable difference in compartment status between SAT and VATs. Within each treatment, the compartment status of all nine *HOXD* cluster genes (typically, *HOXD4,* a key regulator in controlling the adipocyte development [i.e., pre-adipocyte differentiation] of SATs) are similar in ULB and RAD, which are more active than in GOM and MAD (for example, A-B scores in NC, ULB: 2.37, RAD: 2.14, GOM: 1.15, MAD: 1.69).

f Change of compartment status of 55 hyperplasia genes between adipose depots in each group. In the violin plot, the internal dot indicates the median, the box limits indicate the upper and lower quartiles and the whiskers extend to 1.5 IQR from the quartiles.

g Expression level of 13 mitochondrial protein-coding genes in each AT between NC and WG group. In the boxplot, the internal line indicates the median, the box limits indicate the upper and lower quartiles and the whiskers extend to 1.5 IQR from the quartiles. Statistical significance was determined using a one-sided Wilcoxon rank-sum test.

h t-SNE plots based on the compartment status of 37 *HOX* genes. The ellipses indicate the samples of each AT with similar profiles, constructed at a probability of 0.85.

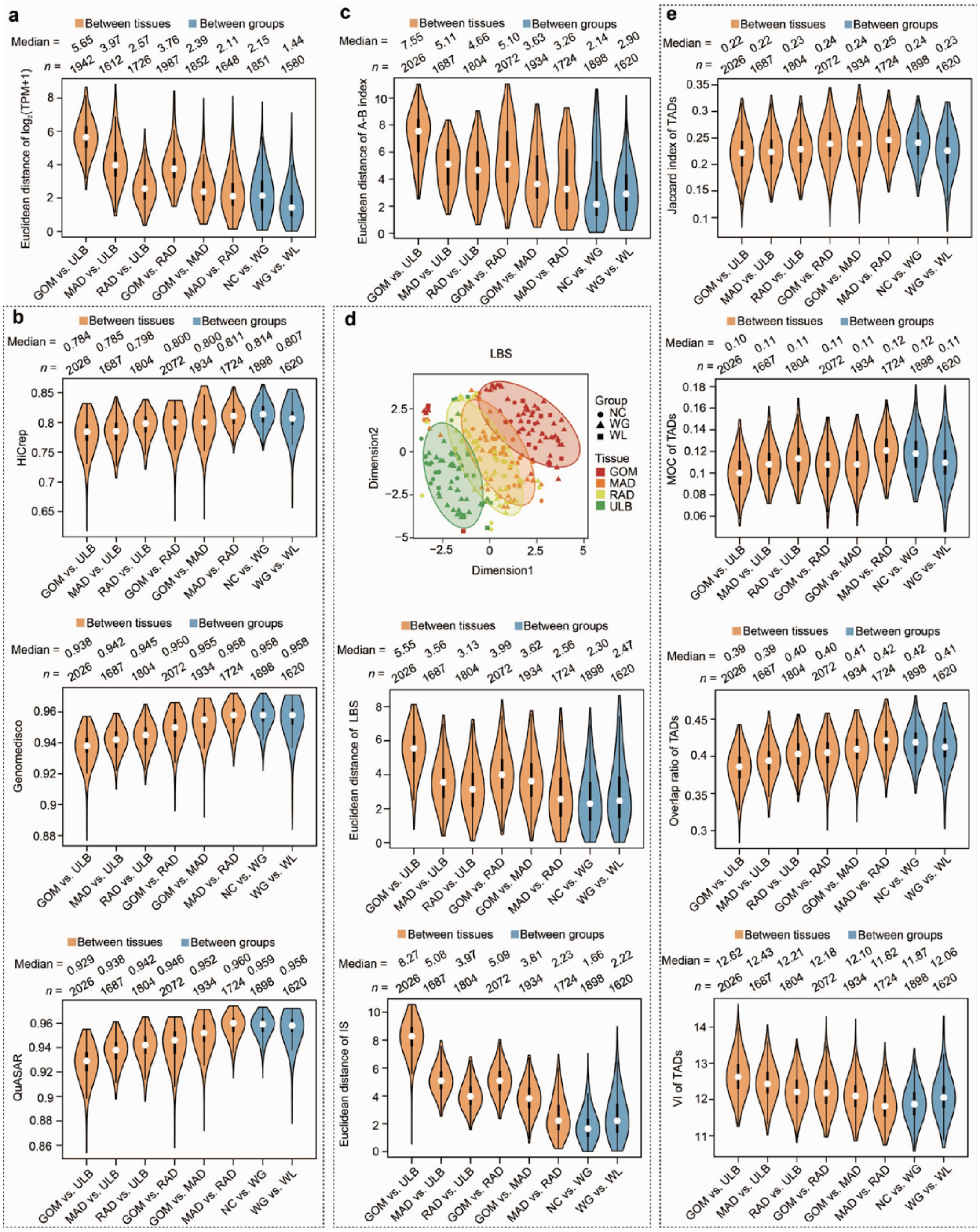

Supplementary Fig. 5. Comparison of variation in chromatin architecture between adipose depots and between groups.

a Violin plot shows the distance of PCGs expression between groups (blue) and ATs (yellow), which is derived from the t-SNE plot (**Fig. 1g**). In the violin plot, the internal dot indicates the median, the box limits indicate the upper and lower quartiles and the whiskers extend to 1.5 IQR from the quartiles.

b Comparison of variation in global genome architecture between groups (blue) and ATs (yellow) using HiCrep SCC (stratum-adjusted correlation coefficient), Genomedisco, and QuASAR based on e Knight-Ruiz and BNBC (see Methods section '*Initial processing of*

Hi-C data'). For example, the violin plot shows the HiCrep SCC (at 100-kb) between samples, which are more distinguishable among distinct ATs (average SCC = 0.7963; for a SAT vs. three VATs, SCC = 0.7951) than between groups (average SCC = 0.8114). In the violin plot, the internal dot indicates the median, the box limits indicate the upper and lower quartiles and the whiskers extend to 1.5 IQR from the quartiles.

c Violin plot showing the distance from AB compartments between groups (blue) and ATs (yellow), which was derived from the t-SNE plot (see **Fig. 1g**). In the violin plot, the internal dot indicates the median, the box limits indicate the upper and lower quartiles and the whiskers extend to 1.5 IQR from the quartiles.

d (top) t-SNE clustering of samples using local boundary score (LBS). For the t-SNE plot, ellipses indicate AT samples with similar profiles, constructed at a probability of 0.85. Violin plot shows the distance of LBS (middle) and IS (derived from **Fig. 1i**) (bottom) between samples, respectively. In the violin plot, the internal dot indicates the median, the box limits indicate the upper and lower quartiles and the whiskers extend to 1.5 IQR from the quartiles.

e Violin plot showing global relationships of TAD organization between groups (blue) and ATs (yellow), as reflected by the Jaccard index, MOC overlap ratio, overlap ratio, and VIs. See **Method** for details. In the violin plot, the internal dot indicates the median, the box limits indicate the upper and lower quartiles and the whiskers extend to 1.5 IQR from the quartiles.

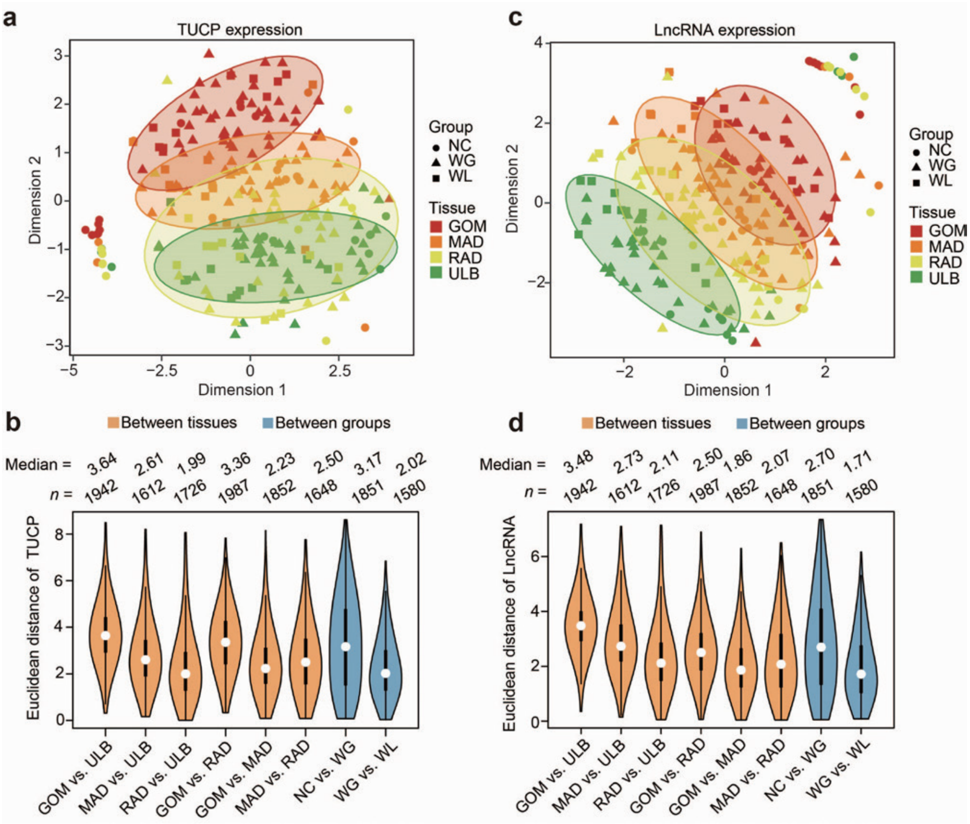

Supplementary Fig. 6. Comparison of transcriptional variation (lncRNA and TUCP) between ATs and between groups. (a, c) t-SNE clustering of samples using TUCP (**a**) and lncRNA (**c**) expression, respectively. For the t-SNE plot, ellipses indicate AT samples with similar profiles, constructed at a probability of 0.85. **(b, d)** Violin plot showing the distance of TUCP (**b**) and lncRNA expression (**d**), which was derived from the t-SNE plot, between samples. In the violin plot, the internal dot indicates the median, the box limits indicate the upper and lower quartiles and the whiskers extend to 1.5 IQR from the quartiles.

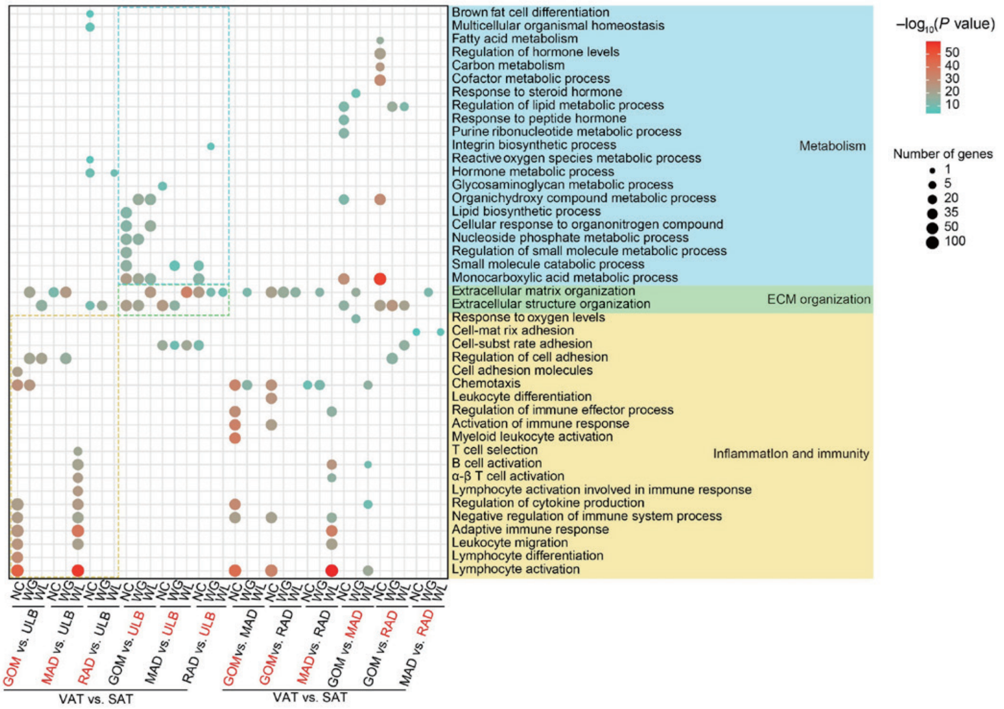

Supplementary Fig. 7. Functional enrichment for differentially expressed (DEs) PCGs between ATs. Plot showing the top 10 enriched GO terms of DE PCGs in pairwise AT comparisons. Pairwise comparisons listed at the bottom are sorted as follows: between VAT and SAT, within VATs. The names highlighted in red for each pairwise comparison represent the tissue in which the genes were highly expressed. The color shades on the Y-axes highlight the categories of GO terms, *i.e.,* metabolism-related terms (blue), extracellular matrix (ECM) organization-related terms (green), inflammation- and immunity-related terms (yellow), and others (*n* = 63, not shown in the plot). The size of the dot represents the number of enriched genes, and the dot color represents the -$\log_{10}$(*P*-value) (unadjusted). *P* values were calculated based on a one-sided accumulative hypergeometric test.

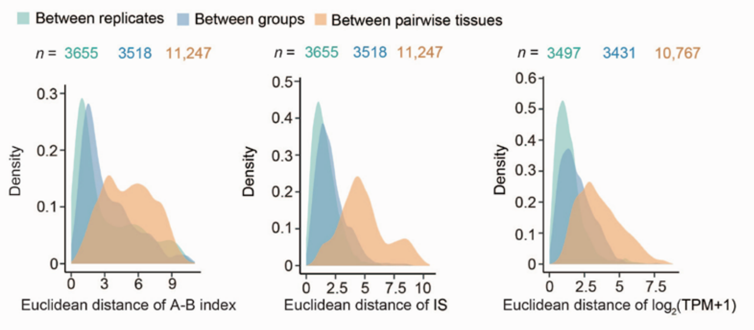

Supplementary Fig. 8. Correlation of gene expression, AB compartmentalization, and local spatial context (IS value) for pairs of samples between replicates, between tissues, and between groups. Statistical significance (*P* values) was determined using one-sided Wilcoxon rank-sum test. A-B index: between replicates vs. between groups, $P < 2.2\times10^{-16}$; between replicates vs. between ATs, $P < 2.2\times10^{-16}$; IS value: between replicates vs. between groups, $P < 2.2\times10^{-16}$; between replicates vs. between ATs, $P < 2.2\times10^{-16}$; Expression: between replicates vs. between groups, $P = 1.96\times10^{-9}$; between replicates vs. between ATs, $P < 2.2\times10^{-16}$.

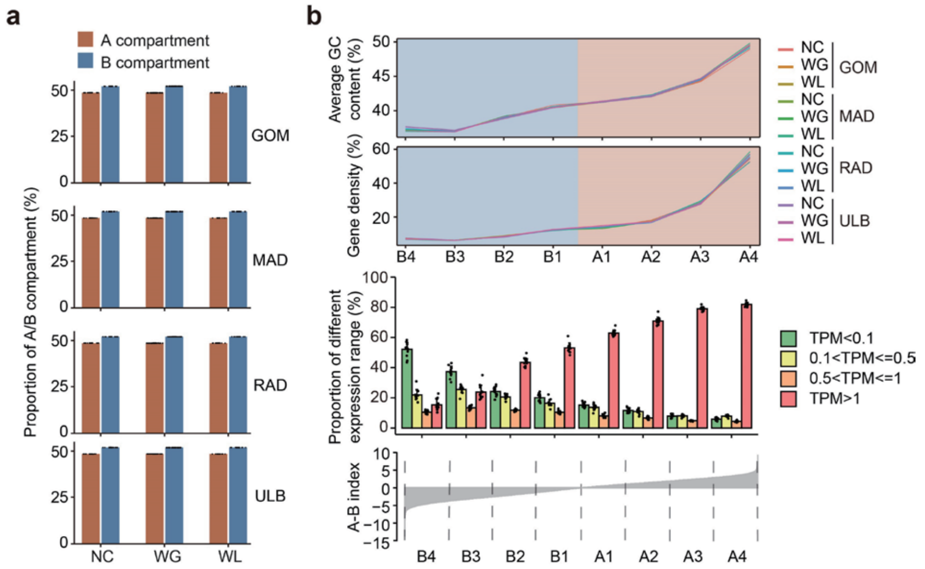

Supplementary Fig. 9. Fundamental features of compartments A/B identified in pig AT genomes.

a Length proportions of compartments A/B detected in genomes of each AT across three groups. Spots represent all replicates (NC *n* = 12, WG *n* = 46, WL *n* = 10). Based on Hi-C contact matrices at a 20-kb resolution, 48.13-48.34% of the genome (1090.60-1095.32 Mb) could be recognized as accessible A compartments, showing transcriptional activity, while the remaining genome was categorized as less accessible B compartments (51.66-51.87% of the genome or 1170.64-1175.36 Mb transcription suppressed).

b According to the A-B index, compartments A (A1–A4) and B (B1–B4) were divided into four equal parts. GC contents (top panel), gene densities (middle panel), and percentages of genes with different expression levels (bottom panel) were calculated. Data are presented as mean ± SD. Lines represent the means of GC content or gene density in each AT. The spots in histogram denote replicates (NC *n* = 12, WG *n* = 46, WL *n* = 10).

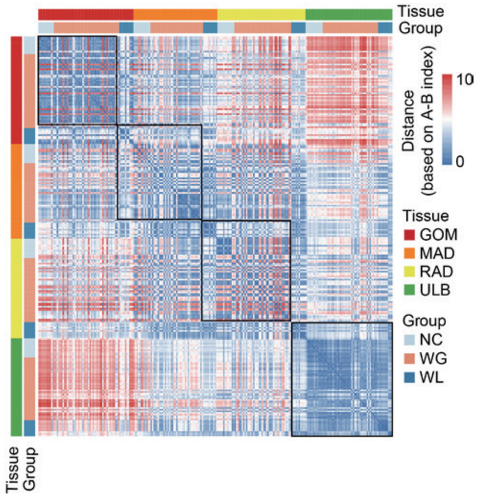

Supplementary Fig. 10. Estimation of the similarities among A/B compartments of distinct ATs across groups/treatments. Heatmaps indicating the distance of A/B compartment status between pairwise samples derived from the t-SNE plot using global A/B compartment (at a 100-kb resolution) as shown in **Fig. 1h**.

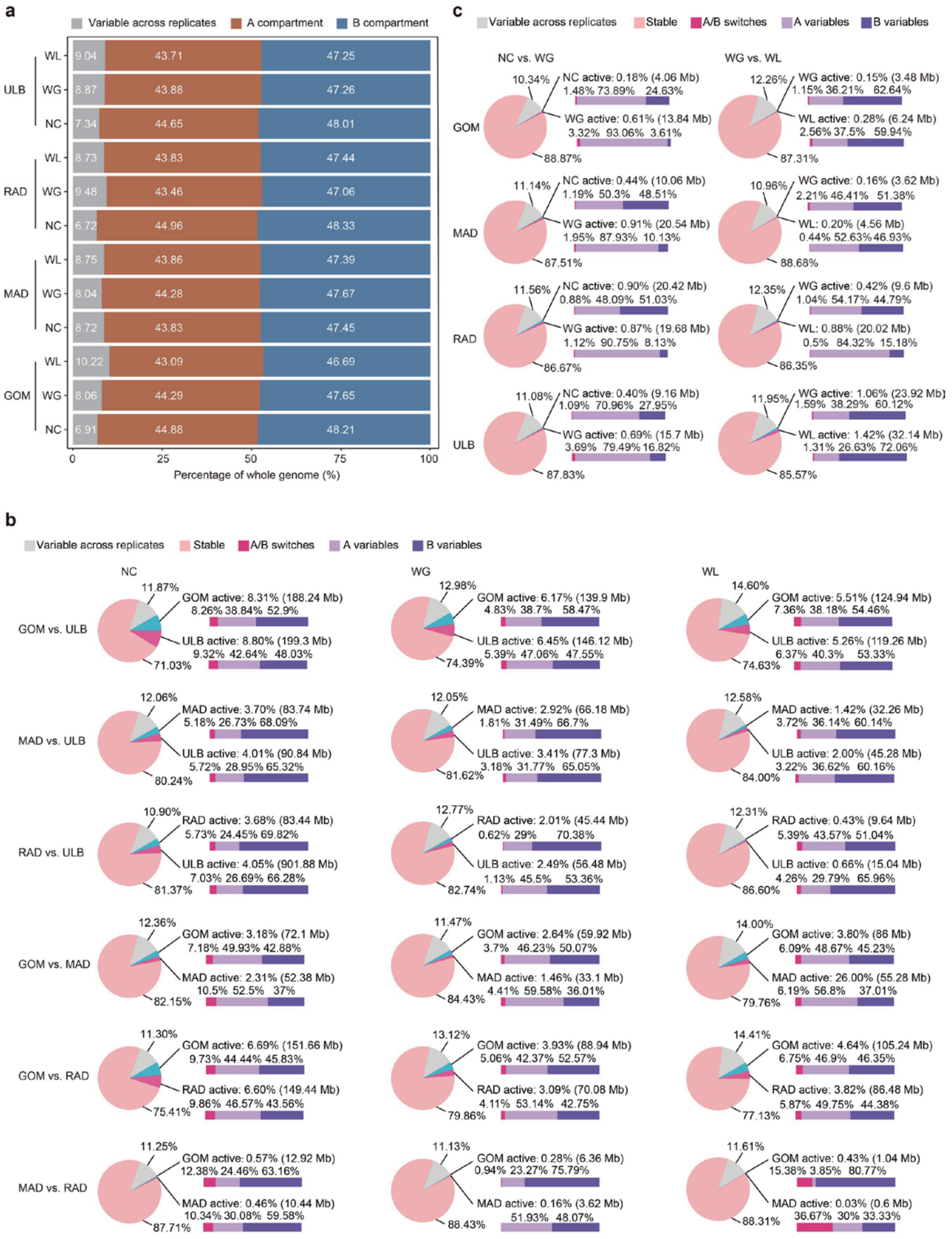

Supplementary Fig. 11. Fraction of genomic regions shows compartment arrangement between pairwise ATs within a group or between groups within each AT.

a Distribution of compartment A/B status across the genome at a 20-kb resolution for each ATs. Most (~91.6%) genomic regions showed the same states among over 80% of biological replicates.

b, c The pie chart shows AT-restricted (**b**) or group-restricted (**c**) active compartment status. The bar plot shows detailed compartment arrangement, including both A/B switches

(switched from A to B compartment status, or reversely) and A/B variables (having the significantly higher A-B index against their respective counterpart [Δ A-B index > 0.75, two-sided Student's *t*-test, FDR < 0.01]). Compared to compartment A/B switches, we observed more compartment A/B variables. We also observed 147.83 Mb (~6.52%) regions showing A/B compartment variables between pairwise AT comparisons, while only 9.44 Mb (~0.42%) showed A/B compartment switches. We observed 27.13 Mb (~1.20%) regions showing A/B compartment variables between groups within each AT, while only 0.43 Mb (~0.02%) showed A/B compartment switches.

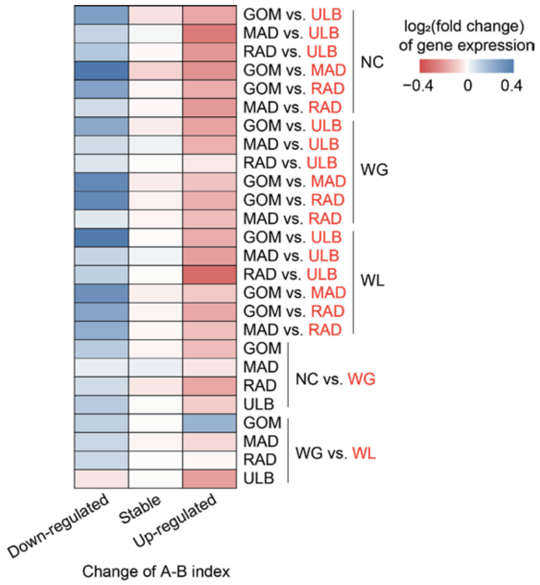

Supplementary Fig. 12. Distribution of fold-change in gene expression for genes with AT-restricted or group-restricted active compartment status.

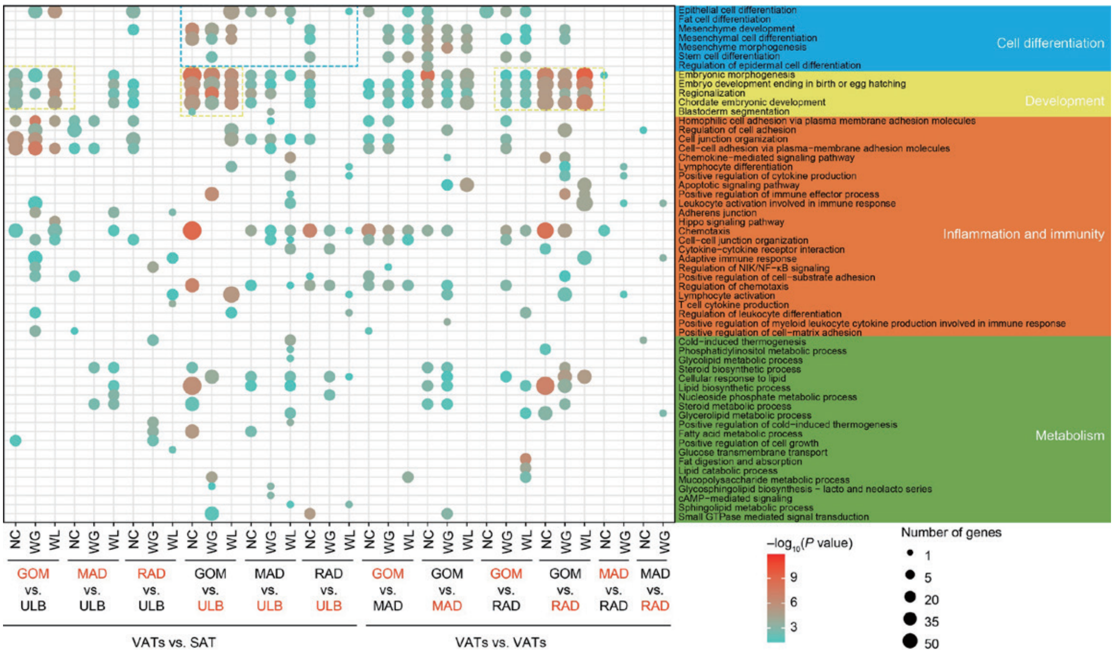

Supplementary Fig. 13. Functional enrichment for genes with changes in compartment status between ATs. Plot showing the top 20 enriched GO terms of genes with compartment status differences (including A/B switches and A/B variables) in pairwise AT comparisons. Pairwise comparisons listed at the bottom are sorted as follows: between VAT and SAT, within VATs. The names highlighted in red for each pairwise comparison represent the tissue in which the genes were active. The color shades on the Y-axes highlight the categories of GO terms, *i.e.,* cell differentiation (blue), development (yellow), inflammation- and immunity-related terms (orange), metabolism-related terms (green), and others (n = 331, not shown in the plot). The size of the dot represents the number of enriched genes, and the dot color represents the -$\log_{10}$(P-value) (unadjusted). P values were calculated based on a one-sided accumulative hypergeometric test.

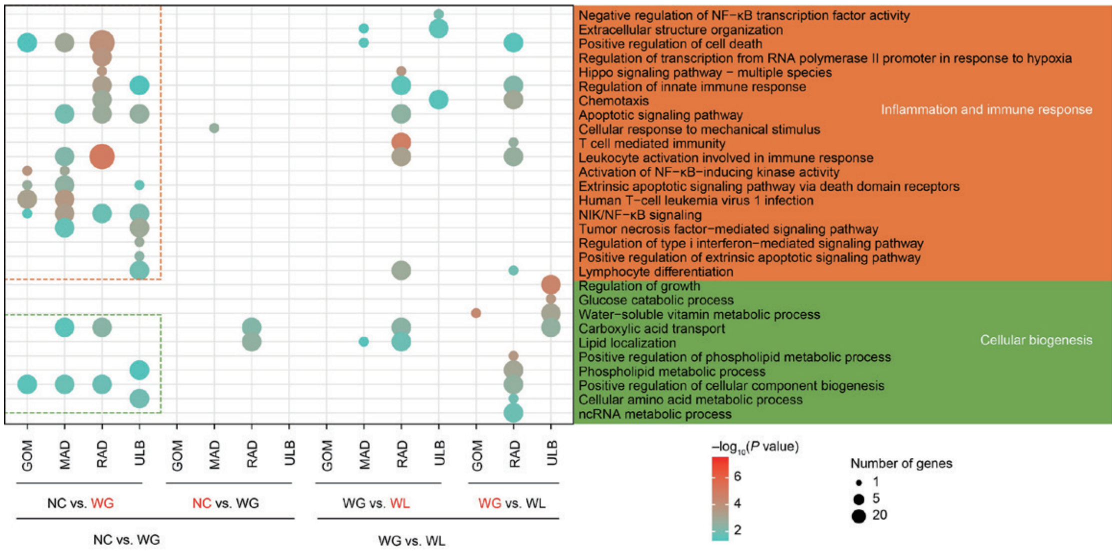

Supplementary Fig. 14. Functional enrichment for genes with changes in compartment status between groups. Plot showing the top 20 enriched GO terms of genes with compartment status differences (including A/B switches and A/B variables) in NC-WG and WG-WL comparisons for each AT. The names highlighted in red for each pairwise comparison represent the group in which the genes were active. The color shades on the Y-axes highlight the categories of GO terms, *i.e.,* inflammation and immunity-related terms (orange), metabolism-related terms (green), and others (n = 157, not shown in the plot). The size of the dots represents the number of enriched genes, and the dot color represents the -$\log_{10}$(P-value) (unadjusted). P values were calculated based on a one-sided accumulative hypergeometric test.

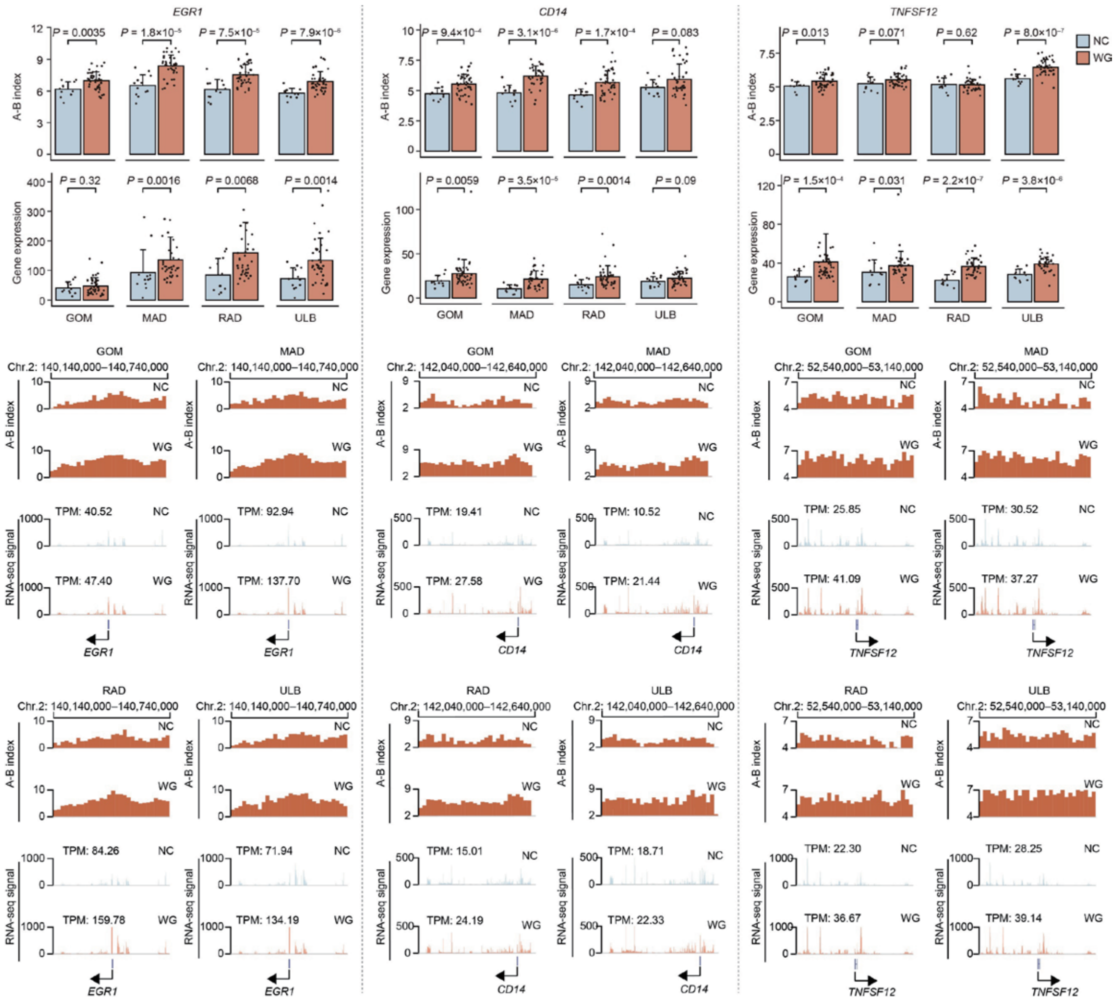

Supplementary Fig. 15. Histograms of compartment status and transcription level of representative hypoxia genes (*i.e.*, *EGR1*), as well as representative inflammation genes (i.e., *CD14* and *TNFSF12*) in each AT after weight gain. The data show means with SD (NC *n* = 12, WG *n* = 46). Statistical significance was determined using a one-sided Wilcoxon rank-sum test. Tracks show compartment status (top) and gene expression level (bottom).

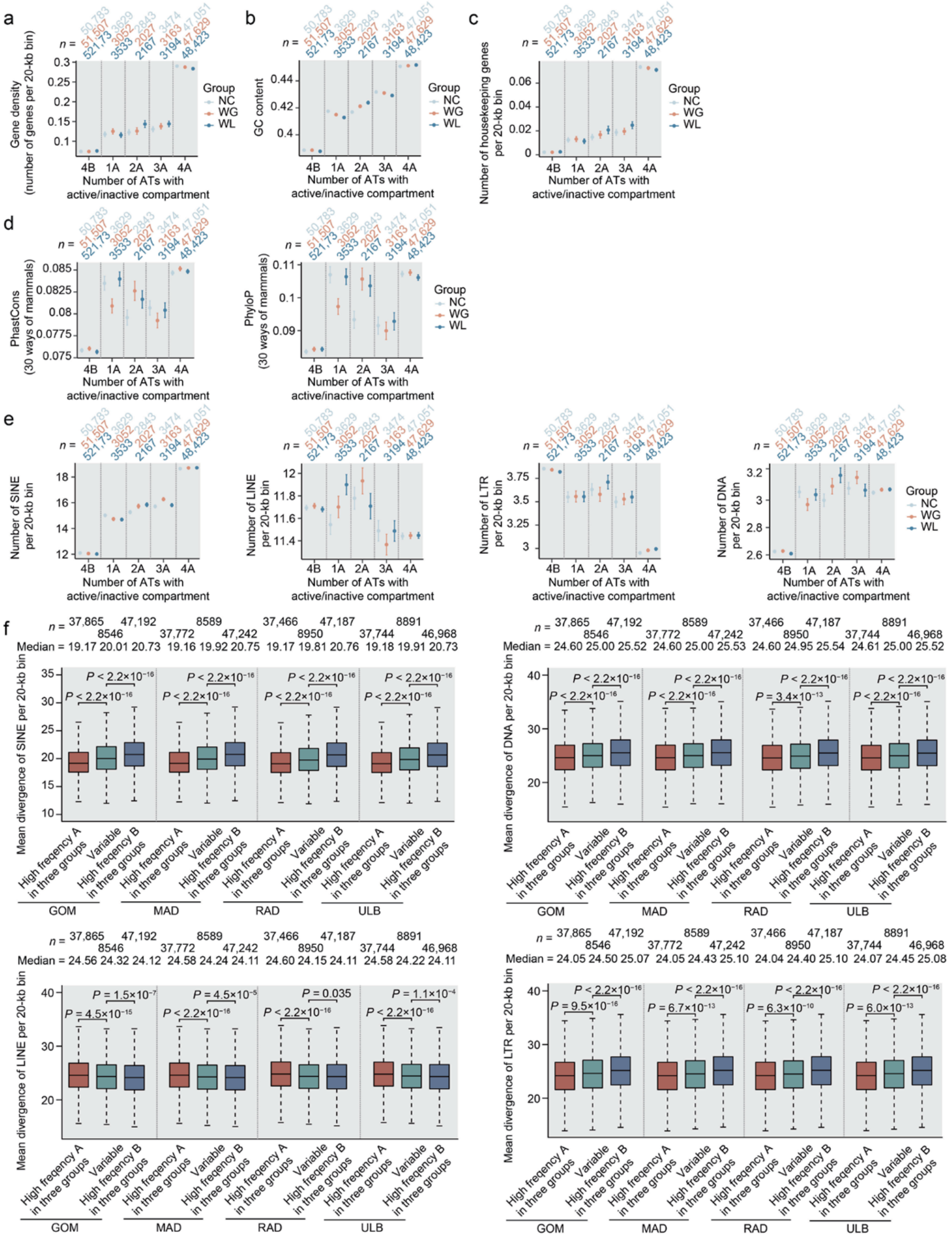

Supplementary Fig. 16. Genome regions with stable active compartment status across Ats are enriched for functional elements. (a–f) Across compartment stability types (different number of Ats being observed with a high frequency of active or inactive compartment). Gene density (**a**), GC content (**b**), housekeeping genes (**c**), evolutionary conservation (inferred by phastcons and phyloP value) (**d**), and four types of transposon elements (TEs) (including SINE, LINE, LTR, and DNA elements) (**e**) at each type of compartment stability are shown. Data are presented as mean ± SD. **(f)** Boxplot showed

an average divergence of TEs (i.e., SINE, LINE, LTR, and DNA, respectively) at regions (20-kb bins) with different stability of active compartment status. In the boxplot, the internal line indicates the median, the box limits indicate the upper and lower quartiles and the whiskers extend to 1.5 IQR from the quartiles. *P* values determined by one-sided Wilcoxon rank-sum test.

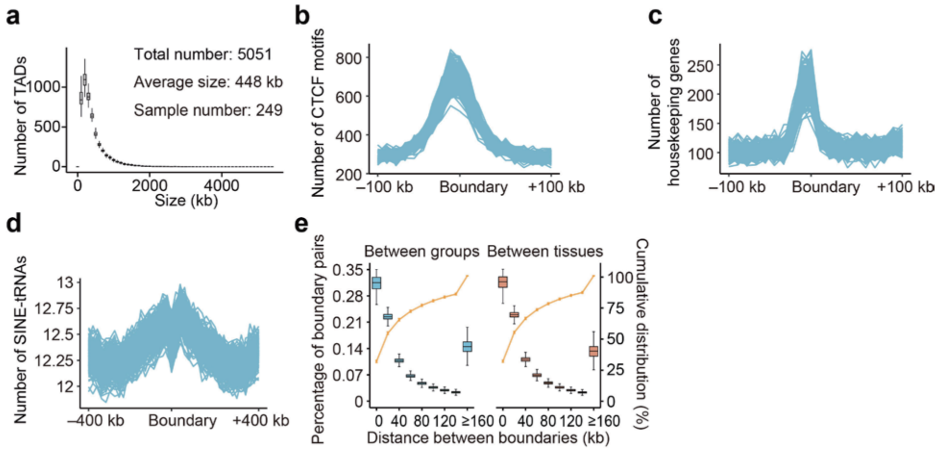

Supplementary Fig. 17. Characteristics of TADs and dynamic pattern across ATs response to weight changes.

a Boxplot of the distribution of sizes of TADs for four distinct ATs at three groups, which were called by deDoc method. The average number of TADs and average sizes were denoted (n = 249). In the boxplot, the internal line indicates the median, the box limits indicate the upper and lower quartiles and the whiskers extend to 1.5 IQR from the quartiles.

b–d Enriched with CTCF motifs (**b**), housekeeping genes (**c**), and typical TEs (i.e., SINE-tRNA) (**d**) at TAD boundary regions.

e Proportion of proximity boundaries between ATs and between groups. Percentage of proximity TAD boundaries between pairwise samples was calculated. Boxplot indicates the percentage of TAD boundaries with different genomic proximities. In the boxplot, the internal line indicates the median, the box limits indicate the upper and lower quartiles and the whiskers extend to 1.5 IQR from the quartiles. Between groups: n = 3518 for each bar, between tissues: n = 11,247 for each bar. The cumulative distribution was also plotted. ~80.08% (4235 of 5289) of boundaries are colocalized within 100 kb in a given AT across conditions, and ~81.28% (4170 of 5131) are colocalized in distinct ATs under the same condition.

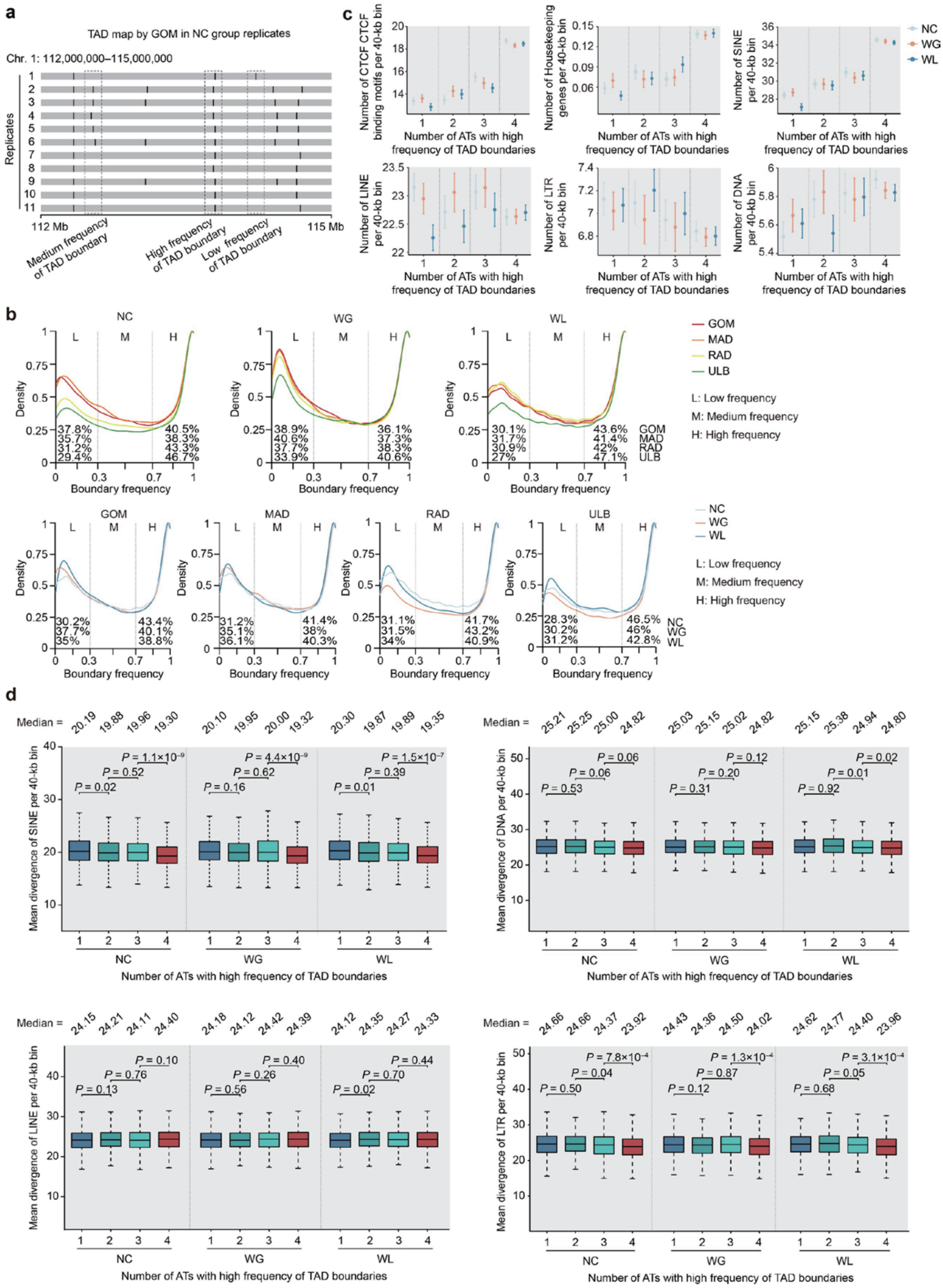

Supplementary Fig. 18. Aggregated TAD boundary enriched regions and distribution of TAD boundary frequency located in these regions.

a Example TAD maps from replicates (rows) of GOM in the NC group. A 3 Mb window from pig chromosome 1 (suscrofa 11.1) indicates a schematic of the TAD boundary frequency calculation. Each grey line represents the genomic extent of a TAD. The black dashed box (200 kb regions) outlines examples of aggregated TAD boundary enriched regions

(harboring bookended TAD boundaries [black vertical lines]), with high (>70%), low (<30%), and medium (30<70%) frequency, respectively.

b Frequency distribution of TAD boundary across different ATs within the same group or across groups of a given AT. The vertical dashed lines indicate low (L, <0.3, left) and high frequencies (H, >0.7, right); otherwise, the medium frequency is represented (M, >0.3 and <0.7). The cumulative fraction of low- and high-frequency boundaries is indicated below, in the following order: GOM, MAD, RAD, and ULB. Nearly half of the boundaries showed high frequency across biological replicates, indicating boundary stability.

c Stable TAD boundaries across ATs are enriched for functional elements. Across TAD boundary stability types (different numbers of tissues being observed with a high frequency of TAD boundary), CTCF binding motifs, housekeeping genes, and four types of TEs (SINE, LINE, LTR, and DNA element) at boundaries are shown. Data are presented as mean ± SD. 1: NC *n* = 1171, WG *n* = 963, WL *n* = 1185; 2: NC *n* = 811, WG *n* = 557, WL *n* = 822; 3: NC *n* = 806, WG *n* = 606, WL *n* = 802; 4: NC *n* = 4175, WG *n* = 4844, WL *n* = 4447.

d Boxplot of average divergence of TEs (i.e., SINE, LINE, LTR, and DNA, respectively) at boundaries across boundaries with different stabilities. In the boxplot, the internal line indicates the median, the box limits indicate the upper and lower quartiles and the whiskers extend to 1.5 IQR from the quartiles. NC: 1 *n* = 1171, 2 *n* = 810, 3 *n* = 806, 4 *n* = 4166; WG: 1 *n* = 962, 2 *n* = 557, 3 *n* = 606, 4 *n* = 4834; WL: 1 *n* = 1184, 2 *n* = 821, 3 *n* = 801, 4 *n* = 4439. *P* values determined by one-sided Wilcoxon rank-sum test.

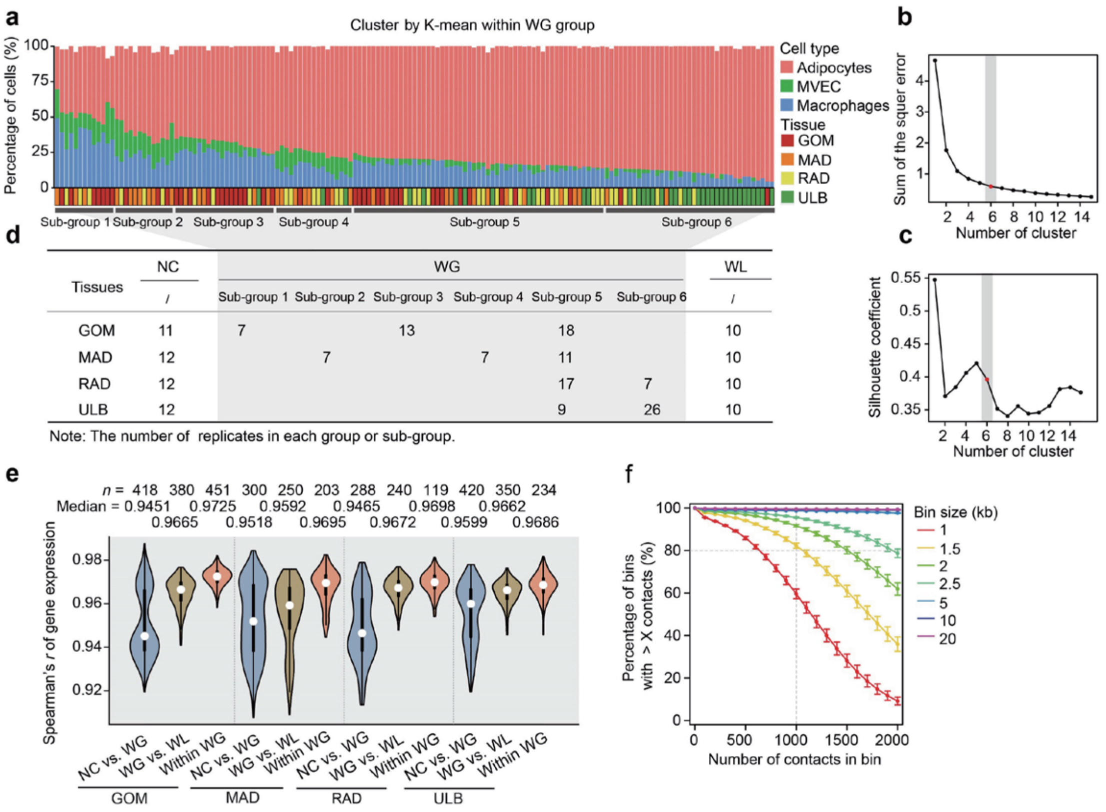

Supplementary Fig. 19. Transcriptomic similarities in WG group for each AT.

a–d Defining the sub-groups within the WG group. For the replicates (n = 46) in WG groups for each AT, we grouped replicates with high similarities in cell composition (including adipocyte, macrophages, and microvascular endothelial cells [MVEC]) based on the K-mean cluster of all the 157 samples, yielded WG sub-groups for each AT. All samples were classified into 6 clusters based on the similarity of cell composition. (**a**) The optimal number of clusters was assessed based on the relatively lower distance (sum of the squares) within the cluster (**b**) and a higher silhouette coefficient. (**c**) Therefore, we empirically defined 2-3 sub-groups for a given AT in the WG group (7-26 biological replicates per sub-group) (as shown in the table) based on the transcriptomic similarity shaped by estimated compositions of three typical cell types in ATs. The number of replicates is indicated for each subset. (**d**)

e Boxplot showing Spearman's correlation of gene expression between samples within WG sub-groups, between WG sub-groups and NC, and between WG sub-groups and WL. In the boxplot, the internal line indicates the median, the box limits indicate the upper and lower quartiles and the whiskers extend to 1.5 IQR from the quartiles. We observed higher transcriptomic similarity within WG sub-groups than between different groups, which indicated suitable representativeness.

f Resolution of the merged intra-chromosomal Hi-C contact. We merged the effective reads from all biological replicates for each given AT in the NC and WL groups, and the defined 2-3 sub-groups for a given AT in the WG group (as shown in **a**) reached a maximum resolution of 1.5 kb. Data are presented as mean ± SD (n = 18). An average of over 98.66%

of loci at 5 kb size have at least 1,000 contacts in each adipose, allowing us to pinpoint enhancers interacting with promoters at a high resolution.

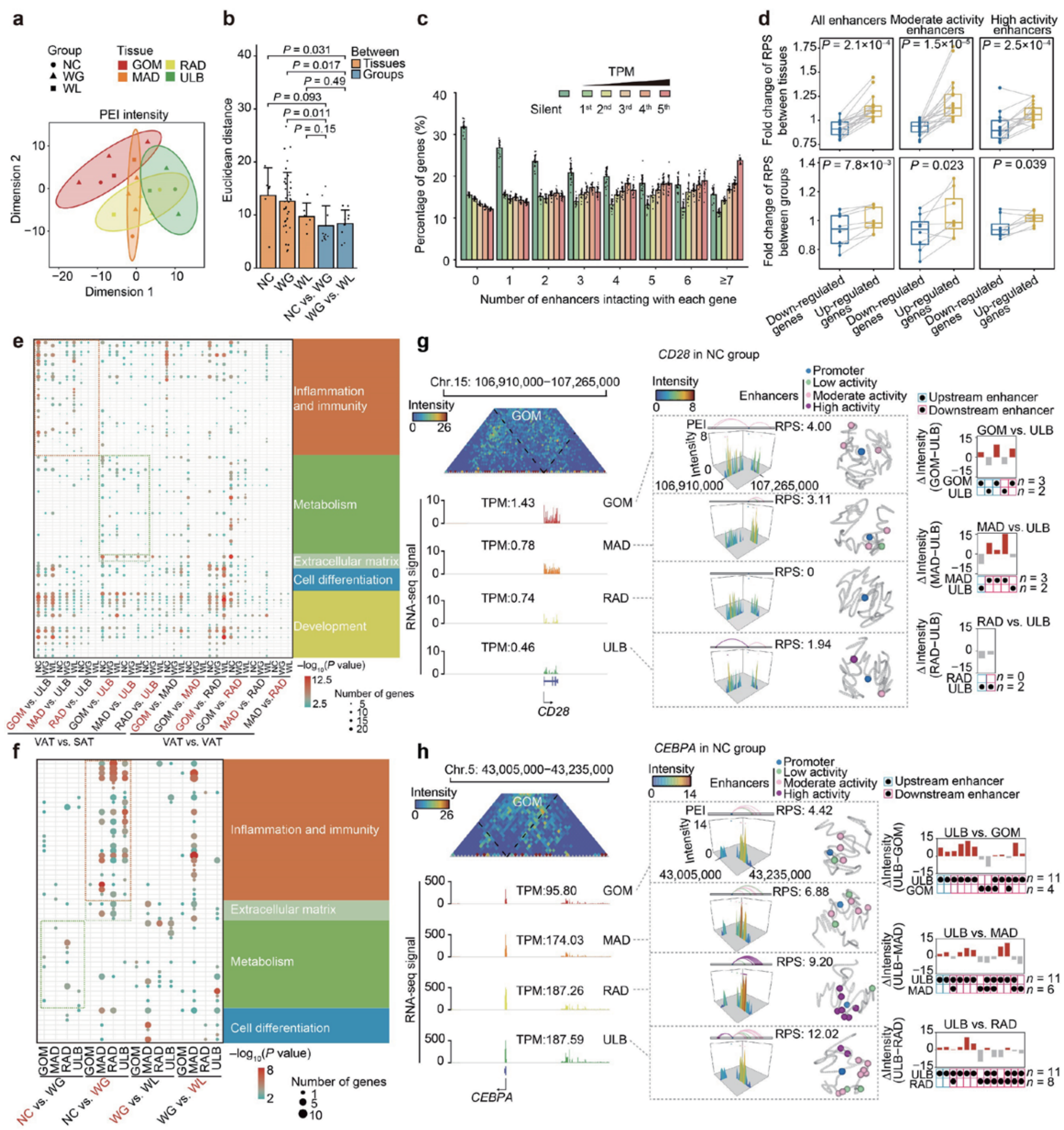

Supplementary Fig. 20. Transcriptional regulation through chromatin rewiring of PEIs in different ATs across groups.

a t-SNE plot of the intensity of promoter and enhancer interactions (PEI).

b Histogram of the distance between PEI interaction intensities between samples from the t-SNE plot in **a.** Data are presented as mean ± SD. Between tissues: NC *n* = 6, WG *n* = 37, WL *n* = 6; Between groups: NC vs. WG *n* = 10, WG vs. WL *n* = 10. *P* values determined by two-sided Wilcoxon rank-sum test.

c Proportions of gene promoters from each expression category interacting with zero to more than six enhancers. This result suggests that enhancers could provide an additive effect on target gene transcription levels. Data are presented as mean ± SD (*n* = 18).

d Genes with differential expression changes showed accordant RPS changes between ATs and between groups. Results are shown for genes with all enhancers, moderate-activity enhancers, and high-activity enhancers, respectively. Genes with up-regulated expression levels often showed larger RPS (as shown for each pairwise ATs in each group).

Statistical significance was calculated by one-sided paired-samples Wilcoxon rank-sum test (n.s., $P \geq 0.05$; *, $P < 0.05$; **, $P < 0.01$, ***, $P < 0.001$). In the boxplot, the internal line indicates the median, the box limits indicate the upper and lower quartiles and the whiskers extend to 1.5 IQR from the quartiles. Between tissues: n = 18; Between groups: n = 8. While this trend was not significant in some comparisons (especially between groups), a potential explanation is that individual genes are expressed to varying degrees through a subset of regulatory elements (e.g., enhancer) and could be better described as fine-tuning rather than independently affecting (inducing or silencing) transcription. These multiple regulatory interactions can also exert synergistic or non-linear effects on gene regulation.

e Functional enrichment for genes with co-variation between RPS and gene expression between ATs. Plot showing the top 20 enriched GO terms of genes with RPS and expression differences in pairwise AT comparisons. Pairwise comparisons listed at the bottom are sorted as follows: between VATs and SATs, within VATs. The names highlighted in red for each pairwise comparison represent the tissue in which genes have higher RPS values. The color shades on the Y-axes highlight the categories of GO terms, *i.e.,* inflammation and immunity-related terms (orange), metabolism-related terms (green), ECM-related terms (light green), cell differentiation (blue), development (yellow), and others (n = 218, not shown in the plot). The size of the dot represents the number of enriched genes, and the dot color represents the $-\log_{10}(P\text{-value})$ (unadjusted). P values were calculated based on a one-sided accumulative hypergeometric test.

f Functional enrichment for genes with co-variation between RPS and gene expression across groups. Plot showing the top 20 enriched GO terms of genes with RPS and expression changes in the NC-WG and WG-WL comparisons for each AT. The names highlighted in red for each pairwise comparison represent the group in which genes have higher RPS values. The color shades on the Y-axes highlight the categories of GO terms, *i.e.,* inflammation and immunity-related terms (orange), ECM-related terms (light green), metabolism-related terms (green), cell differentiation (blue), and others (n = 109, not shown in the plot). The size of the dot represents the number of enriched genes, and the dot color represents the $-\log_{10}(P\text{-value})$ (unadjusted). P values were calculated based on a one-sided accumulative hypergeometric test.

g Schematic representation of PEIs for a typical pro-inflammation gene (*CD28*) that are highly abundant in VATs in the NC group. (left) Promoter-centered interactions and expression levels for gene examples across four ATs. (middle left) Interaction metaplots of promoter-centered regions across four ATs. (middle right) 3D structural models of corresponding genomic regions. (right) Differences in PEI intensity between pairwise AT comparisons.

h Schematic representation of PEIs for typical adipogenesis gene (*CEBPA*) that are highly abundant in SAT in NC group.

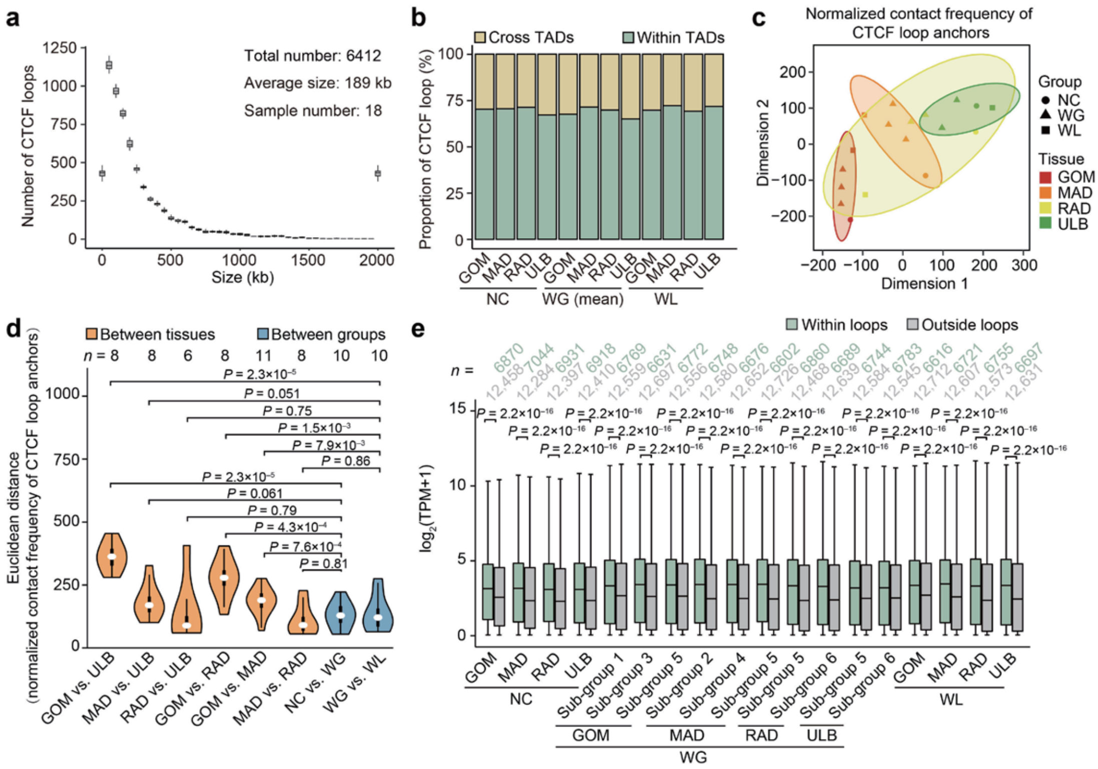

Supplementary Fig. 21. Characteristics of CTCF-mediated loops and dynamic pattern across ATs response to weight changes.

a Distribution of sizes of CTCF loops for four distinct ATs at three groups, analyzed by the Fit-Hi-C package. In the boxplot, the internal line indicates the median, the box limits indicate the upper and lower quartiles and the whiskers extend to 1.5 IQR from the quartiles (n = 18). The average number of CTCF loops and average sizes are denoted.

b Proportions of CTCF loops within or cross TAD.

c t-SNE plots based on chromatin interactions of merged CTCF loop anchors across all samples.

d Violin plot shows the distance between samples derived from the t-SNE plot (**c**). In the violin plot, the internal dot indicates the median, the box limits indicate the upper and lower quartiles and the whiskers extend to 1.5 IQR from the quartiles. P values determined by one-sided Wilcoxon rank-sum test.

e Expression level of genes located within or outside CTCF loops. In the boxplot, the internal line indicates the median, the box limits indicate the upper and lower quartiles and the whiskers extend to 1.5 IQR from the quartiles. The numerical value above each bar indicates the number (n) of genes. Statistical significance was determined using a two-sided Wilcoxon rank-sum test.

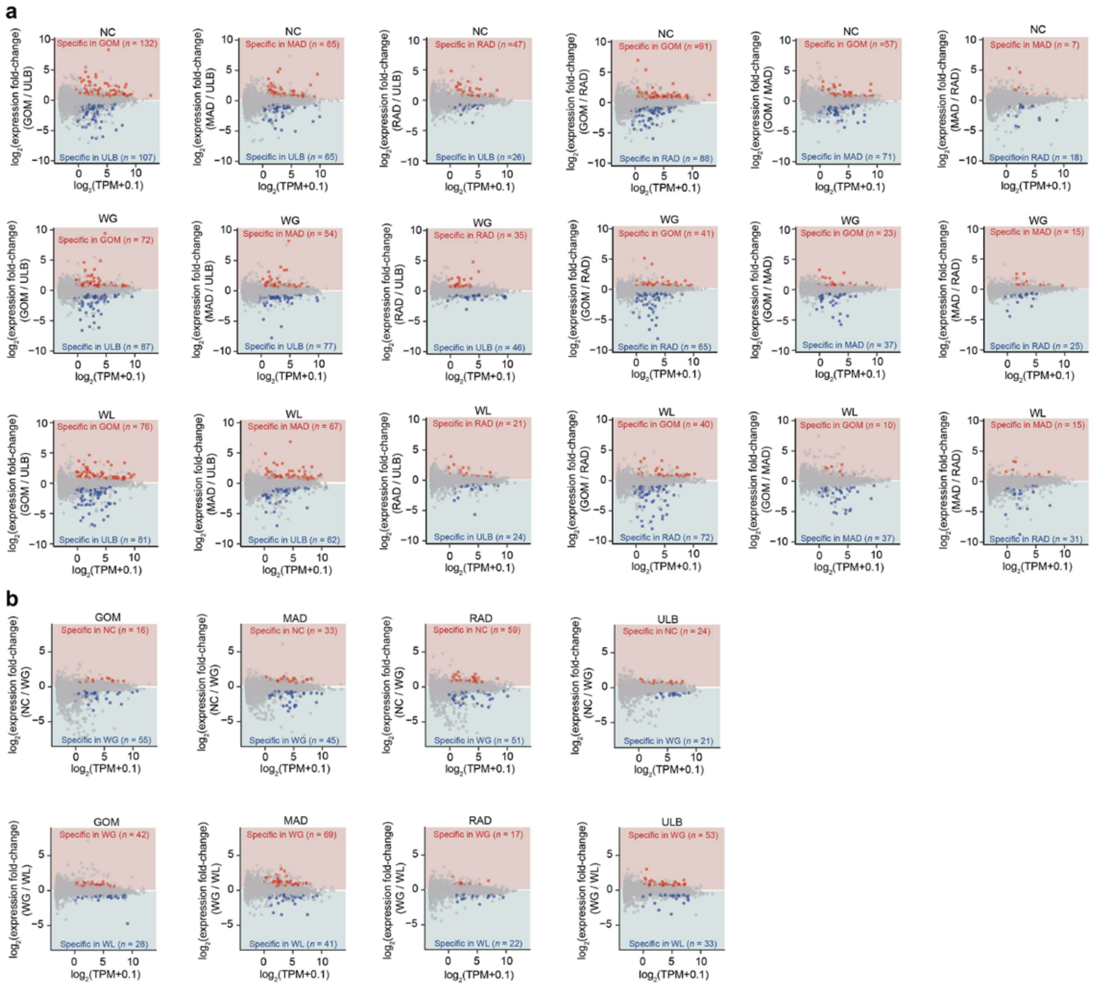

Supplementary Fig. 22. Identification of genes located within AT-specific (a) and group-specific (b) CTCF-mediated loops with specific expression changes. Scatter plot indicates the distribution of expression level versus $\log_2$ fold change of expression between ATs or between treatment groups. We found that genes (average ~105) located in AT-specific CTCF loops showing correspondingly higher expression levels between distinct ATs within a treatment group were generally higher than between treatment groups for a given AT (average ~76).

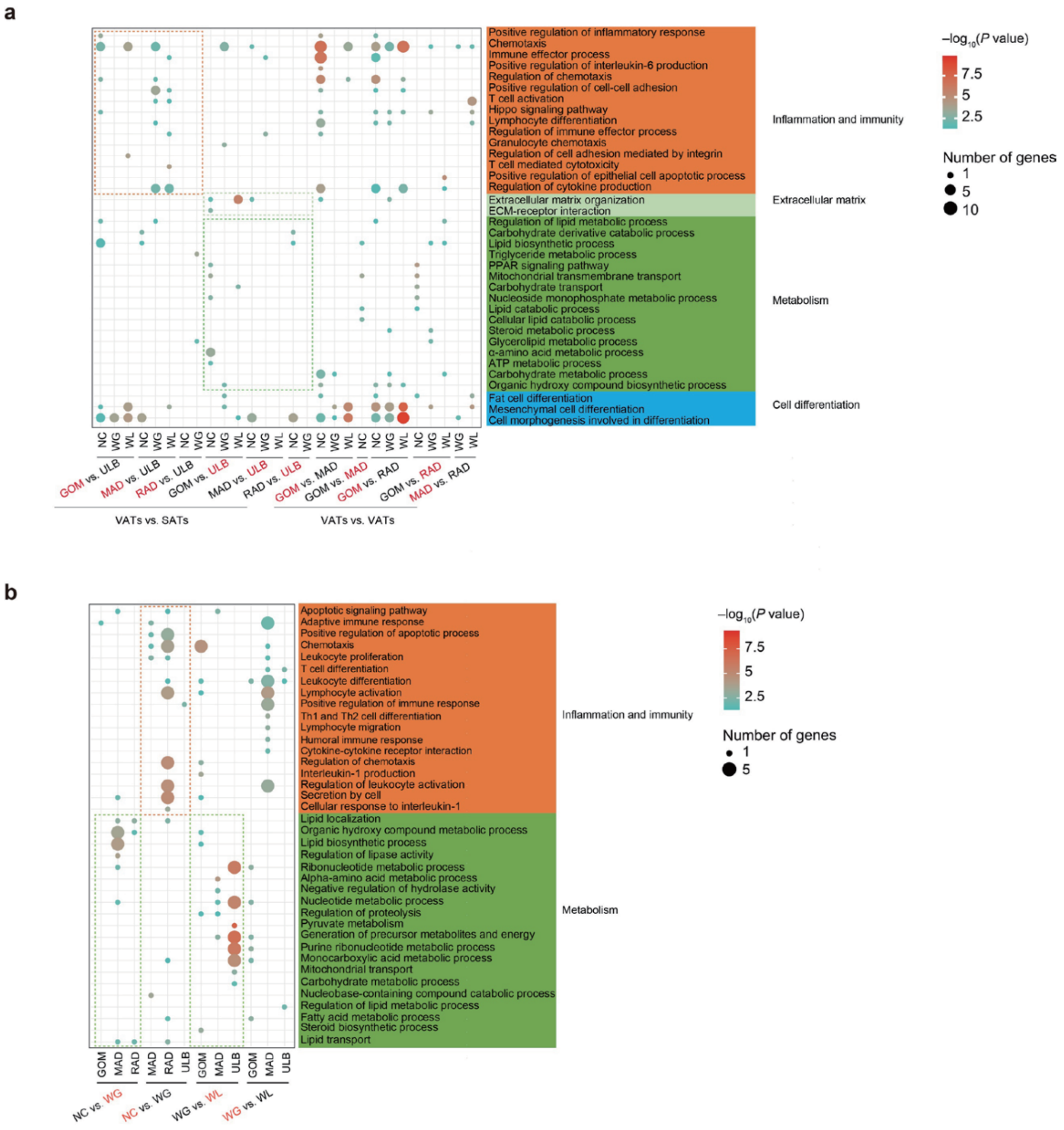

Supplementary Fig. 23. Functional enrichment for genes within AT-specific (a) or treatment-specific (b) CTCF-mediated loops with highly specific expression. Plot showing the top 10 enriched GO terms of genes within AT-specific (**a**) or group-specific (**b**) CTCF loops in pairwise AT or group comparisons. The names highlighted in red for each pairwise comparison represent the tissue or group where the genes are located in specific CTCF loops. The color shades on the Y-axes highlight the categories of GO terms, *i.e.,* inflammation- and immunity-related terms (orange), extracellular matrix (ECM) (light green), metabolism-related terms (green), cell differentiation (blue), and others (n = 141 in **a** panel and n = 38 in **b** panel, not shown in the plot). The size of the dot represents the number of enriched genes, and the dot color represents the -$\log_{10}$(P-value) (unadjusted). P values were calculated based on a one-sided accumulative hypergeometric test.

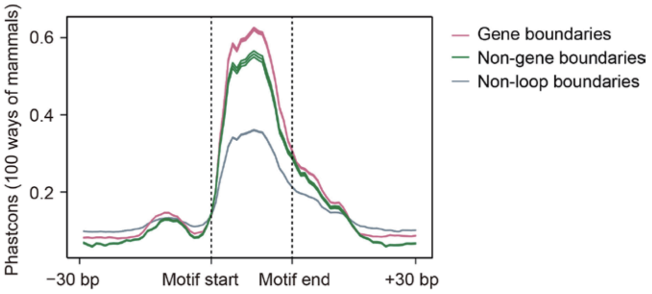

Supplementary Fig. 24. Evolutionary conservation (inferred by Phastcons) of consensus CTCF motifs at boundaries of CTCF-mediated loop boundaries with or without genes and non-boundary regions. The motif region (19 bp in length) is indicated by the dashed box. The shaded area indicates the standard error.

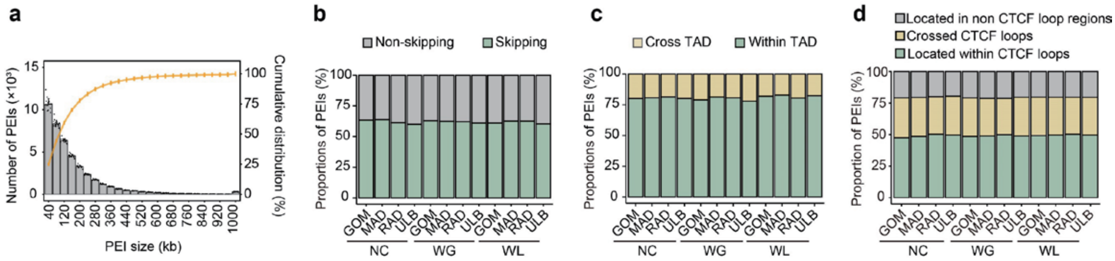

Supplementary Fig. 25. Identification of PEIs using the PSYCHIC algorithm.

a Size distribution of PEIs. PEIs of size ≥ 40 kb were retained. The line represents cumulative distribution. In the boxplot, the internal line indicates the median, the box limits indicate the upper and lower quartiles and the whiskers extend to 1.5 IQR from the quartiles. (n = 18). Error bars represent differences in PEI size among ATs across groups.

b Proportions of gene promoters interacting with the nearest enhancer or skipping at least one enhancer. 38% of promoters interacted with proximal/closest enhancers, while 62% of promoters skip proximal enhancers to interact with more distal enhancers, revealing that promoters and enhancers bridging considerable genomic distances can be located.

c, d Proportions of PEIs within or cross TAD (**c**) and CTCF loops (**d**). As expected, most detected interactions (~66.5%) were within the TADs, while only a small fraction of interactions (~33.5%) crossed TAD boundaries. Among all the PEIs, ~49.28% were located within CTCF loops, ~20.57% were crossed CTCF loops, and ~30.15% were located in non-CTCF loop regions. To test the preference of PEIs located within TADs or CTCF-mediated loops, we generated random/spurious PEI data sets, according to the number and length distribution of observed PEIs for each chromosome. Statistical significance was then assessed by Chi-square Test.

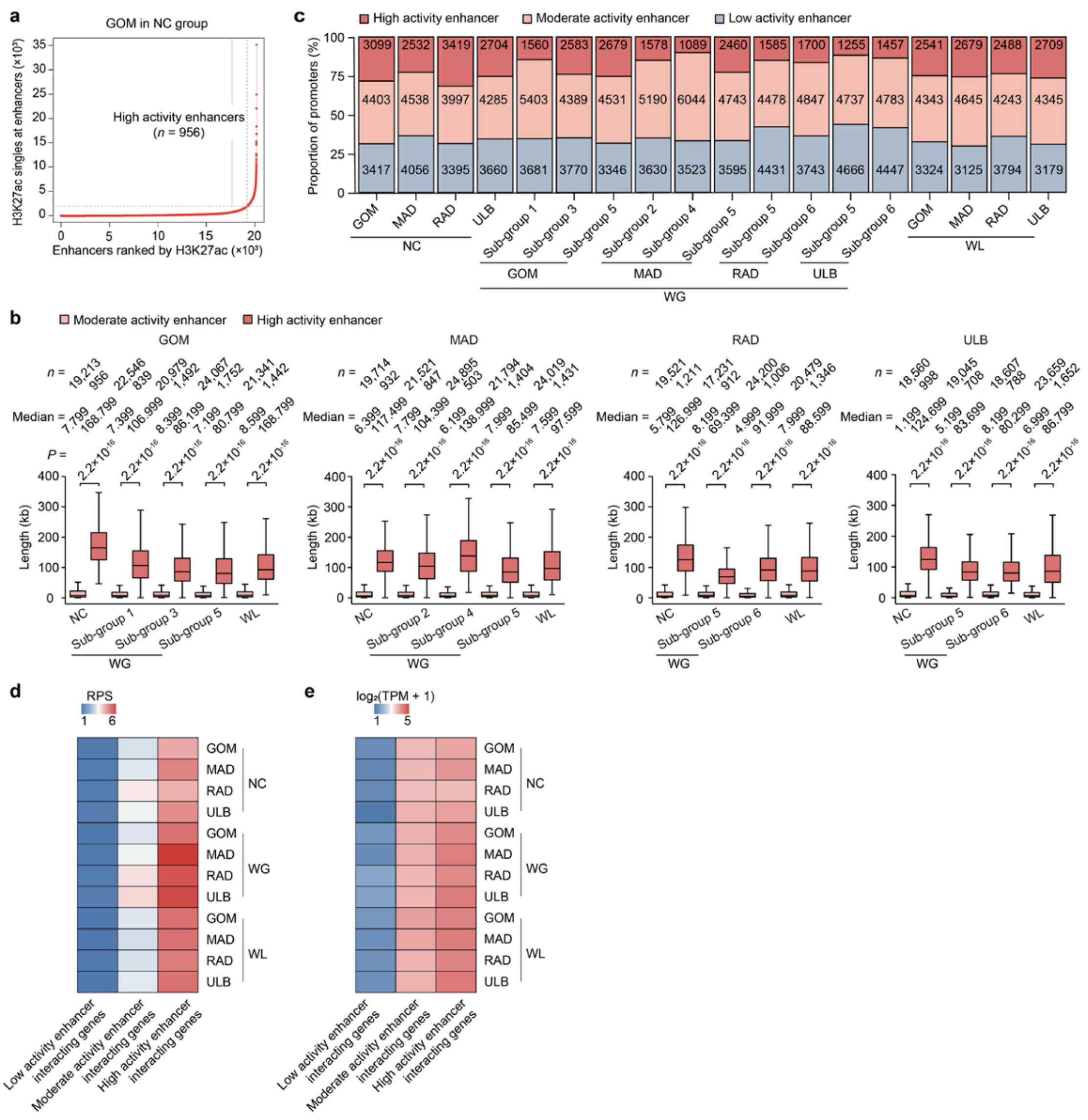

Supplementary Fig. 26. Activities of enhancers associated with gene expression.

a Saturation curves of H3K27ac density in GOM in NC group as an example. The number of ranked moderately active enhancers and enhancers of high-activity (*i.e.*, super-enhancers) by H3K27ac density (x-axis) and their respective densities (y-axis) are plotted. Horizontal dotted lines represent density cutoffs used to classify high-activity enhancers, and vertical dotted lines demarcate high-activity enhancers from moderate-activity enhancers. The total number of high-activity enhancers is noted.

b The distribution of the lengths of moderate-activity and high-activity enhancers for each AT. In the boxplot, the internal line indicates the median, the box limits indicate the upper and lower quartiles and the whiskers extend to 1.5 IQR from the quartiles. The numerical value above each bar indicates the number of enhancers within this group. Statistical significance was determined using a two-sided Wilcoxon rank-sum test.

c The proportion of genes that interacted with high-activity, moderate-activity, and low-activity enhancers.

d, e Expression (**d**) and RPS values (**e**) of genes interacting with enhancers have different

activities. Compared to genes contacted with low-activity enhancers (depleted in H3K27ac peaks; ~17.43% of PEI-associated genes) or moderate-activity enhancers (with moderate H3K27ac peaks; ~21.92%), genes contacted with high-activity enhancers (with broad H3K27ac peaks; ~10.47%) exhibited higher RPS and had increased expression.

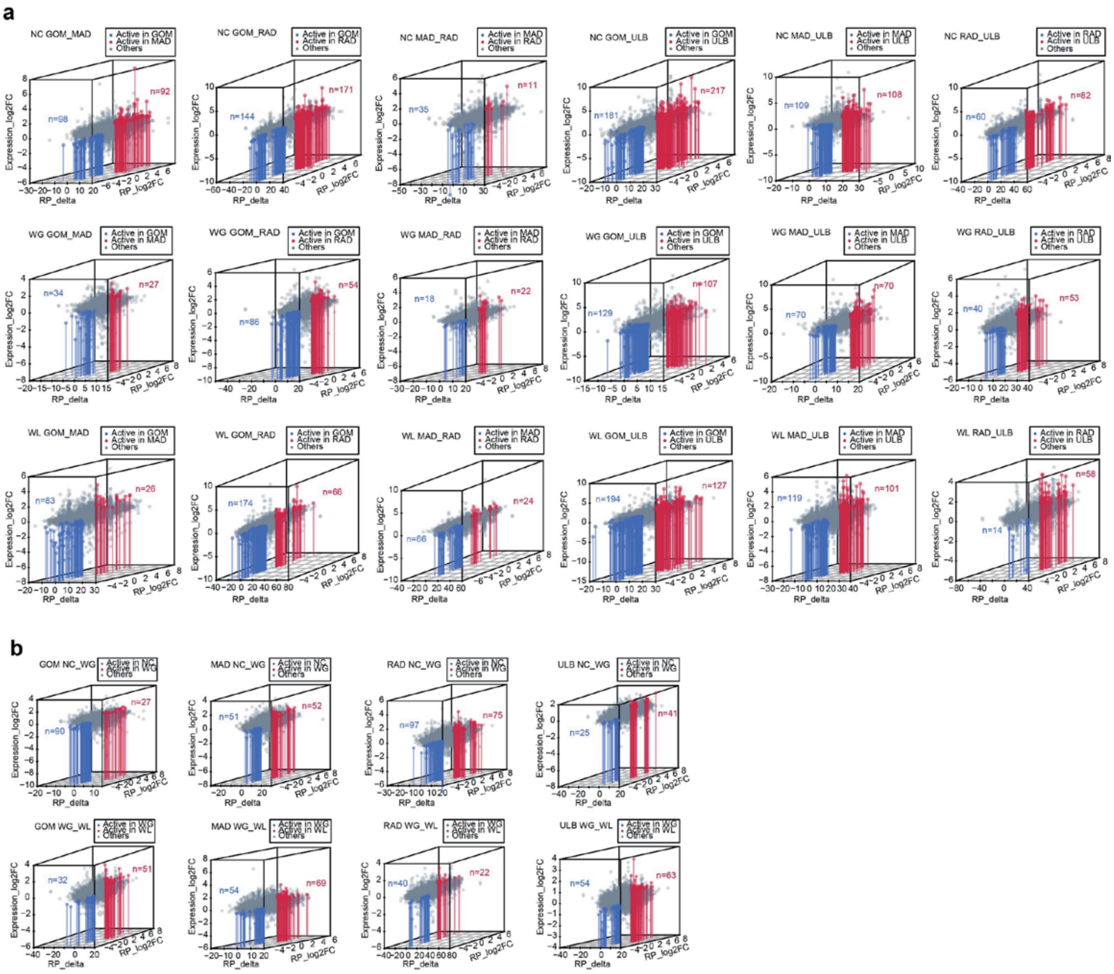

Supplementary Fig. 27. Identification of AT-specific and group-specific genes with covariation between RPS and gene expression. 3D scatter plot indicates the distribution of RPS and expression changes of genes between pairwise AT comparisons in each group (**a**) or between groups for each AT (**b**). In each comparison, color data points with both statistically higher RPS values (fold change [FC] > 1.5, Δ > 2) and statistically up-regulated expression ($\log_2$ FC > 1, FDR < 0.05) were identified as genes with co-variation between RPS and gene expression. The number of co-variation genes is indicated in the plot.

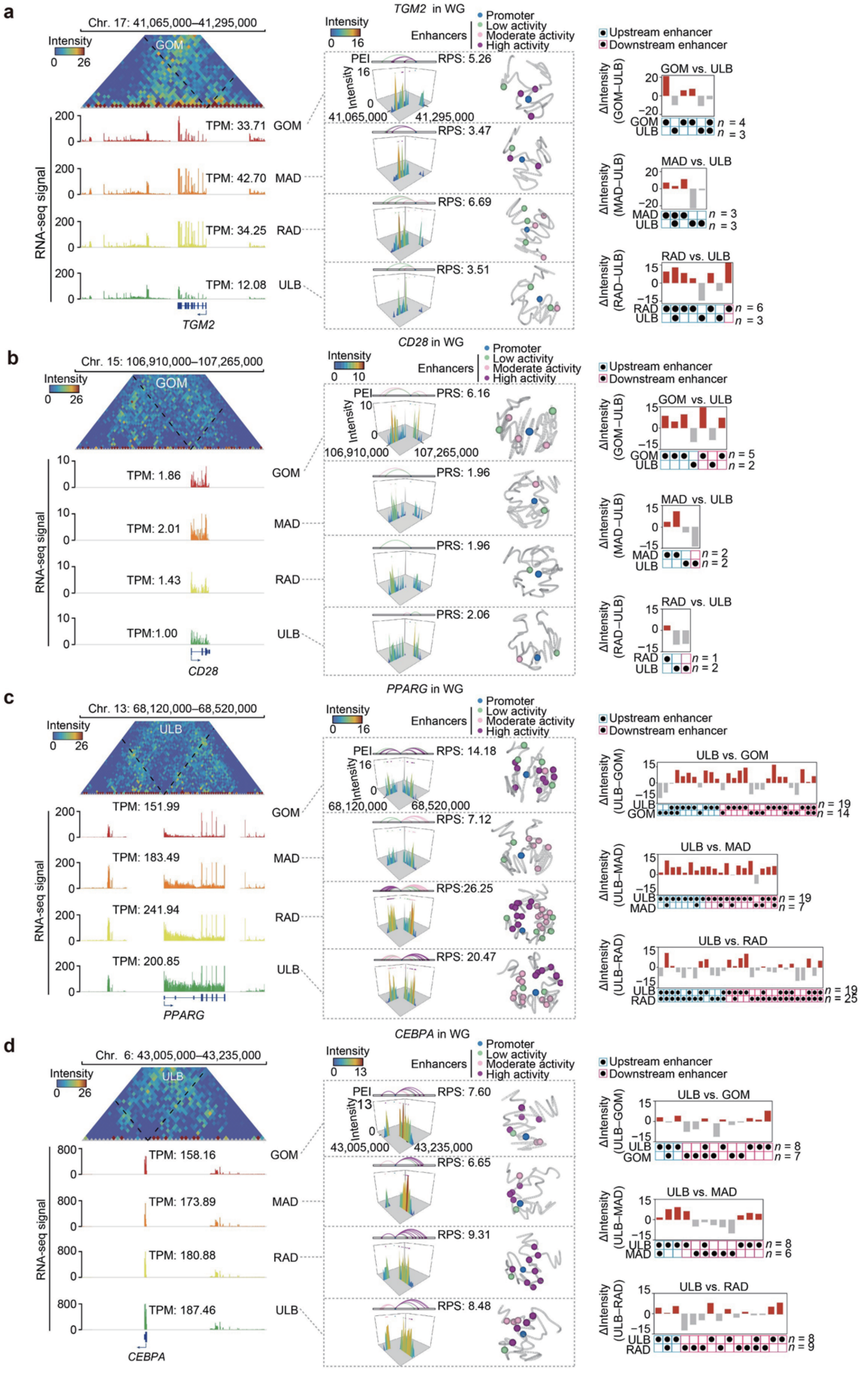

Supplementary Fig. 28. Differential expression and RPS pattern of typical metabolism and pro-inflammation genes between ATs in the WG group.

a, b Schematic representation of PEIs for typical adipogenesis genes, *TGM2* (**a**) and *CD28* (**b**) that are highly abundant in VATs in the WG group.

c, d Schematic representation of PEIs for typical adipogenesis genes, *PPARG* (**c**) and *CEBPA* (**d**) that are highly abundant in SATs in the WG group.

(left) Promoter-centered interactions and expression levels for gene examples across four ATs.

(middle) Interaction metaplots of promoter-centered regions across four ATs.

(right) Difference in PEI intensity between pairwise AT comparisons.

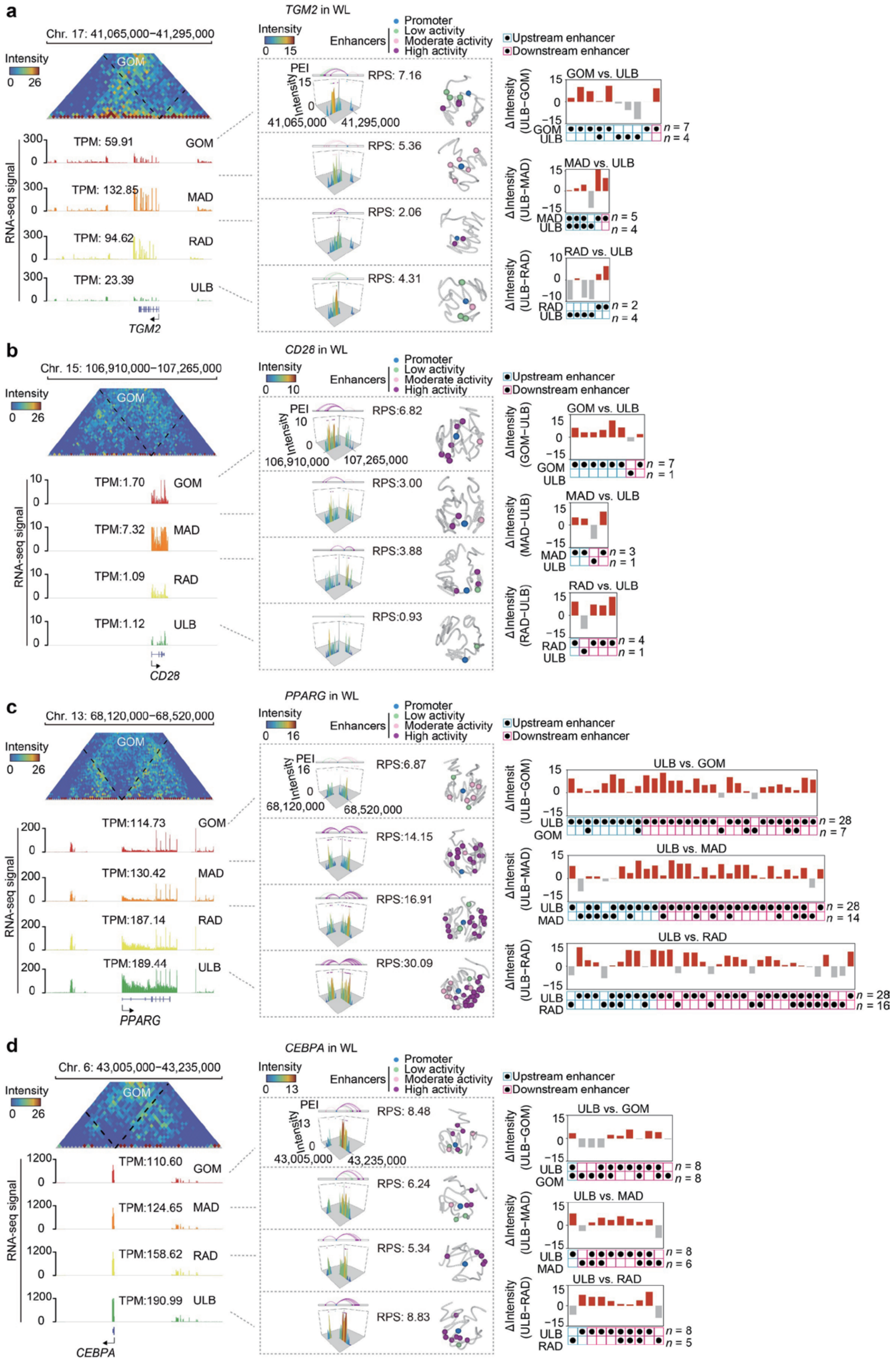

Supplementary Fig. 29. Differential expression and RPS pattern of typical metabolism and pro-inflammation genes between ATs in the WL group.

a, b Schematic representation of PEIs for typical adipogenesis genes, *TGM2* (**a**) and *CD28*

(**b**) that are highly abundant in VATs in the WL group.

c, d Schematic representation of PEIs for typical adipogenesis genes, *PPARG* (**c**) and *CEBPA* (**d**) that are highly abundant in SATs in the WL group.

(left) Promoter-centered interactions and expression levels for gene examples across four ATs.

(middle) Interaction metaplots of promoter-centered regions across four ATs.

(right) Difference in PEI intensity between pairwise AT comparisons.

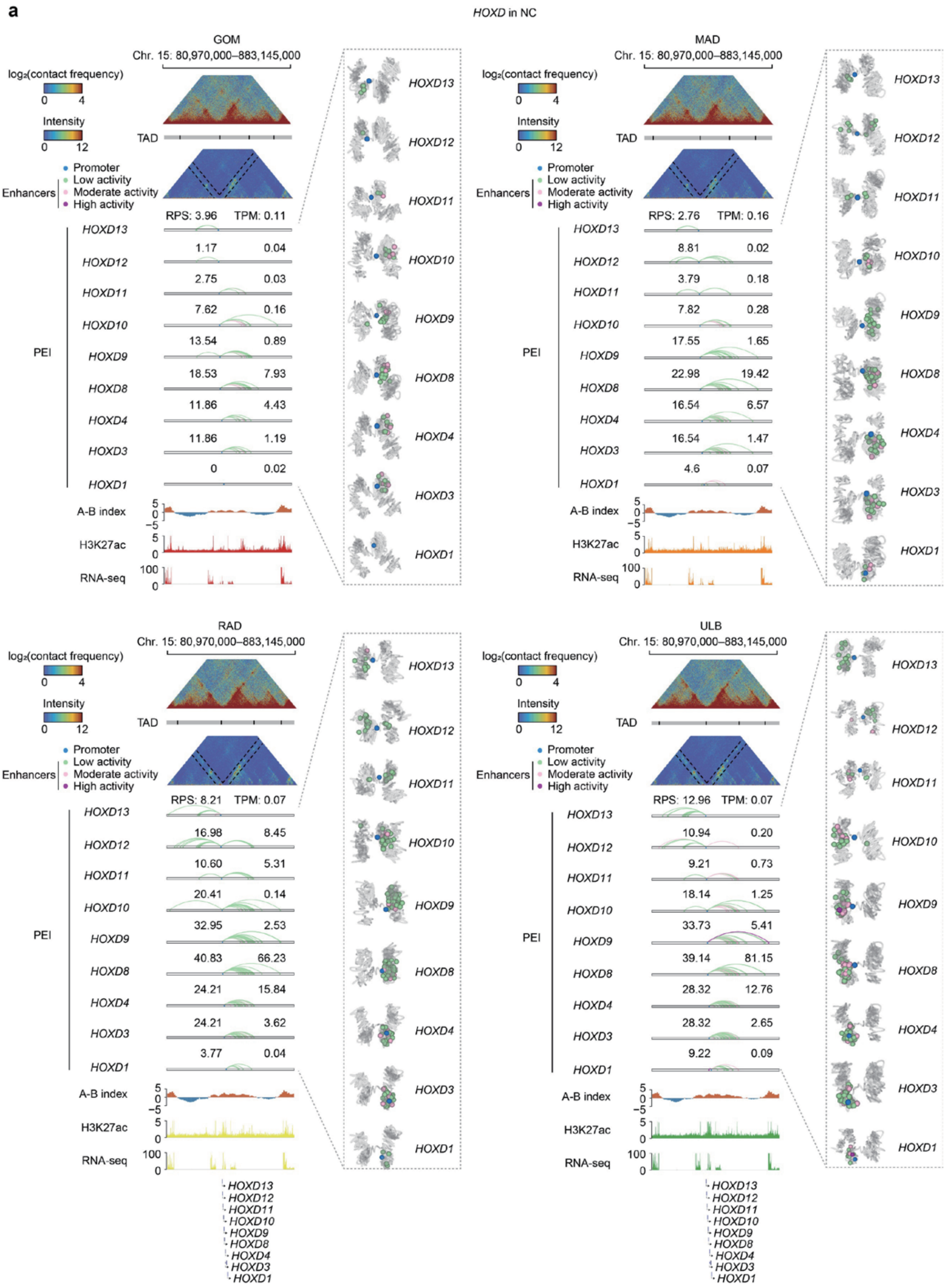
a
HOXD in NC
GOM
MAD
RAD
ULB
Chr. 15: 80,970,000–883,145,000
log2(contact frequency)
Intensity
TAD
Enhancers
Promoter
Low activity
Moderate activity
High activity
PEI
A-B index
H3K27ac
RNA-seq
RPS: 3.96 TPM: 0.11
RPS: 2.76 TPM: 0.16
RPS: 8.21 TPM: 0.07
RPS: 12.96 TPM: 0.07
HOXD13
HOXD12
HOXD11
HOXD10
HOXD9
HOXD8
HOXD4
HOXD3
HOXD1

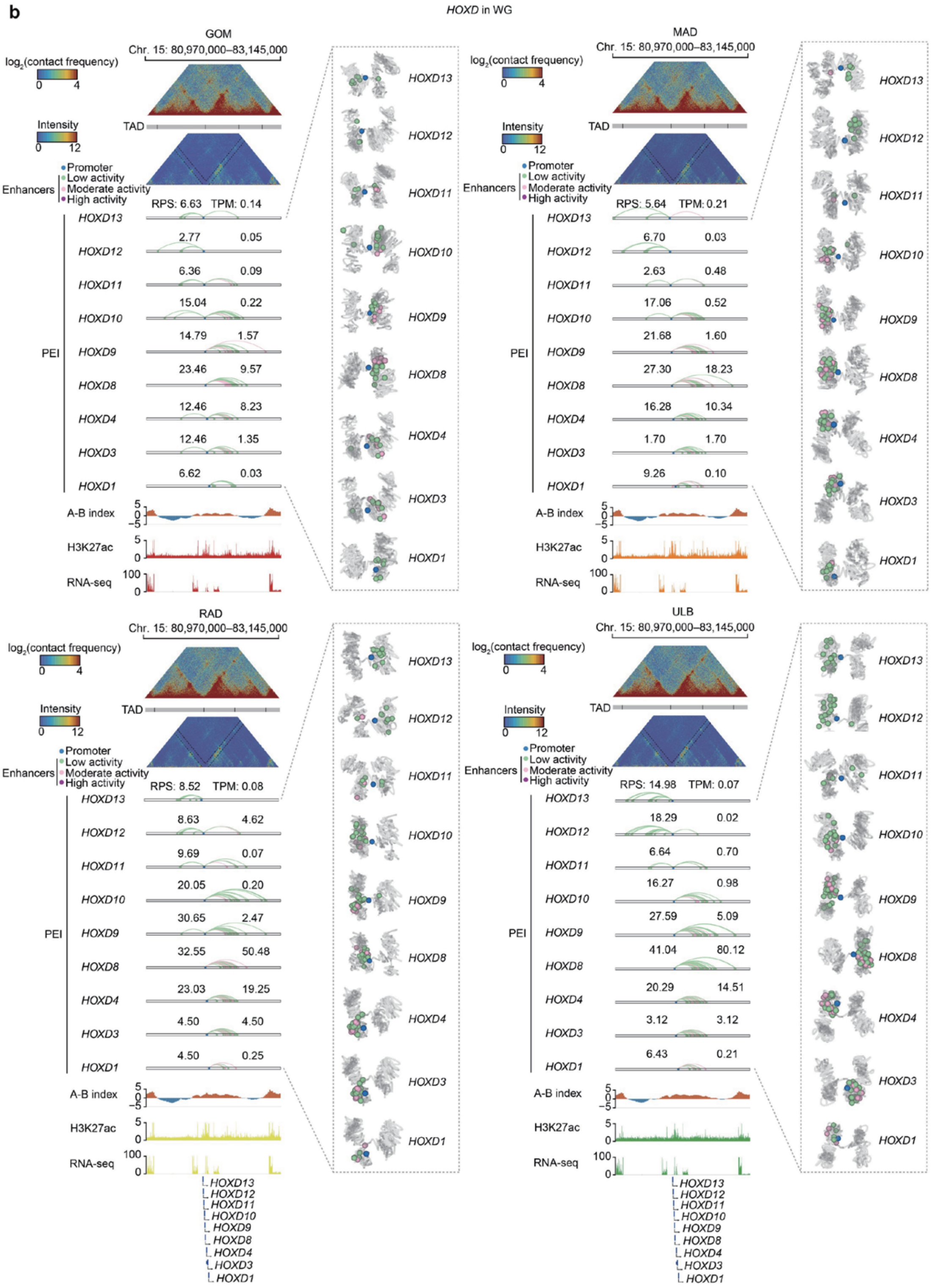
b
HOXD in WG
GOM
MAD
RAD
ULB
Chr. 15: 80,970,000–83,145,000
log2(contact frequency)
TAD
Intensity
Promoter
Low activity
Moderate activity
High activity
Enhancers
PEI
A-B index
H3K27ac
RNA-seq
HOXD13
HOXD12
HOXD11
HOXD10
HOXD9
HOXD8
HOXD4
HOXD3
HOXD1
RPS: 6.63 TPM: 0.14
RPS: 5.64 TPM: 0.21
RPS: 8.52 TPM: 0.08
RPS: 14.98 TPM: 0.07

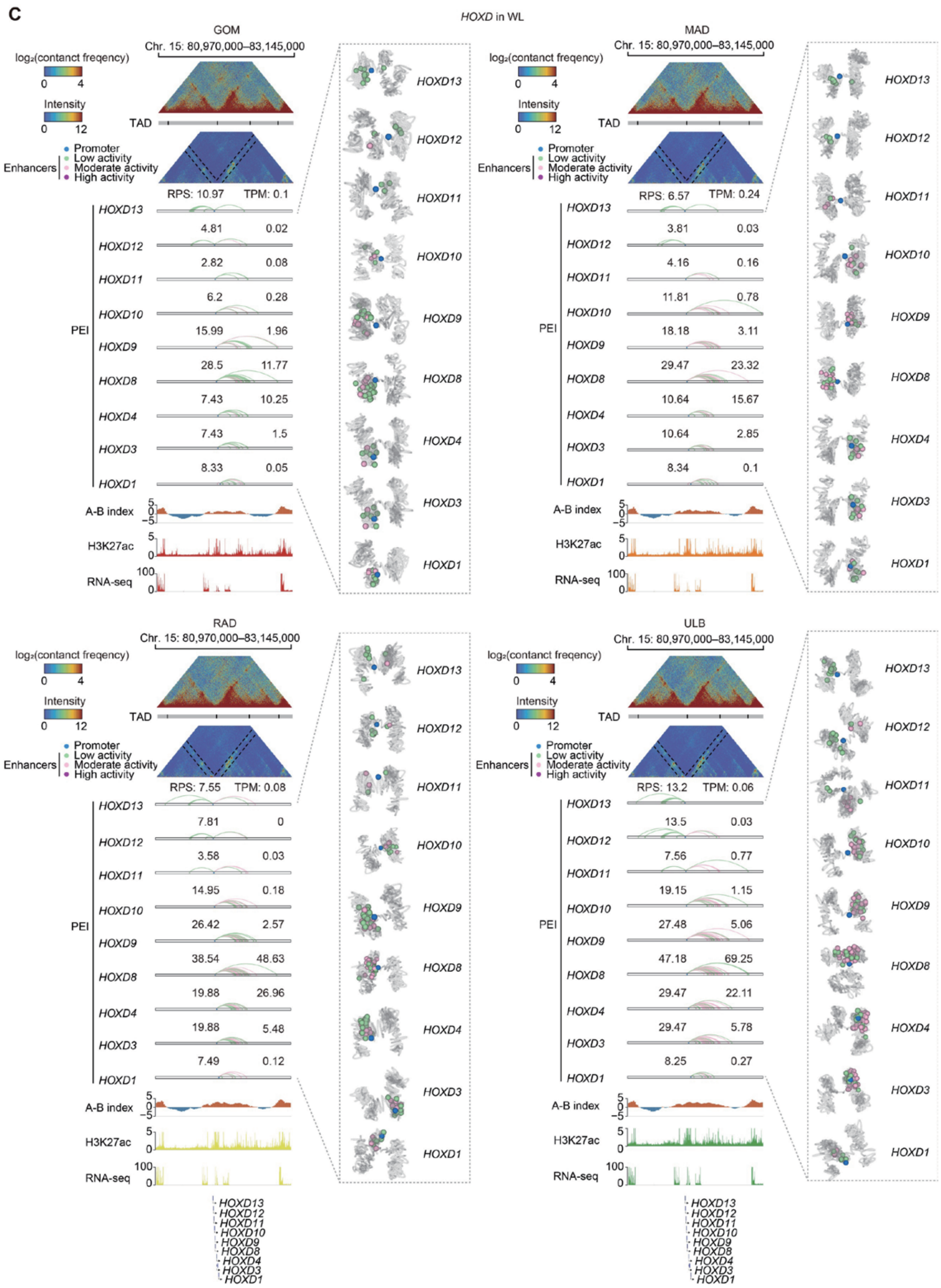

Supplementary Fig. 30. Schematic representation of PEIs for nine *HOXD* genes that are highly abundant in SAT in the NC (**a**), WG (**b**), and WL (**c**) groups. Top to bottom: (top) Hi-C maps of TAD structures; black vertical lines indicate TAD boundaries. Hi-C maps of promoter-centered interactions. (middle) Schematic representation and 3D structural models of PEIs. RPS and gene expression levels are shown. Promoters: blue spheres, low-activity enhancers: green spheres, moderate-activity enhancers: pink spheres, high-activity enhancers: purple spheres, and PEIs: connecting lines. (bottom) A-B index pattern, ChIP-seq signals of H3K27ac, and expression levels in RNA-seq data.

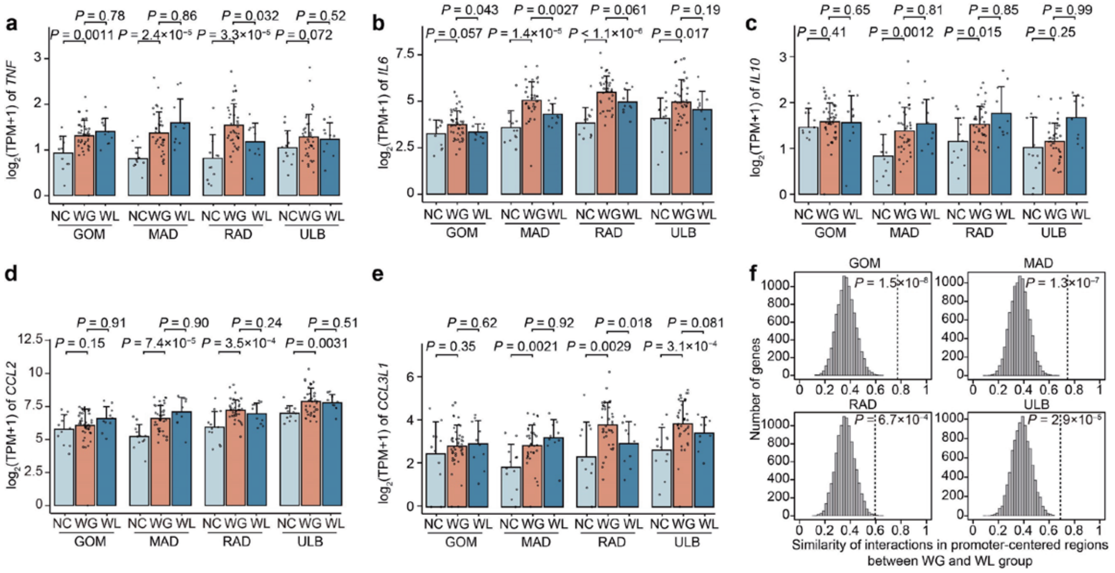

Supplementary Fig. 31. Pattern of expression and RPS of five well-documented obesogenic memory genes.

a-e Expression pattern of five typical obesogenic memory genes (*TNF*, *IL6*, *IL10*, *CCL2*, and *CCL3L1*) in each AT across WG and WL treatment groups. Data are presented as mean ± SD. GOM: NC n = 11, WG n = 45, WL n = 10; MAD: NC n = 12, WG n = 36, WL n = 10; RAD: NC n = 12, WG n = 39, WL n = 10; ULB: NC n = 12, WG n = 38, WL n = 10. P values determined by one-sided Wilcoxon rank-sum test.

f Similarity (Pearson's r) of interactions in promoter-centered regions (± 500 kb from the promoter bin). Histogram shows the distribution of the similarity of interactions of randomly resampling five genes from the 470 inflammatory genes (random number = 10,000). The dotted line indicates the similarity of interactions of five markers of 'obesogenic memory' in mice (*TNF*, *IL6*, *IL10*, *CCL2*, and *CCL3*). P values are calculated by one-sided permutation test.

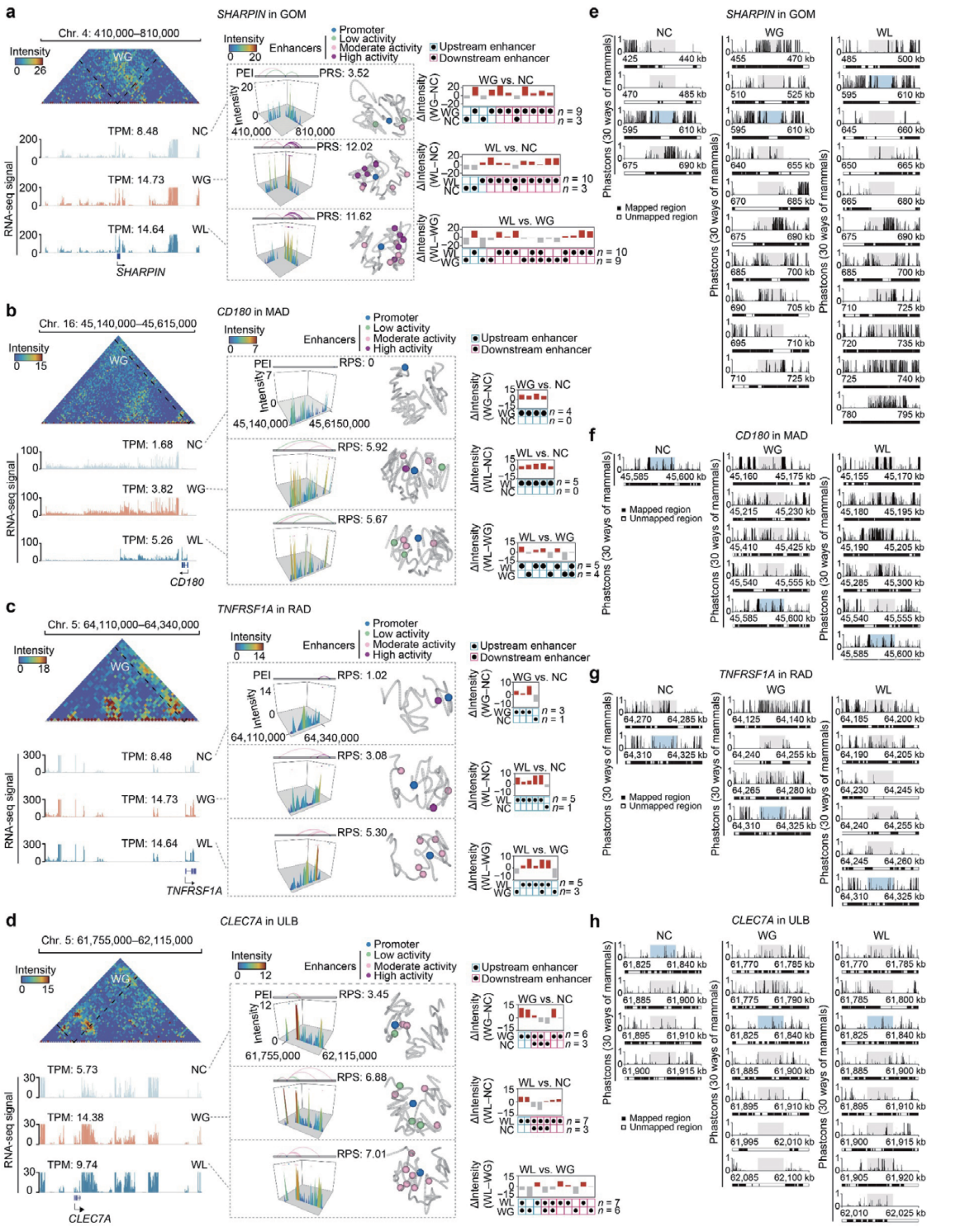

Supplementary Fig. 32. Expression patterns and RPS of candidate obesogenic memory genes.

a–d Schematic representation of PEIs for typical genes, including *SHARPIN* in GOM **(a)**, *CD180* in MAD **(b)**, *TNFRSF1A* in RAD **(c)**, and *CLEC7A* in ULB **(d)** that show highly expressed obesogenic memory established in the WG group compared to NC group which remains stable in the WL group. From left to right: Hi-C maps indicating promoter-centered interactions (upper left) and gene expression levels (lower left). Interaction metaplots of promoter-centered regions and 3D structural models (middle). Promoters: blue spheres;

low-activity enhancers: green spheres; moderate-activity enhancers: pink spheres; high-activity enhancers: purple spheres; and PEIs: connecting lines. Difference in PEI intensity between pairwise groups comparisons (right).

e-f The conservation information across multiple mammals (30-way phastCons value) in the promoters and its interacting enhancers for *SHARPIN* in GOM, *CD180* in MAD, *TNFRDF1A* in RAD, *CLEC7A* in ULB during weight gain or loss. The tracks show conservation information alongside the designated 5-kb regions with 5-kb up- and down-stream regions. The shadow box shows the 5-kb region of promoters (blue) or enhancers (grey). Enhancers/promoter are presented from top to bottom based on their linear genomic position, corresponding to **a-d**, respectively.

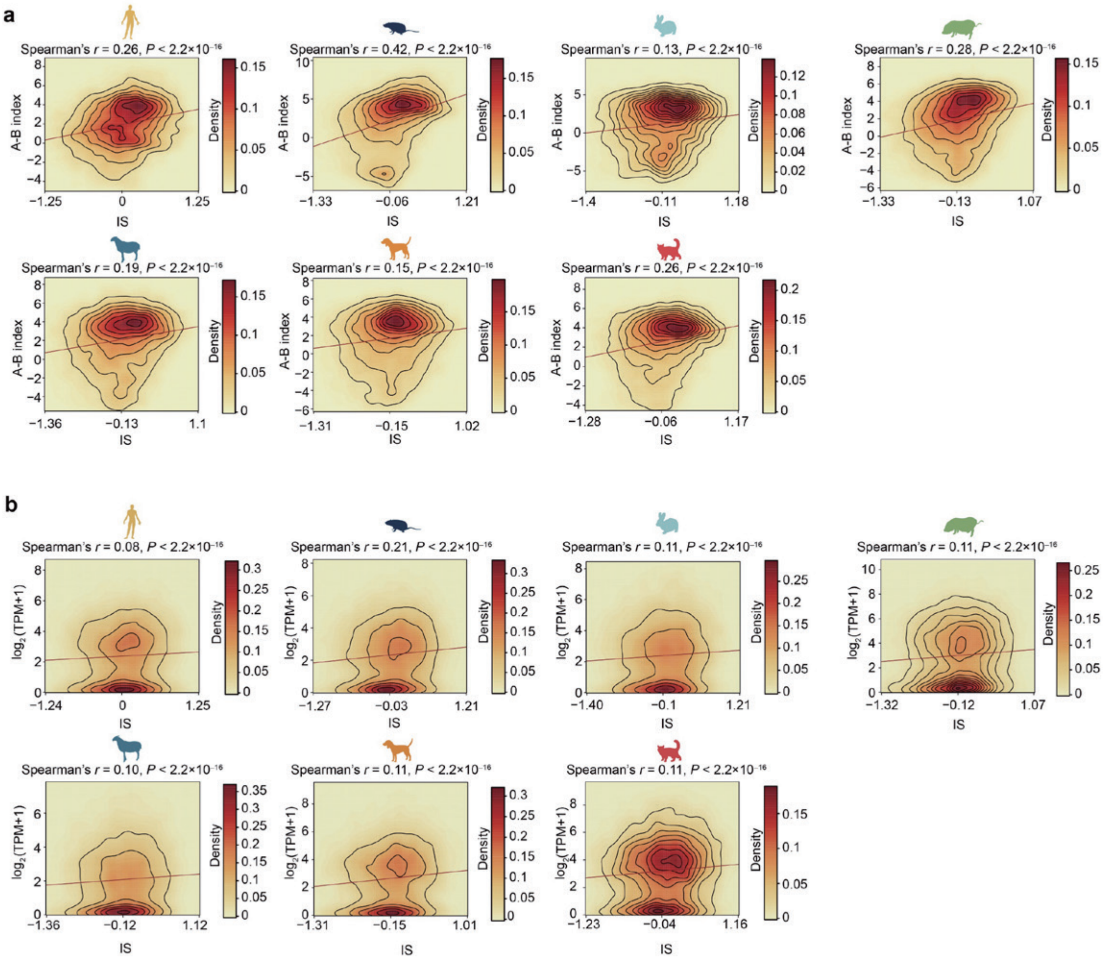

Supplementary Fig. 33. Local spatial context is positively correlated to compartmental status and transcriptional activity.

a Correlation between IS and A-B index across all the bins (20-kb in length) for all seven mammalian species. The statistical significance of the two-sided *P* value was calculated using hypothesis testing.

b Correlation between IS and gene expression across all gene bins (20-kb in length) in each species. The statistical significance of the two-sided *P* value was calculated using hypothesis testing.

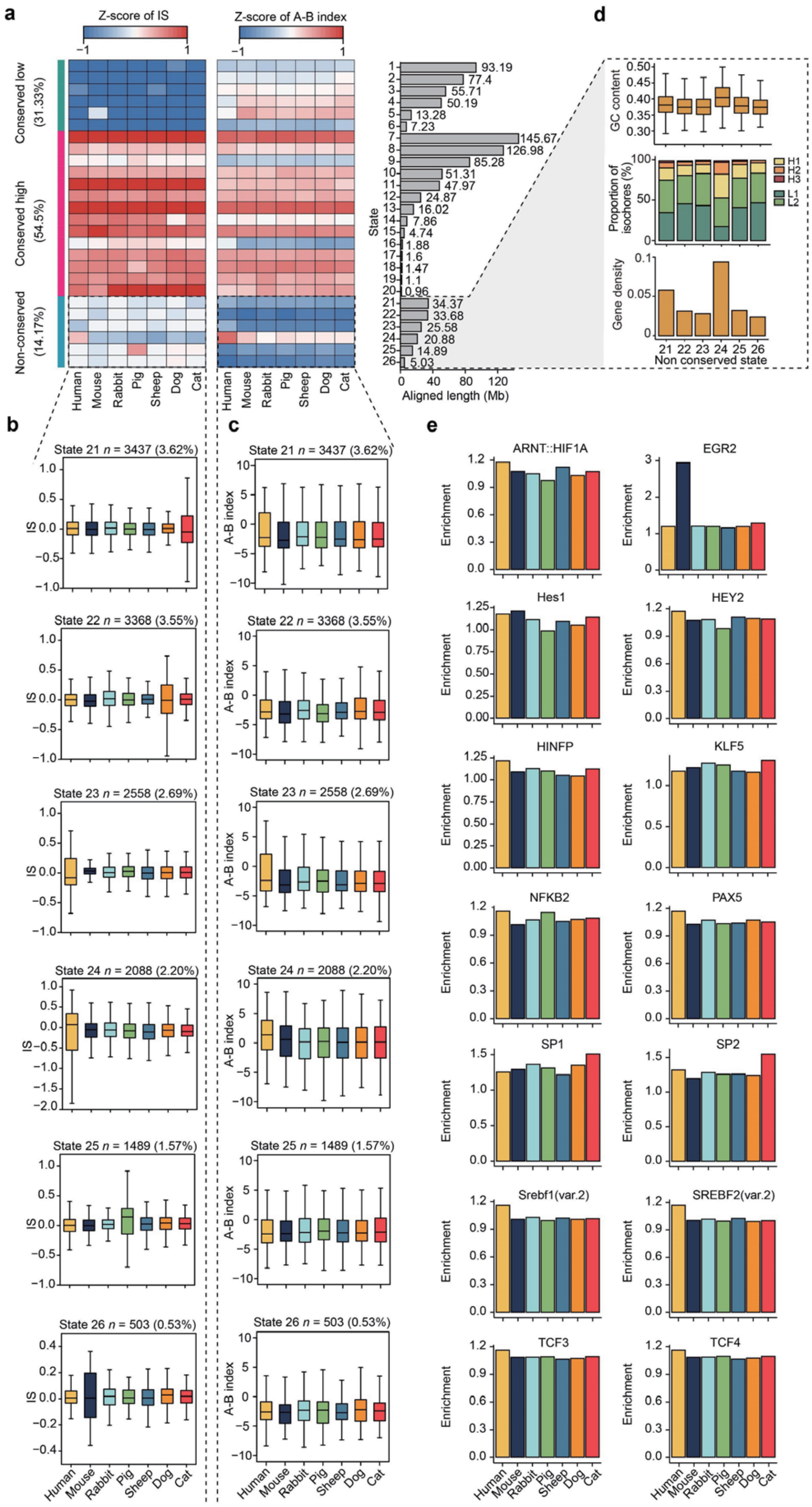

Supplementary Fig. 34. Conservation pattern of IS value across seven species.

a The conserved IS index groups were clustered using cosine similarity ($r \geq 0.85$ for any two states in the same group). (left panel) Twenty-six states could be grouped into conserved IS, including conserved high IS or low IS, and the remaining states were classified as non-conserved IS. (middle panel) The normalized AB compartment status distribution is also shown for each state. (right panel) The length of genomic regions of each state is shown on the right. The color key indicates the Z-score transformed IS value and AB index. The functional enrichment for genes embedded in conserved and non-conserved regions is shown in **Supplementary Data 8**.

b, c Boxplot showed the IS value (**b**) and AB compartment (**c**) for six non-conserved states. In the boxplot, the internal line indicates the median, the box limits indicate the upper and lower quartiles and the whiskers extend to 1.5 IQR from the quartiles

d Comparisons of genomic features across six non-conserved IS states. (top) Box plot indicates GC content for each state. In the boxplot, the internal line indicates the median, the box limits indicate the upper and lower quartiles and the whiskers extend to 1.5 IQR from the quartiles. 21: $n = 3437$; 22: $n = 3368$; 23: $n = 2558$; 24: $n = 2088$; 25: $n = 1489$; 26: $n = 503$. (middle) Distribution of isochores according to GC levels for each state. We divided the human genome into five families of isochores, in order of increasing GC levels: L1 (GC content < 37%), L2 (37~41%), H1 (41~46%), H2 (46~53%), and H3 (> 53%). (bottom) Gene density for each non-conserved state. Higher GC content and gene density in state 24 in humans with a pattern similar to that of the *Alu* element (**Fig. 4d**) suggested that *Alu* (generally GC-rich) sequences are related to regions with more chromatin interactions regulating the transcription of species-specific genes.

e Enrichment of 14 TFs specifically enriched in state 24 (corresponding to **Fig. 4e, f**) across homologous regions in other species.

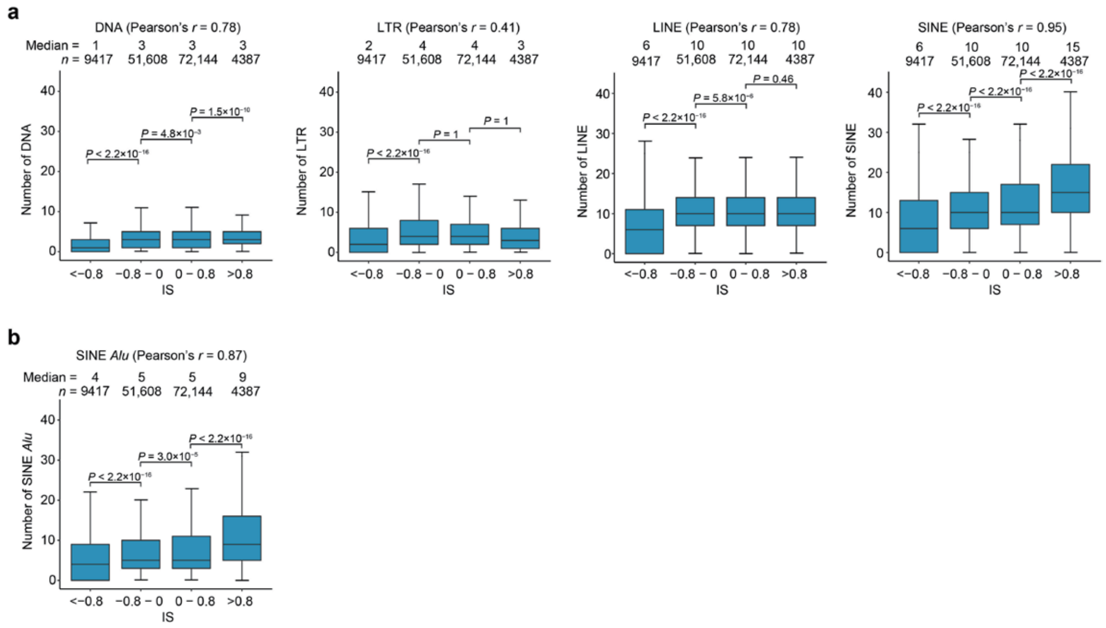

Supplementary Fig. 35. Correlation between IS level and different TE families.

a Correlation between IS level and different TE families. We divided bins (10-kb in length) with IS values into four intervals and analyzed the number of each of the four TE families in each IS interval. In the boxplot, the internal line indicates the median, the box limits indicate the upper and lower quartiles and the whiskers extend to 1.5 IQR from the quartiles. *P* values determined by one-sided Wilcoxon rank-sum test. SINE density is positively correlated with IS levels, and this trend for LINE, DNA, or LTR cannot be easily observed.

b Correlation between IS level and *Alu* families. In the boxplot, the internal line indicates the median, the box limits indicate the upper and lower quartiles and the whiskers extend to 1.5 IQR from the quartiles. *P* values determined by one-sided Wilcoxon rank-sum test. *Alu* density showed obviously increasing trend with IS values.

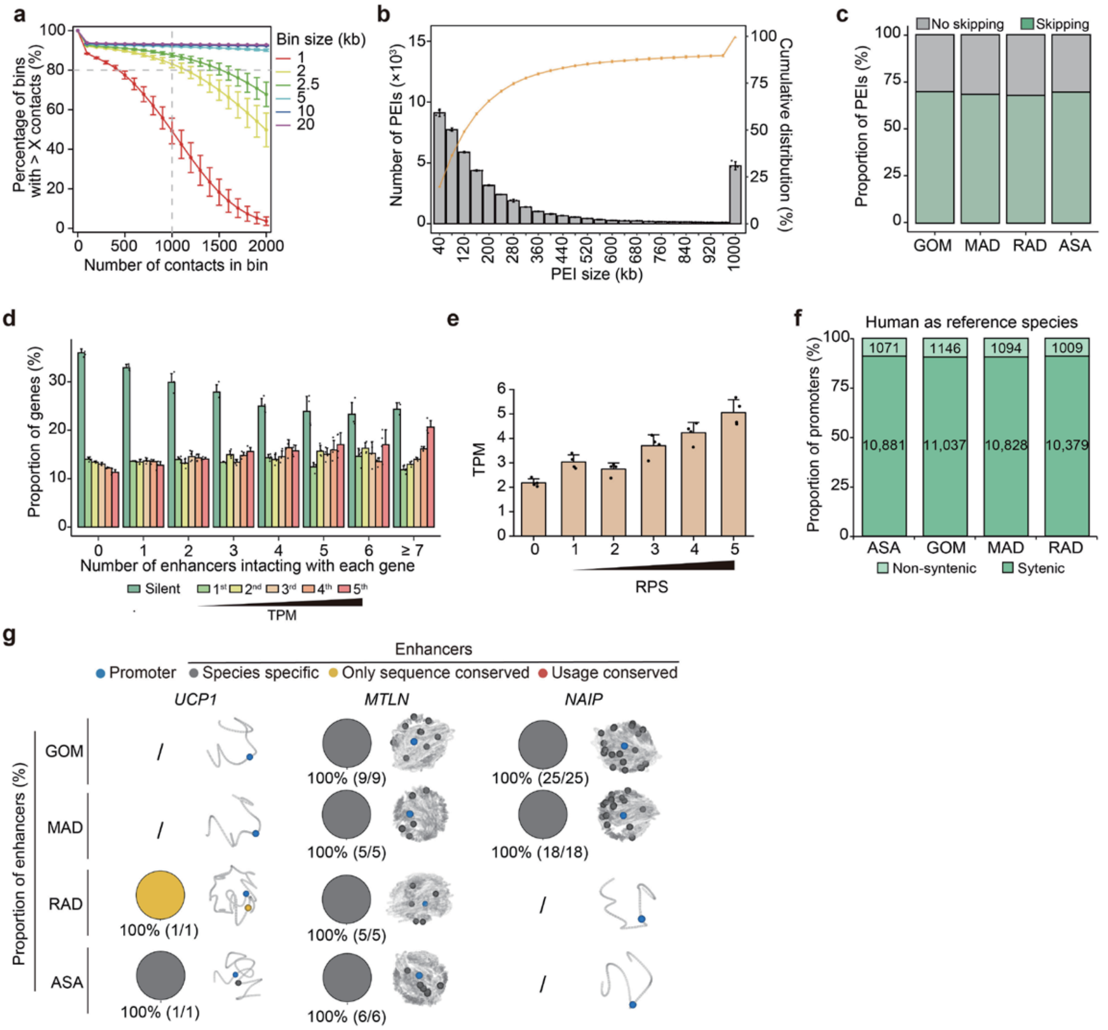

Supplementary Fig. 36. Identification and comparison of PEIs in human ATs.

a Resolution of the merged intra-chromosomal Hi-C contact matrices of replicates for each AT in normal conditions, reached a maximum resolution of ~2 kb. An average of 82.25 % of loci at a 5-kb size have at least 1,000 contacts in each AT. Data are presented as mean values ± SD (n = 4).

b Size distribution of PEIs. PEIs of size ≥ 40 kb were retained. The line represents cumulative distribution. Error bars represent differences in PEI size among ATs. Data are presented as mean values ± SD (n = 4).

c Proportions of gene promoters interacting with the nearest enhancer or skipping at least one enhancer. 30.69% of promoters interacted with proximal/closest enhancers, while 69.31% of promoters skip proximal enhancers to interact with more distal enhancers.

d Proportions of gene promoters from each expression category interacting with zero to more than six enhancers. Data are presented as mean values ± SD (n = 4).

e Gene expression was positively associated with RPS. Genes with RPS > 0 were equally divided into five percentiles. Data are presented as mean values ± SD (n = 4), and dots represent the values of each AT.

f Proportion of human gene promoters comparable to those in pigs. LiftOver information of

genes/promoters that interacted with at least one enhancer in each human AT. To assess the conservation of enhancers in sequence and usage, we only focused on the genes with promoters (5-kb bin embedded in the TSS) that LiftOver could transfer from pigs (as the ‘query’ species) to humans (as the ‘reference’ species).

g Enhancer conservation for typical human-specific genes (*UCP1, MTLN*, and *NAIP*).

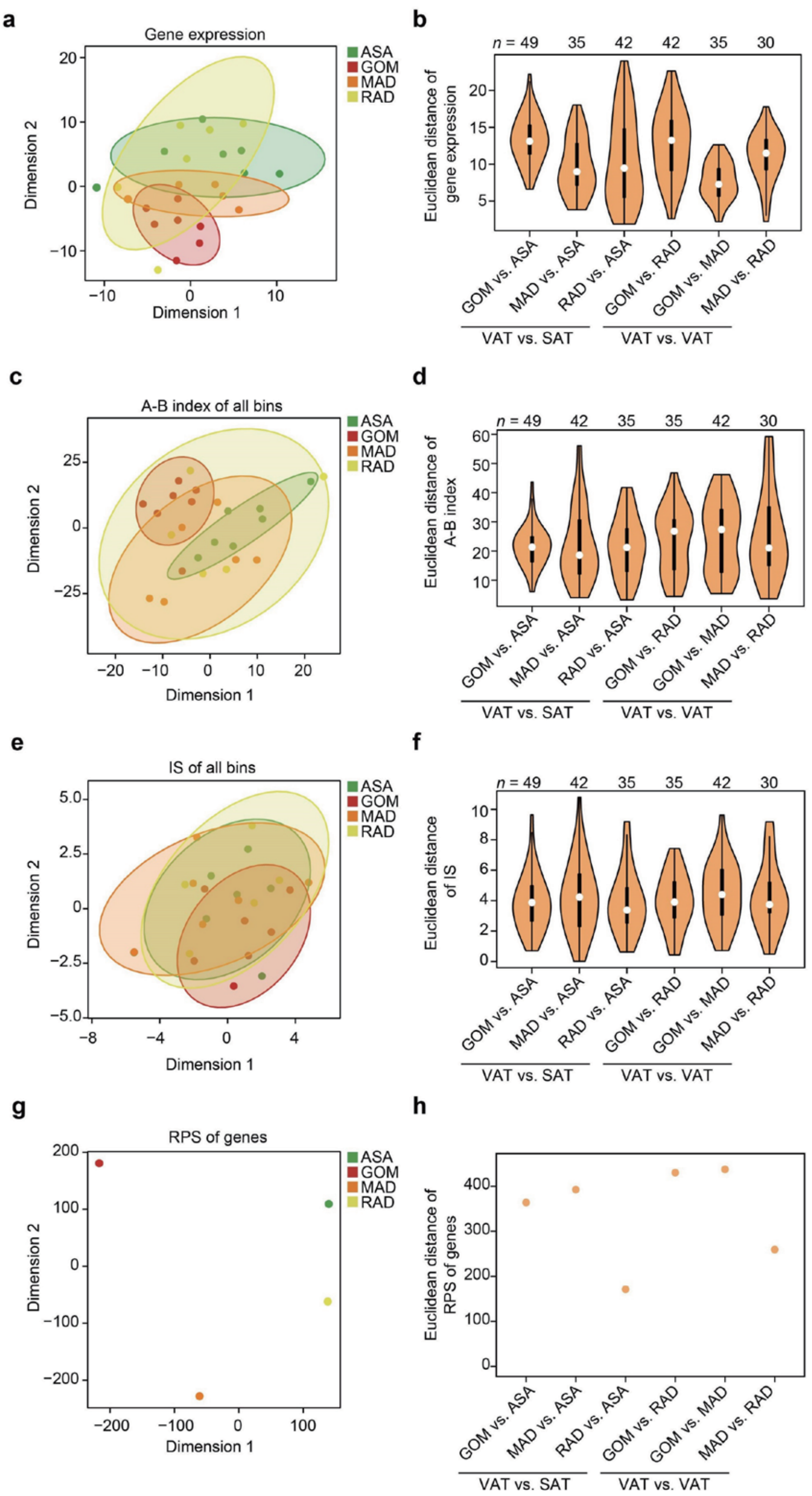

Supplementary Fig. 37. Comparison of transcriptional, PRS, and local spatial context (A/B index and IS values) variation between ATs in humans.

a, c, e, g t-SNE plots based on gene expression (**a**), global AB compartment (**c**), IS values (**e**), and gene RPS (**g**).

b, d, f, h Violin plot showing the distance between pairwise ATs from the tSNE plot. The pattern showing ATs across samples is not obvious compared to that in pigs, because of the objective intrinsic difference between human individuals/donors. In the violin plot, the

internal dot indicates the median, the box limits indicate the upper and lower quartiles and the whiskers extend to 1.5 IQR from the quartiles.

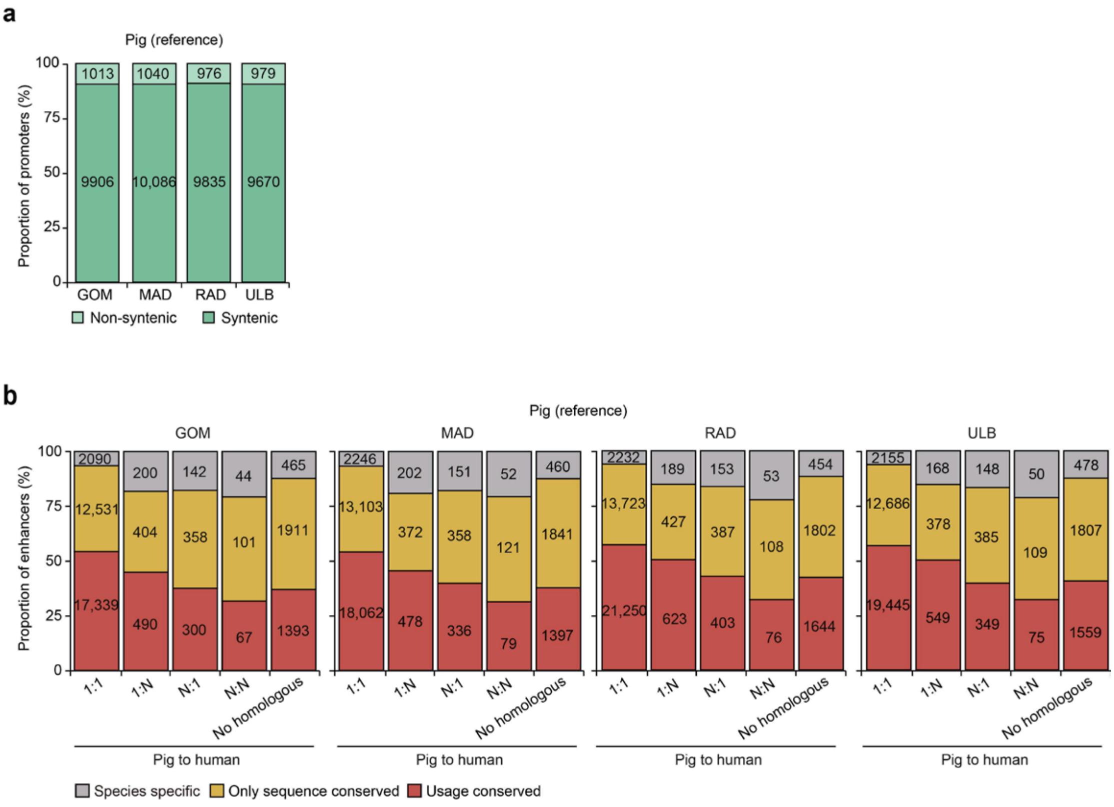

Supplementary Fig. 38. Inter-species conservation of pig enhancers between single-copy orthologs and other orthologs in each AT.

a LiftOver information of genes/promoters that interacted with at least one enhancer in each pig AT.

b Percentage distribution of enhancers that are conserved in both sequence and usage for different types of orthologs between pigs (‘reference’) and humans (‘query’) in each AT. As described in the main text, when using pigs as reference species for humans, we found that single-copy orthologs contained enhancers that showed higher sequence conservation (93.63%) and usage conservation (59.39%) compared to those that interacted with multi-copy orthologs (sequences: 81.51%; usage: 48.63%) or pig-specific genes (sequences: 87.79%; usage: 44.83%). Conservation of enhancers was assessed in both sequence and usage as described in **Fig. 5c and Methods**.

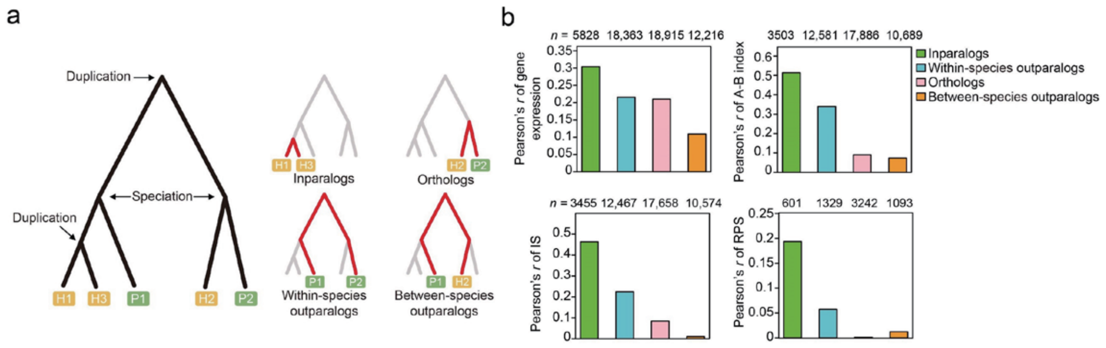

Supplementary Fig. 39. Similarity in expression, local spatial context, and RPS of different types of homologies.

a Four different types of homology relations, including orthologs, inparalogs, within-species outparalogs, and between-species outparalogs.

b Barpot showing differences in pairwise gene expression between different types of homologies. The similarity was calculated as the Pearson's correlation of TPM, A-B index, IS, and RPS for homologs between pigs and humans across four distinct ATs.

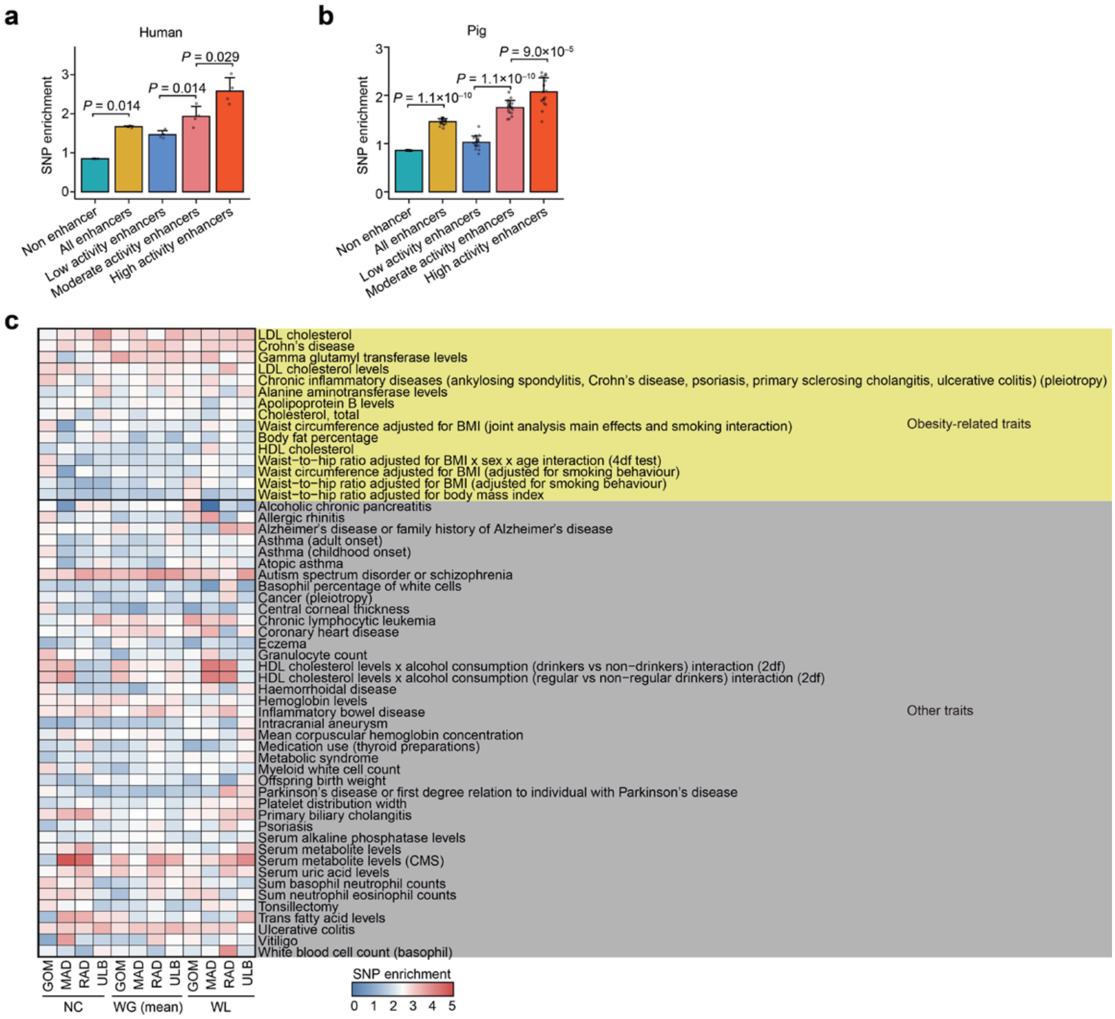

Supplementary Fig. 40. Enhancers in ATs were enriched for trait-associated non-coding SNPs, especially for obesity-related traits.

a, b Comparison of enrichment in non-coding regions for obesity-related trait-associated, non-coding SNPs in non-enhancer sequences, found only in enhancers (*i.e.,* all enhancers), low-activity enhancers, moderate-activity enhancers, and high-activity enhancers in Human ATs (**a**). Data are presented as mean values ± SD (n = 4). Enrichment analysis for trait-associated, non-coding human SNPs that LiftOver could transfer to pigs (**b**). Data are presented as mean values ± SD (n = 18). Identification of enhancer activity based on H3K27ac binding peaks and calculation of SNP enrichment (defined as the percent of SNPs contained in the percent of the genome covering a given region (*i.e.,* different types of enhancers) (see **Methods** for details). DNA sequence variations (*i.e.*, human non-coding SNPs) associated with obesity-related traits (n = 55) were enriched in the enhancers of ATs in the human genome (mean SNP enrichment scores in enhancers vs. non-enhancer regions: 1.67 vs. 0.85, P = 2.20×10^{-10}, one-tailed Wilcoxon test), which was more prominent in high-activity enhancers (mean SNP enrichment score: 2.58)**.** SNP variations were also enriched in enhancers of ATs in the pig genome (mean SNP

enrichment scores in enhancer vs. non-enhancer regions: 1.38 vs. 0.87, $P = 2.20 \times 10^{-10}$, one-tailed Wilcoxon rank-sum test).

c Heatmap of SNP enrichment for all trait-associated non-coding SNPs in all enhancers in each AT across the NC, WG, and WL groups. The top 20 traits merged with the highest SNP enrichment for enhancers in each AT is shown. Traits can be empirically classified into obesity-related traits (in yellow) and other traits (in grey).

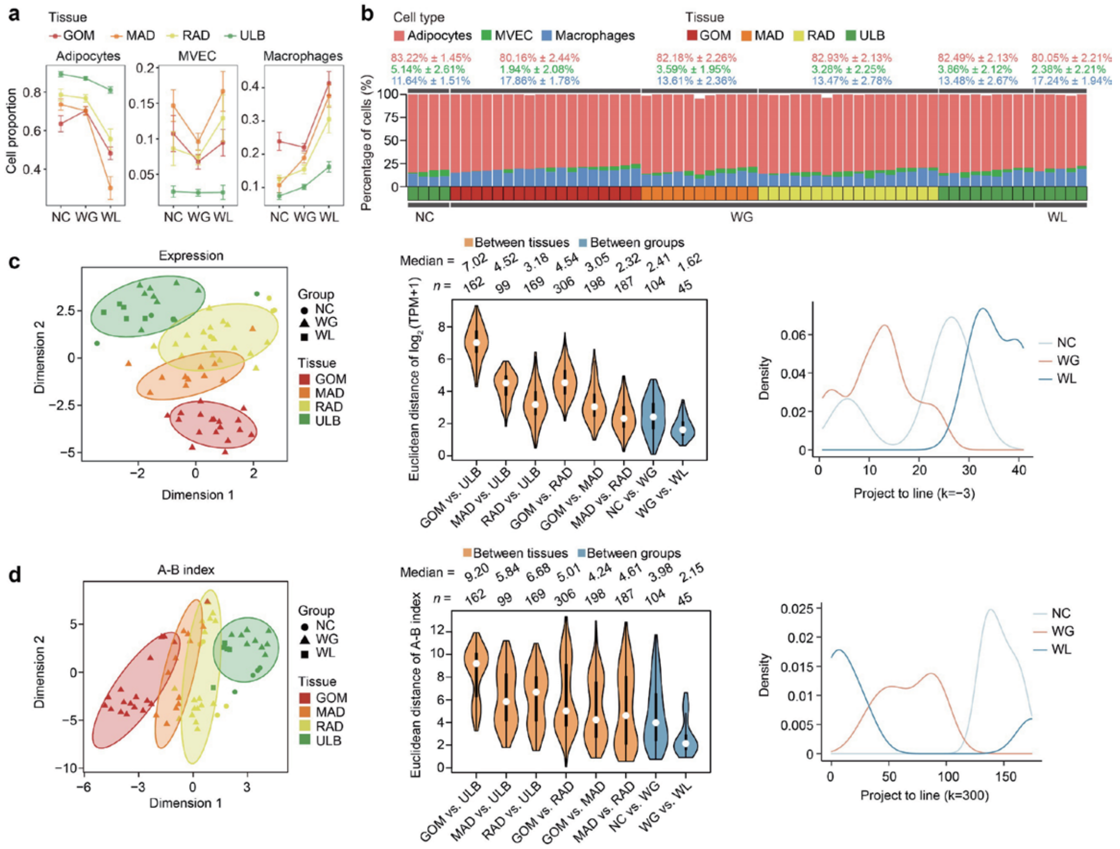

Supplementary Fig. 41. Comparison of differences in gene expression and A/B compartmentalization between adipose depots and between treatment groups with similar cellular composition.

a The cellular proportions of adipocytes, microvascular endothelial cells (MVECs), and macrophages during weight gain or weight loss for each adipose depot. Data are presented as mean ± SD. GOM: NC *n* = 11, WG *n* = 45, WL *n* = 10; MAD: NC *n* = 12, WG *n* = 36, WL *n* = 10; RAD: NC *n* = 12, WG *n* = 39, WL *n* = 10; ULB: NC *n* = 12, WG *n* = 38, WL *n* = 10. Dots represent mean values across replicates. The interval lines show standard deviation across replicates.

b All 64 samples with highly similar cell composition (including adipocytes, macrophages, and MVECs) of four ATs in the WG group and ULB from all three treatment groups. The above numbers indicate the proportion of each cell type in each AT across groups. Data show means ± SD.

c t-SNE clustering of the 64 samples with similar cell compositions using gene expression data (left). In the t-SNE plot, ellipses indicate AT samples with similar profiles, constructed at a probability of 0.85. Violin plot shows the Euclidean distance of gene expression between samples (middle). In the violin plot, the internal dot indicates the median, the box limits indicate the upper and lower quartiles and the whiskers extend to 1.5 IQR from the quartiles. The percentage distribution of projection distance (derived from t-SNE plot) between each dot and a given line (y=kx, k=-3) across groups in ULB (right).

d t-SNE clustering of the same 64 samples using A-B index (left). Ellipses indicate AT samples with similar profiles, constructed at a probability of 0.85. Violin plot shows the Euclidean distance of A-B index between samples (middle). In the violin plot, the internal dot indicates the median, the box limits indicate the upper and lower quartiles and the whiskers extend to 1.5 IQR from the quartiles. The percentage distribution of projection distance (derived from t-SNE plot) between each dot and a given line (y=kx, k=300) across groups in ULB (right).

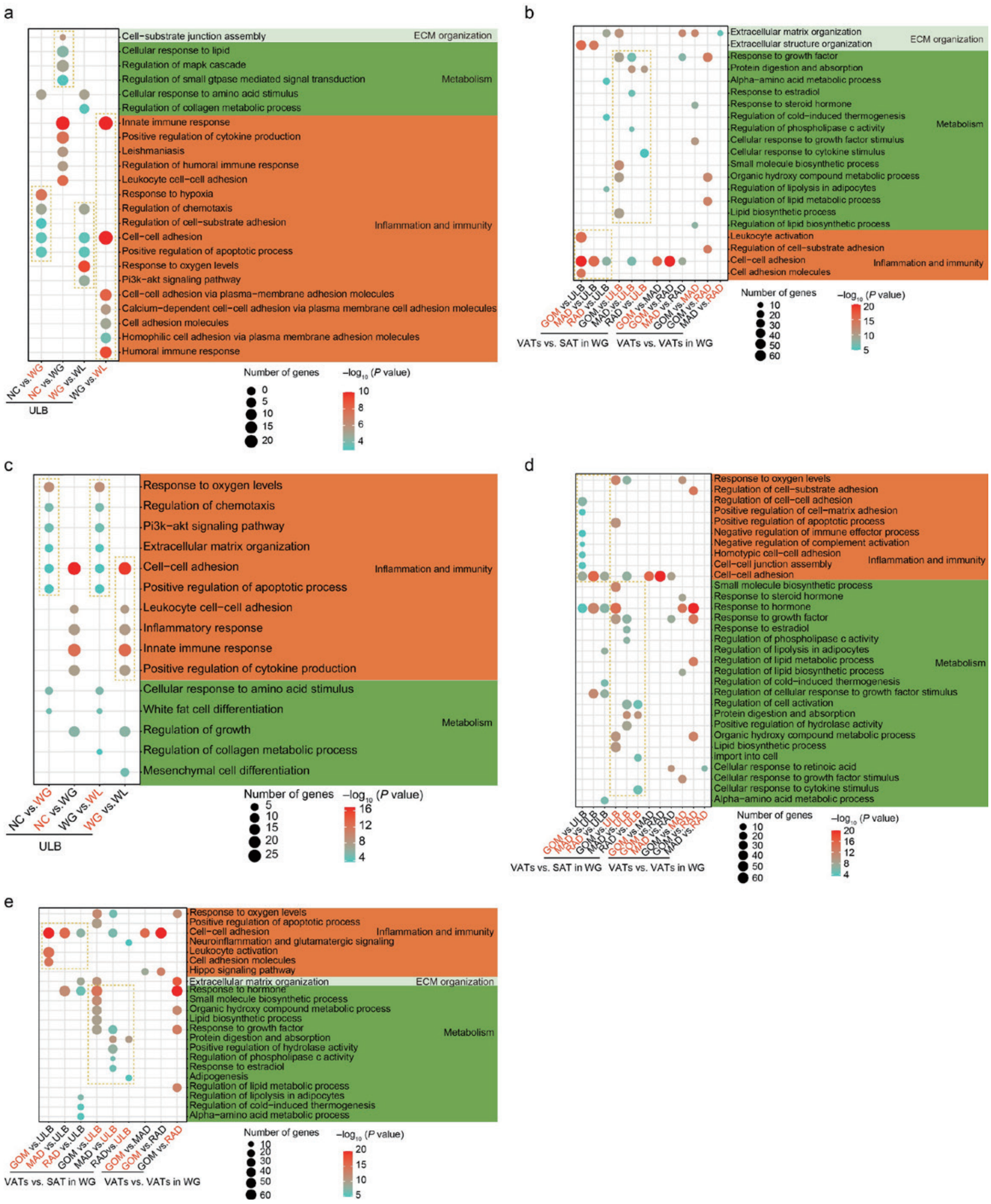

Supplementary Fig. 42. Functional enrichment for differentially expressed genes (a,b), A/B compartmentalization (c,d), and RPS changes (e) between groups or between ATs with similar cell composition.

a Plot showing the top 20 enriched GO terms of DE PCGs in NC-WG and WG-WL comparisons for ULB. The names highlighted in red for each pairwise comparison indicate the group in which the genes were highly expressed. GO term color scheme: dark green, metabolism-related terms; pale green, extracellular matrix (ECM) organization-related terms; orange, inflammation- and immunity-related terms; and others (n = 38, not shown in the plot). Dot size is proportional to the number of enriched genes; dot color represents

the -$\log_{10}$(P-value) (unadjusted). P values were calculated based on a one-sided accumulative hypergeometric test.

b Plot showing the top 10 enriched GO terms of DE PCGs in pairwise comparisons of AT with similar cellular composition in the WG group. Pairwise comparisons are between VAT and SAT or within VATs. Names highlighted in red indicate the tissue in which the genes were highly expressed. GO term color scheme: dark green, metabolism-related terms; pale green, extracellular matrix (ECM) organization-related terms; orange, inflammation- and immunity-related terms; and others (n = 86, not shown in the plot). Dot size is proportional to the number of enriched genes; dot color represents the -$\log_{10}$(P-value) (unadjusted). P values were calculated based on a one-sided accumulative hypergeometric test.

c,d Plot showing the top 20 enriched GO terms in comparisons between groups **(c)** and between ATs **(d)** with similar cellular composition. The presentation is the same as in **a** and **b**, but show A/B compartments of genes. Other terms (27 terms for **c**, 87 for **d**) are not shown.

e, Plot showing the top 20 enriched GO terms in comparisons between ATs with similar cellular composition. The presentation is the same as that in **a** and **b**, but for AT-specific genes with covariation between RPS and gene expression. Other terms (n = 63) are not shown. Number of group-specific genes with covariation between RPS and gene expression were generally fewer than 50, and thus insufficient for functional enrichment analysis.

Supplementary References

1. Reilly, S. M. & Saltiel, A. R. Adapting to obesity with adipose tissue inflammation. *Nat. Rev. Endocrinol.* **13**, 633 (2017).
2. Rosen, E. D. & Spiegelman, B. M. What we talk about when we talk about fat. *Cell* **156**, 20–44 (2014).
3. De Jonge, L. et al. Effect of diet composition and weight loss on resting energy expenditure in the POUNDS LOST study. *Obesity* **20**, 2384–2389 (2012).
4. Ibrahim, M. M. Subcutaneous and visceral adipose tissue: structural and functional differences. *Obes. Rev.* **11**, 11–18 (2010).
5. Tchkonia, T. et al. Mechanisms and metabolic implications of regional differences among fat depots. *Cell Metab*. **17**, 644–656 (2013).
6. Trinh, N. T. et al. Increased expression of *EGR-1* in diabetic human adipose tissue-derived mesenchymal stem cells reduces their wound healing capacity. *Stem Cells Dev.* **25**, 760–773 (2016).
7. Sperandio, S. et al. The transcription factor Egr1 regulates the *HIF-1α* gene during hypoxia. *Mol. Carcinog.* **48**, 38–44 (2009).
8. Fernández-Real, J. M. et al. CD14 modulates inflammation-driven insulin resistance. *Diabetes* **60**, 2179–2186 (2011).
9. Chacón, M. R. et al. Expression of TWEAK and its receptor Fn14 in human subcutaneous adipose tissue. Relationship with other inflammatory cytokines in obesity. *Cytokine* **33**, 129–137 (2006).
10. Kleinert, M. et al. Animal models of obesity and diabetes mellitus. *Nat. Rev. Endocrinol.* **14**, 140–162 (2018).
11. McArthur, E. & Capra, J. A. Topologically associating domain boundaries that are stable across diverse cell types are evolutionarily constrained and enriched for heritability. *Am. J. Hum. Genet.* **108**, 269–283 (2021).
12. Qin, Y. F., Grimm, S. A., Roberts, J. D., Chrysovergis, K. & Wade, P. A. Alterations in promoter interaction landscape and transcriptional network underlying metabolic adaptation to diet. *Nat. Commun.* **11**, 962 (2020).
13. Choudhary, M. N. et al. Co-opted transposons help perpetuate conserved higher-order chromosomal structures. *Genome Biol.* **21**, 1–14 (2020).
14. Wu, H. J. et al. Topological isolation of developmental regulators in mammalian genomes. *Nat. Commun.* **12**, 4897 (2021).
15. Rhie, S. K. et al. Using 3D epigenomic maps of primary olfactory neuronal cells from living individuals to understand gene regulation. *Sci. Adv.* **4**, eaav8550 (2018).
16. Yang, Y. et al. Continuous-trait probabilistic model for comparing multi-species functional genomic data. *Cell Syst.* **7**, 208–218 (2018).
