# Supplementary material for: Dynamic chromatin architecture of the porcine adipose tissues with weight gain and loss
Source: Nat Commun. 2023 Jun 12;14:3457. doi: 10.1038/s41467-023-39191-0 (PMC10258790; doi:10.1038/s41467-023-39191-0)
Supplement: Supplementary file 3 — Description of Additional Supplementary Files [file 41467_2023_39191_MOESM3_ESM.pdf]

## Description of Additional Supplementary Files

File Name: Supplementary Data 1
Description: Data information for pig adipose tissues.

File Name: Supplementary Data 2
Description: Information for differentially expressed genes (DEGs) between ATs and groups.

File Name: Supplementary Data 3
Description: Compartment and TAD boundary frequency changes across ATs and groups.

File Name: Supplementary Data 4
Description: Information for CTCF-mediated loops.

File Name: Supplementary Data 5
Description: Genes with co-changes in expression level and PRS between ATs and groups.

File Name: Supplementary Data 6
Description: Data summary for Hi-C and RNA-seq data of seven species.

File Name: Supplementary Data 7
Description: Genomic and gene information for non-conserved states.

File Name: Supplementary Data 8
Description: Functional enrichment for genes embedded in different states. P values are calculated based on a one-sided accumulative hypergeometric test. q-values are calculated using the Banjamini-Hochberg procedure to account for multiple testings.

File Name: Supplementary Data 9
Description: Data summary for human Hi-C and RNA-seq data.

File Name: Supplementary Data 10
Description: Enhancer conservation for homologs between human and pig.

File Name: Supplementary Data 11
Description: Typical genes with conserved enhancers between human and pig.

File Name: Supplementary Data 12
Description: Enhancer conservation for single copy orthologs between human and pig.

File Name: Supplementary Data 13
Description: Functional enrichment for genes with expression changes between human and pig. P values are calculated based on a one-sided accumulative hypergeometric test. q-values are calculated using the Banjamini-Hochberg procedure to account for multiple testings.

File Name: Supplementary Data 14
Description: Enhancer conservation for typical expanded human gene families.
