# Supplementary material for: Dynamic chromatin architecture of the porcine adipose tissues with weight gain and loss
Source: Nat Commun. 2023 Jun 12;14:3457. doi: 10.1038/s41467-023-39191-0 (PMC10258790; doi:10.1038/s41467-023-39191-0)
Supplement: Supplementary file 18 — Reporting Summary [file 41467_2023_39191_MOESM18_ESM.pdf]

nature portfolio

Corresponding author(s): Mingzhou Li; Zhihua Zhang

Last updated by author(s): May 10, 2023

# Reporting Summary

Nature Portfolio wishes to improve the reproducibility of the work that we publish. This form provides structure for consistency and transparency in reporting. For further information on Nature Portfolio policies, see our Editorial Policies and the Editorial Policy Checklist.

## Statistics

For all statistical analyses, confirm that the following items are present in the figure legend, table legend, main text, or Methods section.

| n/a | Confirmed | |
|---|---|---|
| ☐ | ☒ | The exact sample size (*n*) for each experimental group/condition, given as a discrete number and unit of measurement |
| ☐ | ☒ | A statement on whether measurements were taken from distinct samples or whether the same sample was measured repeatedly |
| ☐ | ☒ | The statistical test(s) used AND whether they are one- or two-sided<br>*Only common tests should be described solely by name; describe more complex techniques in the Methods section.* |
| ☐ | ☒ | A description of all covariates tested |
| ☐ | ☒ | A description of any assumptions or corrections, such as tests of normality and adjustment for multiple comparisons |
| ☐ | ☒ | A full description of the statistical parameters including central tendency (e.g. means) or other basic estimates (e.g. regression coefficient) AND variation (e.g. standard deviation) or associated estimates of uncertainty (e.g. confidence intervals) |
| ☐ | ☒ | For null hypothesis testing, the test statistic (e.g. *F*, *t*, *r*) with confidence intervals, effect sizes, degrees of freedom and *P* value noted<br>*Give P values as exact values whenever suitable.* |
| ☒ | ☐ | For Bayesian analysis, information on the choice of priors and Markov chain Monte Carlo settings |
| ☐ | ☒ | For hierarchical and complex designs, identification of the appropriate level for tests and full reporting of outcomes |
| ☐ | ☒ | Estimates of effect sizes (e.g. Cohen's *d*, Pearson's *r*), indicating how they were calculated |

*Our web collection on statistics for biologists contains articles on many of the points above.*

## Software and code

Policy information about availability of computer code

**Data collection**

No software was used for data collection.

**Data analysis**

For Hi-C analysis:
Juicer pipeline (version 1.5.6), HiCRep (version 1.10.0, R package), miniMDS (version 2018-09-27, https://github. com/seqcode/miniMDS, python package), PyMOL (version 2.5.2), the public code of matrix2insulation.pl (version 1.0.0, https:// github.com/dekkerlab/cworld-dekker), PSYCHIC (version 2018-01-05, https://github.com/dhkron/PSYCHIC), deDoc (version 1.0.0, https://github.com/zengguangjie/deDoc2), FIMO (version 5.1.1), Fithic2 (version 2.0.7, https://github.com/ay-lab/fithic), bedtools (version v2.25.0), C-InterSecture (version 2020-09-14, https://github.com/NuriddinovMA/C-InterSecture), the custom codes of generate.oe.matrix.py (https://github.com/JiamanZhang/Lab_Porcine-Adiposes_paper_codes/tree/main/Lab_OE_matrix), BNBC (version 1.0.0, R package), the custom codes of get_ABindex.py (https://github.com/JiamanZhang/Lab_Porcine-Adiposes_paper_codes/tree/main/Lab_AB_compartment), R software (version 3.6.1), HOMER's findMotifs.pl (version v4.4, http://homer.ucsd.edu/homer/), UCSC LiftOver tool (version linux.x86_64), Pymol (version 2.5.2), Phylo-HMGP model (version 1)
For RNA-seq analysis:
STAR (version 2.6.0c), Kallisto (version 0.43.0), tximport (version 1.6.0, R package), CIBERSORTx (version 1.05, https:// cibersortx.stanford.edu/), edgeR (version 3.30.3, R package)
For ChIP-seq analysis:
BWA (version 0.7.15), Samtools (version 1.3.1), SICER tool (version 0.1.1, https://github.com/dariober/SICERpy), ROSE algorithm (version 0.1), MACS2 (version 2.1.1.20160309)
For identification of expansion and contraction genes:
CAFE (version 4.2.1, https://github.com/hahnlab/CAFE)

For functional enrichment analysis:
Metascape (version 3.5, http://metascape.org)

For manuscripts utilizing custom algorithms or software that are central to the research but not yet described in published literature, software must be made available to editors and reviewers. We strongly encourage code deposition in a community repository (e.g. GitHub). See the Nature Portfolio guidelines for submitting code & software for further information.

## Data

Policy information about availability of data

All manuscripts must include a data availability statement. This statement should provide the following information, where applicable:

- Accession codes, unique identifiers, or web links for publicly available datasets
- A description of any restrictions on data availability
- For clinical datasets or third party data, please ensure that the statement adheres to our policy

The reference genome and gene annotation file (Sscrofa11.1, release 102) were downloaded from Ensembl (https://ftp.ensembl.org/pub/release-102/). A phylogenetic tree of five species was obtained from the TimeTree database (http://timetree.org). The raw and processed Hi-C, RNA-seq, and ChIP-seq data of pigs generated in this study are available at Gene Expression Omnibus (GEO) under the accession code “GSE206539”. The raw Hi-C, RNA-seq, and ChIP-seq data of humans generated in this study are available at Genome Sequence Archive (GSA) under the accession code “HRA002514”. The public Hi-C and RNA-seq data of nine adipose samples of pigs were downloaded from Sequence Read Archive (SRA) under the BioProject accession codes “PRJNA637678” and “PRJNA733023”. The public Hi-C and RNA-seq data of three adipose samples of humans were downloaded from SRA under the BioProject accession code “PRJNA678123”. The public Hi-C and RNA-seq data of the other five species for cross-species analysis were downloaded from SRA under the BioProject accession codes “PRJNA637678” and “PRJNA817154”. Details are available in Supplementary Data 1, 6 and 9. Source data are provided with this paper.

## Human research participants

Policy information about studies involving human research participants and Sex and Gender in Research.

| | |
|---|---|
| Reporting on sex and gender | Our study only included male adipose tissue donors. Sexes were self-reported. |
| Population characteristics | Seven patients were recruited. Males with 20, 35, 36, 46, 47, 50 and 54 -year-old age, respectively. All of them are of Chinese descent. |
| Recruitment | All human adipose tissue samples were obtained with informed consent prior to tissue collection from healthy participants from Sichuan Provincial People's Hospital. There was no obvious bias in the selection. |
| Ethics oversight | Collection and sequencing of human clinical samples were approved by the Ethics Committee of Sichuan Provincial People's Hospital, and informed consent was obtained before the study. |

Note that full information on the approval of the study protocol must also be provided in the manuscript.

# Field-specific reporting

Please select the one below that is the best fit for your research. If you are not sure, read the appropriate sections before making your selection.

☒ Life sciences ☐ Behavioural & social sciences ☐ Ecological, evolutionary & environmental sciences

For a reference copy of the document with all sections, see nature.com/documents/nr-reporting-summary-flat.pdf

# Life sciences study design

All studies must disclose on these points even when the disclosure is negative.

| | |
|---|---|
| Sample size | No predetermined sample sizes were performed. Sample size was determined by sample availability at the time of analysis. All experiments were conducted in 1-10 or more biological replicates. Statistical testing ensured significant findings. |
| Data exclusions | No data were excluded from the study. |
| Replication | To identify regulatory mechanisms of three-dimensional (3D) genome architecture underlying obesity phenotypes, 249 Hi-C data (at least 10 biological replicates for each group), 239 RNA-seq data (at least 6 biological replicates for each group), and 73 ChIP-seq data (1-15 biological replicates for each group) of four adipose tissues (ATs) of a miniature pig model of weight gain/weight loss were used in this study. To explored the evolutionary patterns of local spatial context in SATs, 29 Hi-C data (7 biological replicates for Human, 2 biological replicates for Mouse, 2 biological replicates for Rabbit, 1 biological replicates for Cat, 2 biological replicates for Dog, 12 biological replicates for Pig, 3 biological replicates for Sheep) and their corresponding RNA-seq data were used in this study. To comprehensively compared PEI organization in human and pig genomes across the four homologous ATs, 25 Hi-C data (5-7 biological replicates for each tissue), 25 RNA-seq data (5-7 biological replicates for each tissue), and 10 ChIP-seq data (2-3 biological replicates for each tissue) of four human ATs were used in this study. |
| Randomization | Not relevant to our study since we did not use settings of experiment/control groups. |

| | |
|---|---|
| Blinding | Not relevant to our study since we did not use settings of experiment/control groups. |

# Reporting for specific materials, systems and methods

We require information from authors about some types of materials, experimental systems and methods used in many studies. Here, indicate whether each material, system or method listed is relevant to your study. If you are not sure if a list item applies to your research, read the appropriate section before selecting a response.

## Materials & experimental systems

| n/a | Involved in the study |
|---|---|
| ☐ | ☒ Antibodies |
| ☒ | ☐ Eukaryotic cell lines |
| ☒ | ☐ Palaeontology and archaeology |
| ☐ | ☒ Animals and other organisms |
| ☒ | ☐ Clinical data |
| ☒ | ☐ Dual use research of concern |

## Methods

| n/a | Involved in the study |
|---|---|
| ☐ | ☒ ChIP-seq |
| ☒ | ☐ Flow cytometry |
| ☒ | ☐ MRI-based neuroimaging |

# Antibodies

| | |
|---|---|
| Antibodies used | Antibody used for immunoprecipitation: H3K27ac (Abcam, Cat. #ab4729). |
| Validation | This antibody was extensively validated in previously published papers and by suppliers. This antibody has been previously used in our lab: Zhi M, Zhang J, Tang Q, et al. Generation and characterization of stable pig pregastrulation epiblast stem cell lines. Cell Res. 2022;32(4):383-400; Li D, Ning C, Zhang J, et al. Dynamic transcriptome and chromatin architecture in granulosa cells during chicken folliculogenesis. Nat Commun. 2022;13(1):131. |

# Animals and other research organisms

Policy information about studies involving animals; ARRIVE guidelines recommended for reporting animal research, and Sex and Gender in Research

| | |
|---|---|
| Laboratory animals | Pig (Bama pigs, female, 2-year-old) |
| Wild animals | No wild animals used in the study. |
| Reporting on sex | Sex was not considered in this study. All female animals. |
| Field-collected samples | No field-collected samples used in the study. |
| Ethics oversight | All the animals and samples used in this study were collected according to the guidelines for the care and use of experimental animals established by the Ministry of Agriculture of China. |

Note that full information on the approval of the study protocol must also be provided in the manuscript.

# ChIP-seq

## Data deposition

☒ Confirm that both raw and final processed data have been deposited in a public database such as GEO.

☒ Confirm that you have deposited or provided access to graph files (e.g. BED files) for the called peaks.

| | |
|---|---|
| Data access links<br>*May remain private before publication.* | For Human ChIP-seq data: Genome Sequence Archive (GSA) under the accession number HRA002514 (https://ngdc.cncb.ac.cn/gsa-human/browse/HRA002514).<br>For Pig ChIP-seq data: Gene Expression Omnibus (GEO) under the accession number GSE206539 (reviewer link: https://www.ncbi.nlm.nih.gov/geo/query/acc.cgi?acc=GSE206539, passwd: sdepusqopvuvdmd). |
| Files in database submission | IP.H3-ASA.H3K27ac.R1.clean.fq.gz; IP.H3-ASA.H3K27ac.R2.clean.fq.gz<br>IN.H3-ASA.input.R1.clean.fq.gz; IN.H3-ASA.input.R2.clean.fq.gz<br>IP.H3-MAD.H3K27ac.R1.clean.fq.gz; IP.H3-MAD.H3K27ac.R2.clean.fq.gz<br>IN.H3-MAD.input.R1.clean.fq.gz; IN.H3-MAD.input.R2.clean.fq.gz<br>IP.H3-RAD.H3K27ac.R1.clean.fq.gz; IP.H3-RAD.H3K27ac.R2.clean.fq.gz<br>IP.H5-ASA.H3K27ac.R1.clean.fq.gz; IP.H5-ASA.H3K27ac.R2.clean.fq.gz<br>IN.H5-ASA.input.R1.clean.fq.gz; IN.H5-ASA.input.R2.clean.fq.gz<br>IP.H5-GOM.H3K27ac.R1.clean.fq.gz; IP.H5-GOM.H3K27ac.R2.clean.fq.gz |

IN.H5-GOM.input.R1.clean.fq.gz; IN.H5-GOM.input.R2.clean.fq.gz
IP.H5-RAD.H3K27ac.R1.clean.fq.gz; IP.H5-RAD.H3K27ac.R2.clean.fq.gz
IN.H5-RAD.input.R1.clean.fq.gz; IN.H5-RAD.input.R2.clean.fq.gz
IP.H6-ASA.H3K27ac.R1.clean.fq.gz; IP.H6-ASA.H3K27ac.R2.clean.fq.gz
IN.H6-ASA.input.R1.clean.fq.gz; IN.H6-ASA.input.R2.clean.fq.gz
IP.H6-GOM.H3K27ac.R1.clean.fq.gz; IP.H6-GOM.H3K27ac.R2.clean.fq.gz
IN.H6-GOM.input.R1.clean.fq.gz; IN.H6-GOM.input.R2.clean.fq.gz
IP.H6-MAD.H3K27ac.R1.clean.fq.gz; IP.H6-MAD.H3K27ac.R2.clean.fq.gz
IN.H6-MAD.input.R1.clean.fq.gz; IN.H6-MAD.input.R2.clean.fq.gz
IP.H6-RAD.H3K27ac.R1.clean.fq.gz; IP.H6-RAD.H3K27ac.R2.clean.fq.gz
IN.NC_GOM.replicate_10.input.R1.clean.fq.gz; IN.NC_GOM.replicate_10.input.R2.clean.fq.gz
IN.NC_GOM.replicate_3.input.R1.clean.fq.gz; IN.NC_GOM.replicate_3.input.R2.clean.fq.gz
IN.NC_GOM.replicate_7.input.R1.clean.fq.gz; IN.NC_GOM.replicate_7.input.R2.clean.fq.gz
IN.NC_MAD.replicate_3.input.R1.clean.fq.gz; IN.NC_MAD.replicate_3.input.R2.clean.fq.gz
IN.NC_RAD.replicate_10.input.R1.clean.fq.gz; IN.NC_RAD.replicate_10.input.R2.clean.fq.gz
IN.NC_RAD.replicate_3.input.R1.clean.fq.gz; IN.NC_RAD.replicate_3.input.R2.clean.fq.gz
IN.NC_ULB.replicate_10.input.R1.clean.fq.gz; IN.NC_ULB.replicate_10.input.R2.clean.fq.gz
IN.NC_ULB.replicate_3.input.R1.clean.fq.gz; IN.NC_ULB.replicate_3.input.R2.clean.fq.gz
IN.NC_ULB.replicate_7.input.R1.clean.fq.gz; IN.NC_ULB.replicate_7.input.R2.clean.fq.gz
IN.WG_GOM.replicate_14.input.R1.clean.fq.gz; IN.WG_GOM.replicate_14.input.R2.clean.fq.gz
IN.WG_GOM.replicate_15.input.R1.clean.fq.gz; IN.WG_GOM.replicate_15.input.R2.clean.fq.gz
IN.WG_GOM.replicate_20.input.R1.clean.fq.gz; IN.WG_GOM.replicate_20.input.R2.clean.fq.gz
IN.WG_GOM.replicate_21.input.R1.clean.fq.gz; IN.WG_GOM.replicate_21.input.R2.clean.fq.gz
IN.WG_GOM.replicate_25.input.R1.clean.fq.gz; IN.WG_GOM.replicate_25.input.R2.clean.fq.gz
IN.WG_GOM.replicate_28.input.R1.clean.fq.gz; IN.WG_GOM.replicate_28.input.R2.clean.fq.gz
IN.WG_GOM.replicate_30.input.R1.clean.fq.gz; IN.WG_GOM.replicate_30.input.R2.clean.fq.gz
IN.WG_GOM.replicate_33.input.R1.clean.fq.gz; IN.WG_GOM.replicate_33.input.R2.clean.fq.gz
IN.WG_GOM.replicate_38.input.R1.clean.fq.gz; IN.WG_GOM.replicate_38.input.R2.clean.fq.gz
IN.WG_GOM.replicate_41.input.R1.clean.fq.gz; IN.WG_GOM.replicate_41.input.R2.clean.fq.gz
IN.WG_GOM.replicate_45.input.R1.clean.fq.gz; IN.WG_GOM.replicate_45.input.R2.clean.fq.gz
IN.WG_GOM.replicate_7.input.R1.clean.fq.gz; IN.WG_GOM.replicate_7.input.R2.clean.fq.gz
IN.WG_MAD.replicate_15.input.R1.clean.fq.gz; IN.WG_MAD.replicate_15.input.R2.clean.fq.gz
IN.WG_MAD.replicate_20.input.R1.clean.fq.gz; IN.WG_MAD.replicate_20.input.R2.clean.fq.gz
IN.WG_MAD.replicate_25.input.R1.clean.fq.gz; IN.WG_MAD.replicate_25.input.R2.clean.fq.gz
IN.WG_MAD.replicate_28.input.R1.clean.fq.gz; IN.WG_MAD.replicate_28.input.R2.clean.fq.gz
IN.WG_MAD.replicate_30.input.R1.clean.fq.gz; IN.WG_MAD.replicate_30.input.R2.clean.fq.gz
IN.WG_MAD.replicate_38.input.R1.clean.fq.gz; IN.WG_MAD.replicate_38.input.R2.clean.fq.gz
IN.WG_MAD.replicate_42.input.R1.clean.fq.gz; IN.WG_MAD.replicate_42.input.R2.clean.fq.gz
IN.WG_MAD.replicate_43.input.R1.clean.fq.gz; IN.WG_MAD.replicate_43.input.R2.clean.fq.gz
IN.WG_MAD.replicate_45.input.R1.clean.fq.gz; IN.WG_MAD.replicate_45.input.R2.clean.fq.gz
IN.WG_MAD.replicate_46.input.R1.clean.fq.gz; IN.WG_MAD.replicate_46.input.R2.clean.fq.gz
IN.WG_RAD.replicate_21.input.R1.clean.fq.gz; IN.WG_RAD.replicate_21.input.R2.clean.fq.gz
IN.WG_RAD.replicate_25.input.R1.clean.fq.gz; IN.WG_RAD.replicate_25.input.R2.clean.fq.gz
IN.WG_RAD.replicate_28.input.R1.clean.fq.gz; IN.WG_RAD.replicate_28.input.R2.clean.fq.gz
IN.WG_RAD.replicate_30.input.R1.clean.fq.gz; IN.WG_RAD.replicate_30.input.R2.clean.fq.gz
IN.WG_RAD.replicate_42.input.R1.clean.fq.gz; IN.WG_RAD.replicate_42.input.R2.clean.fq.gz
IN.WG_RAD.replicate_45.input.R1.clean.fq.gz; IN.WG_RAD.replicate_45.input.R2.clean.fq.gz
IN.WG_RAD.replicate_46.input.R1.clean.fq.gz; IN.WG_RAD.replicate_46.input.R2.clean.fq.gz
IN.WG_ULB.replicate_15.input.R1.clean.fq.gz; IN.WG_ULB.replicate_15.input.R2.clean.fq.gz
IN.WG_ULB.replicate_25.input.R1.clean.fq.gz; IN.WG_ULB.replicate_25.input.R2.clean.fq.gz
IN.WG_ULB.replicate_44.input.R1.clean.fq.gz; IN.WG_ULB.replicate_44.input.R2.clean.fq.gz
IN.WG_ULB.replicate_45.input.R1.clean.fq.gz; IN.WG_ULB.replicate_45.input.R2.clean.fq.gz
IN.WL_GOM.replicate_1.input.R1.clean.fq.gz; IN.WL_GOM.replicate_1.input.R2.clean.fq.gz
IN.WL_GOM.replicate_4.input.R1.clean.fq.gz; IN.WL_GOM.replicate_4.input.R2.clean.fq.gz
IN.WL_MAD.replicate_5.input.R1.clean.fq.gz; IN.WL_MAD.replicate_5.input.R2.clean.fq.gz
IN.WL_RAD.replicate_1.input.R1.clean.fq.gz; IN.WL_RAD.replicate_1.input.R2.clean.fq.gz
IN.WL_RAD.replicate_5.input.R1.clean.fq.gz; IN.WL_RAD.replicate_5.input.R2.clean.fq.gz
IN.WL_RAD.replicate_7.input.R1.clean.fq.gz; IN.WL_RAD.replicate_7.input.R2.clean.fq.gz
IN.WL_ULB.replicate_5.input.R1.clean.fq.gz; IN.WL_ULB.replicate_5.input.R2.clean.fq.gz
IN.WL_ULB.replicate_7.input.R1.clean.fq.gz; IN.WL_ULB.replicate_7.input.R2.clean.fq.gz
IP.NC_GOM.replicate_10.H3K27ac.R1.clean.fq.gz; IP.NC_GOM.replicate_10.H3K27ac.R2.clean.fq.gz
IP.NC_GOM.replicate_3.H3K27ac.R1.clean.fq.gz; IP.NC_GOM.replicate_3.H3K27ac.R2.clean.fq.gz
IP.NC_GOM.replicate_7.H3K27ac.R1.clean.fq.gz; IP.NC_GOM.replicate_7.H3K27ac.R2.clean.fq.gz
IP.NC_MAD.replicate_3.H3K27ac.R1.clean.fq.gz; IP.NC_MAD.replicate_3.H3K27ac.R2.clean.fq.gz
IP.NC_RAD.replicate_10.H3K27ac.R1.clean.fq.gz; IP.NC_RAD.replicate_10.H3K27ac.R2.clean.fq.gz
IP.NC_RAD.replicate_3.H3K27ac.R1.clean.fq.gz; IP.NC_RAD.replicate_3.H3K27ac.R2.clean.fq.gz
IP.NC_ULB.replicate_10.H3K27ac.R1.clean.fq.gz; IP.NC_ULB.replicate_10.H3K27ac.R2.clean.fq.gz
IP.NC_ULB.replicate_3.H3K27ac.R1.clean.fq.gz; IP.NC_ULB.replicate_3.H3K27ac.R2.clean.fq.gz
IP.NC_ULB.replicate_7.H3K27ac.R1.clean.fq.gz; IP.NC_ULB.replicate_7.H3K27ac.R2.clean.fq.gz
IP.WG_GOM.replicate_14.H3K27ac.R1.clean.fq.gz; IP.WG_GOM.replicate_14.H3K27ac.R2.clean.fq.gz
IP.WG_GOM.replicate_15.H3K27ac.R1.clean.fq.gz; IP.WG_GOM.replicate_15.H3K27ac.R2.clean.fq.gz
IP.WG_GOM.replicate_20.H3K27ac.R1.clean.fq.gz; IP.WG_GOM.replicate_20.H3K27ac.R2.clean.fq.gz
IP.WG_GOM.replicate_21.H3K27ac.R1.clean.fq.gz; IP.WG_GOM.replicate_21.H3K27ac.R2.clean.fq.gz
IP.WG_GOM.replicate_25.H3K27ac.R1.clean.fq.gz; IP.WG_GOM.replicate_25.H3K27ac.R2.clean.fq.gz
IP.WG_GOM.replicate_28.H3K27ac.R1.clean.fq.gz; IP.WG_GOM.replicate_28.H3K27ac.R2.clean.fq.gz
IP.WG_GOM.replicate_30.H3K27ac.R1.clean.fq.gz; IP.WG_GOM.replicate_30.H3K27ac.R2.clean.fq.gz

IP.WG_GOM.replicate_33.H3K27ac.R1.clean.fq.gz; IP.WG_GOM.replicate_33.H3K27ac.R2.clean.fq.gz
IP.WG_GOM.replicate_38.H3K27ac.R1.clean.fq.gz; IP.WG_GOM.replicate_38.H3K27ac.R2.clean.fq.gz
IP.WG_GOM.replicate_41.H3K27ac.R1.clean.fq.gz; IP.WG_GOM.replicate_41.H3K27ac.R2.clean.fq.gz
IP.WG_GOM.replicate_42.H3K27ac.R1.clean.fq.gz; IP.WG_GOM.replicate_42.H3K27ac.R2.clean.fq.gz
IP.WG_GOM.replicate_43.H3K27ac.R1.clean.fq.gz; IP.WG_GOM.replicate_43.H3K27ac.R2.clean.fq.gz
IP.WG_GOM.replicate_45.H3K27ac.R1.clean.fq.gz; IP.WG_GOM.replicate_45.H3K27ac.R2.clean.fq.gz
IP.WG_GOM.replicate_46.H3K27ac.R1.clean.fq.gz; IP.WG_GOM.replicate_46.H3K27ac.R2.clean.fq.gz
IP.WG_GOM.replicate_7.H3K27ac.R1.clean.fq.gz; IP.WG_GOM.replicate_7.H3K27ac.R2.clean.fq.gz
IP.WG_MAD.replicate_14.H3K27ac.R1.clean.fq.gz; IP.WG_MAD.replicate_14.H3K27ac.R2.clean.fq.gz
IP.WG_MAD.replicate_20.H3K27ac.R1.clean.fq.gz; IP.WG_MAD.replicate_20.H3K27ac.R2.clean.fq.gz
IP.WG_MAD.replicate_21.H3K27ac.R1.clean.fq.gz; IP.WG_MAD.replicate_21.H3K27ac.R2.clean.fq.gz
IP.WG_MAD.replicate_25.H3K27ac.R1.clean.fq.gz; IP.WG_MAD.replicate_25.H3K27ac.R2.clean.fq.gz
IP.WG_MAD.replicate_28.H3K27ac.R1.clean.fq.gz; IP.WG_MAD.replicate_28.H3K27ac.R2.clean.fq.gz
IP.WG_MAD.replicate_30.H3K27ac.R1.clean.fq.gz; IP.WG_MAD.replicate_30.H3K27ac.R2.clean.fq.gz
IP.WG_MAD.replicate_33.H3K27ac.R1.clean.fq.gz; IP.WG_MAD.replicate_33.H3K27ac.R2.clean.fq.gz
IP.WG_MAD.replicate_41.H3K27ac.R1.clean.fq.gz; IP.WG_MAD.replicate_41.H3K27ac.R2.clean.fq.gz
IP.WG_MAD.replicate_42.H3K27ac.R1.clean.fq.gz; IP.WG_MAD.replicate_42.H3K27ac.R2.clean.fq.gz
IP.WG_MAD.replicate_43.H3K27ac.R1.clean.fq.gz; IP.WG_MAD.replicate_43.H3K27ac.R2.clean.fq.gz
IP.WG_MAD.replicate_44.H3K27ac.R1.clean.fq.gz; IP.WG_MAD.replicate_44.H3K27ac.R2.clean.fq.gz
IP.WG_MAD.replicate_45.H3K27ac.R1.clean.fq.gz; IP.WG_MAD.replicate_45.H3K27ac.R2.clean.fq.gz
IP.WG_MAD.replicate_46.H3K27ac.R1.clean.fq.gz; IP.WG_MAD.replicate_46.H3K27ac.R2.clean.fq.gz
IP.WG_MAD.replicate_7.H3K27ac.R1.clean.fq.gz; IP.WG_MAD.replicate_7.H3K27ac.R2.clean.fq.gz
IP.WG_RAD.replicate_14.H3K27ac.R1.clean.fq.gz; IP.WG_RAD.replicate_14.H3K27ac.R2.clean.fq.gz
IP.WG_RAD.replicate_15.H3K27ac.R1.clean.fq.gz; IP.WG_RAD.replicate_15.H3K27ac.R2.clean.fq.gz
IP.WG_RAD.replicate_21.H3K27ac.R1.clean.fq.gz; IP.WG_RAD.replicate_21.H3K27ac.R2.clean.fq.gz
IP.WG_RAD.replicate_25.H3K27ac.R1.clean.fq.gz; IP.WG_RAD.replicate_25.H3K27ac.R2.clean.fq.gz
IP.WG_RAD.replicate_28.H3K27ac.R1.clean.fq.gz; IP.WG_RAD.replicate_28.H3K27ac.R2.clean.fq.gz
IP.WG_RAD.replicate_30.H3K27ac.R1.clean.fq.gz; IP.WG_RAD.replicate_30.H3K27ac.R2.clean.fq.gz
IP.WG_RAD.replicate_38.H3K27ac.R1.clean.fq.gz; IP.WG_RAD.replicate_38.H3K27ac.R2.clean.fq.gz
IP.WG_RAD.replicate_42.H3K27ac.R1.clean.fq.gz; IP.WG_RAD.replicate_42.H3K27ac.R2.clean.fq.gz
IP.WG_RAD.replicate_43.H3K27ac.R1.clean.fq.gz; IP.WG_RAD.replicate_43.H3K27ac.R2.clean.fq.gz
IP.WG_RAD.replicate_44.H3K27ac.R1.clean.fq.gz; IP.WG_RAD.replicate_44.H3K27ac.R2.clean.fq.gz
IP.WG_RAD.replicate_45.H3K27ac.R1.clean.fq.gz; IP.WG_RAD.replicate_45.H3K27ac.R2.clean.fq.gz
IP.WG_RAD.replicate_46.H3K27ac.R1.clean.fq.gz; IP.WG_RAD.replicate_46.H3K27ac.R2.clean.fq.gz
IP.WG_ULB.replicate_15.H3K27ac.R1.clean.fq.gz; IP.WG_ULB.replicate_15.H3K27ac.R2.clean.fq.gz
IP.WG_ULB.replicate_20.H3K27ac.R1.clean.fq.gz; IP.WG_ULB.replicate_20.H3K27ac.R2.clean.fq.gz
IP.WG_ULB.replicate_21.H3K27ac.R1.clean.fq.gz; IP.WG_ULB.replicate_21.H3K27ac.R2.clean.fq.gz
IP.WG_ULB.replicate_25.H3K27ac.R1.clean.fq.gz; IP.WG_ULB.replicate_25.H3K27ac.R2.clean.fq.gz
IP.WG_ULB.replicate_30.H3K27ac.R1.clean.fq.gz; IP.WG_ULB.replicate_30.H3K27ac.R2.clean.fq.gz
IP.WG_ULB.replicate_38.H3K27ac.R1.clean.fq.gz; IP.WG_ULB.replicate_38.H3K27ac.R2.clean.fq.gz
IP.WG_ULB.replicate_41.H3K27ac.R1.clean.fq.gz; IP.WG_ULB.replicate_41.H3K27ac.R2.clean.fq.gz
IP.WG_ULB.replicate_43.H3K27ac.R1.clean.fq.gz; IP.WG_ULB.replicate_43.H3K27ac.R2.clean.fq.gz
IP.WG_ULB.replicate_44.H3K27ac.R1.clean.fq.gz; IP.WG_ULB.replicate_44.H3K27ac.R2.clean.fq.gz
IP.WG_ULB.replicate_45.H3K27ac.R1.clean.fq.gz; IP.WG_ULB.replicate_45.H3K27ac.R2.clean.fq.gz
IP.WL_GOM.replicate_1.H3K27ac.R1.clean.fq.gz; IP.WL_GOM.replicate_1.H3K27ac.R2.clean.fq.gz
IP.WL_GOM.replicate_4.H3K27ac.R1.clean.fq.gz; IP.WL_GOM.replicate_4.H3K27ac.R2.clean.fq.gz
IP.WL_GOM.replicate_5.H3K27ac.R1.clean.fq.gz; IP.WL_GOM.replicate_5.H3K27ac.R2.clean.fq.gz
IP.WL_GOM.replicate_7.H3K27ac.R1.clean.fq.gz; IP.WL_GOM.replicate_7.H3K27ac.R2.clean.fq.gz
IP.WL_MAD.replicate_4.H3K27ac.R1.clean.fq.gz; IP.WL_MAD.replicate_4.H3K27ac.R2.clean.fq.gz
IP.WL_MAD.replicate_5.H3K27ac.R1.clean.fq.gz; IP.WL_MAD.replicate_5.H3K27ac.R2.clean.fq.gz
IP.WL_MAD.replicate_7.H3K27ac.R1.clean.fq.gz; IP.WL_MAD.replicate_7.H3K27ac.R2.clean.fq.gz
IP.WL_RAD.replicate_1.H3K27ac.R1.clean.fq.gz; IP.WL_RAD.replicate_1.H3K27ac.R2.clean.fq.gz
IP.WL_RAD.replicate_4.H3K27ac.R1.clean.fq.gz; IP.WL_RAD.replicate_4.H3K27ac.R2.clean.fq.gz
IP.WL_RAD.replicate_5.H3K27ac.R1.clean.fq.gz; IP.WL_RAD.replicate_5.H3K27ac.R2.clean.fq.gz
IP.WL_RAD.replicate_7.H3K27ac.R1.clean.fq.gz; IP.WL_RAD.replicate_7.H3K27ac.R2.clean.fq.gz
IP.WL_ULB.replicate_5.H3K27ac.R1.clean.fq.gz; IP.WL_ULB.replicate_5.H3K27ac.R2.clean.fq.gz
IP.WL_ULB.replicate_7.H3K27ac.R1.clean.fq.gz; IP.WL_ULB.replicate_7.H3K27ac.R2.clean.fq.gz

Genome browser session
(e.g. UCSC)

Our publicly available online resource (https://3dgphat.sicau.edu.cn/HPC/front/#/genomeBrowser).

## Methodology

Replicates

For Human:
Four adipose tissues (GOM, MAD, RAD, and ASA) with 2-3 biological replicates for each adipose tissue.
For Pig:
Four adipose tissues (GOM, MAD, RAD, and ULB) with 1-3 biological replicates for each adipose tissue in NC group.
Four adipose tissues (GOM, MAD, RAD, and ULB) with 10-15 biological replicates for each adipose tissue in WG group.
Four adipose tissues (GOM, MAD, RAD, and ULB) with 2-4 biological replicates for each adipose tissue in WL group.

Sequencing depth

All ChIP-seq data were 150 bp paired-end reads. For pig, there were averaged ~108 M (million) reads and ~79 M of uniquely mapped reads for each sample. For human, there were ~105 M reads and ~78 M of uniquely mapped reads for each sample.

Antibodies

Antibody used for immunoprecipitation: H3K27ac (Abcam, Cat. #ab4729).

| | |
|---|---|
| Peak calling parameters | SICER tool: --windowSize 200 --gapSize 3 --mapq 0 --fragSize 250 --FDR 0.05 |
| Data quality | We performed the peak calling step for each sample bam file, and group bam files were pulled from all replicates. Peaks called from group bam files were retained if the peak was observed in at least 50% of all replicates and the overlap length was at least 50% of both pairwise peaks. |
| Software | BWA (version 0.7.15), Samtools (version 1.3.1), SICER tool (version 0.1.1, https://github.com/dariober/SICERpy), ROSE algorithm (version 0.1), MACS2 (version 2.1.1.20160309) |
